# Supplementary material for: In silico detection of dysregulated genes and molecular pathways in Alzheimer’s disease as basis for food restoring approach
Source: PeerJ. 2025 Apr 7;13:e19100. doi: 10.7717/peerj.19100 (PMC11984471; doi:10.7717/peerj.19100)
Supplement: Supplemental Information 4 — The target genes of up-regulated miRs in AD. The most significantly altered genes, selected based on their p-value, were further analyzed using the Reactome online tool. [file peerj-13-19100-s004.docx]

| **Genes microRNA-UP** | |
| --- | --- |
| **Gene Symbol** | **p-value** |
| BAHD1 | 7,03152E-05 |
| DHX40 | 0,00012793 |
| NEO1 | 0,000204943 |
| MMD | 0,000299692 |
| SSX2IP | 0,000391314 |
| ADAMTS9 | 0,000407773 |
| LRRC8B | 0,00047758 |
| ARRDC4 | 0,000486026 |
| GRHL1 | 0,000486026 |
| C16orf52 | 0,000605904 |
| A1CF | 0,000993297 |
| CDR2L | 0,000993297 |
| RNF144A | 0,000993297 |
| MIER3 | 0,001061784 |
