# Supplementary material for: In silico detection of dysregulated genes and molecular pathways in Alzheimer’s disease as basis for food restoring approach
Source: PeerJ. 2025 Apr 7;13:e19100. doi: 10.7717/peerj.19100 (PMC11984471; doi:10.7717/peerj.19100)
Supplement: Supplemental Information 5 — The target genes of down-regulated miRNAs in AD. The genes showing the most significant alterations, identified by their p-values, were subjected to further analysis using the Reactome online tool. [file peerj-13-19100-s005.docx]

| **Genes microRNA-DOWN** | |
| --- | --- |
| **Gene Symbol** | **p-value** |
| FRAT2 | 1,96779E-05 |
| TIGD5 | 1,96779E-05 |
| ZBTB37 | 2,8865E-05 |
| MTMR10 | 4,72848E-05 |
| SMS | 4,72848E-05 |
| PDIK1L | 9,28602E-05 |
| PRR14L | 9,8807E-05 |
| ICOS | 0,000108 |
| DCUN1D4 | 0,000114165 |
| SEC31B | 0,000121977 |
| ATP11C | 0,000129872 |
| PPP2R5E | 0,000142776 |
| ADAMTS18 | 0,000201986 |
| PDK4 | 0,000201986 |
| POM121C | 0,000201986 |
| STAG1 | 0,000201986 |
| AMMECR1 | 0,000213971 |
| CNNM2 | 0,000218532 |
| SOX5 | 0,000265761 |
| ZBTB16 | 0,000265761 |
| DLG2 | 0,000286272 |
| JARID2 | 0,000286272 |
| RUNX1T1 | 0,000297646 |
| CDK8 | 0,000301684 |
| EXOC5 | 0,000313315 |
| DAPK1 | 0,000345726 |
| ZCCHC2 | 0,000349672 |
| MMS19 | 0,000370252 |
| SLC25A37 | 0,000370252 |
| TNFAIP3 | 0,000370252 |
| FOXP1 | 0,000383442 |
| CHD9 | 0,000449206 |
| ADAMTS5 | 0,000481161 |
| STOX2 | 0,000482341 |
| EPC1 | 0,000570743 |
| EMX2 | 0,00059839 |
| SLC2A14 | 0,00059839 |
| PCGF3 | 0,000631735 |
| SYT2 | 0,000631735 |
| LRP4 | 0,000636128 |
| TRIB2 | 0,000636128 |
| OTUD4 | 0,000830089 |
| AQP11 | 0,000843128 |
| AZI2 | 0,000843128 |
| GOLM1 | 0,000843128 |
| SIPA1L2 | 0,000845295 |
| TGFBR3 | 0,000847844 |
| RAB30 | 0,000872844 |
| CAMSAP2 | 0,000914051 |
| LIN28B | 0,000914051 |
| SERBP1 | 0,000914051 |
| TGFB2 | 0,000914051 |
| PTEN | 0,000974304 |
| RNF38 | 0,001021278 |
