# Supplementary material for: In silico detection of dysregulated genes and molecular pathways in Alzheimer’s disease as basis for food restoring approach
Source: PeerJ. 2025 Apr 7;13:e19100. doi: 10.7717/peerj.19100 (PMC11984471; doi:10.7717/peerj.19100)
Supplement: Supplemental Information 8 — The following table shows the 25 most relevant pathways sorted by p-value, related to the target gene of down-regulated miRs in AD. [file peerj-13-19100-s008.pdf]

Pathway Analysis Report

This report contains the pathway analysis results for the submitted sample ''. Analysis was performed against Reactome version 75 on 20/03/2021. The web link to these results is:

https://reactome.org/PathwayBrowser/#/ANALYSIS=MjAyMTAzMTYxNjIwMTRfNTI0ODA%3D

Please keep in mind that analysis results are temporarily stored on our server. The storage period depends on usage of the service but is at least 7 days. As a result, please note that this URL is only valid for a limited time period and it might have expired.

Table of Contents

1. Introduction
2. Properties
3. Genome-wide overview
4. Most significant pathways
5. Pathways details
6. Identifiers found
7. Identifiers not found

1. Introduction

Reactome is a curated database of pathways and reactions in human biology. Reactions can be considered as pathway 'steps'. Reactome defines a 'reaction' as any event in biology that changes the state of a biological molecule. Binding, activation, translocation, degradation and classical biochemical events involving a catalyst are all reactions. Information in the database is authored by expert biologists, entered and maintained by Reactome's team of curators and editorial staff. Reactome content frequently cross-references other resources e.g. NCBI, Ensembl, UniProt, KEGG (Gene and Compound), ChEBI, PubMed and GO. Orthologous reactions inferred from annotation for Homo sapiens are available for 17 non-human species including mouse, rat, chicken, puffer fish, worm, fly, yeast, rice, and Arabidopsis. Pathways are represented by simple diagrams following an SBGN-like format.

Reactome's annotated data describe reactions possible if all annotated proteins and small molecules were present and active simultaneously in a cell. By overlaying an experimental dataset on these annotations, a user can perform a pathway over-representation analysis. By overlaying quantitative expression data or time series, a user can visualize the extent of change in affected pathways and its progression. A binomial test is used to calculate the probability shown for each result, and the p-values are corrected for the multiple testing (Benjamini–Hochberg procedure) that arises from evaluating the submitted list of identifiers against every pathway.

To learn more about our Pathway Analysis, please have a look at our relevant publications:

Fabregat A, Sidiropoulos K, Garapati P, Gillespie M, Hausmann K, Haw R, … D'Eustachio P (2016). The reactome pathway knowledgebase. Nucleic Acids Research, 44(D1), D481–D487. https://doi.org/10.1093/nar/gkv1351.

Fabregat A, Sidiropoulos K, Viteri G, Forner O, Marin-Garcia P, Arnau V, … Hermjakob H (2017). Reactome pathway analysis: a high-performance in-memory approach. BMC Bioinformatics, 18.

2. Properties

- This is an **overrepresentation** analysis: A statistical (hypergeometric distribution) test that determines whether certain Reactome pathways are over-represented (enriched) in the submitted data. It answers the question 'Does my list contain more proteins for pathway X than would be expected by chance?' This test produces a probability score, which is corrected for false discovery rate using the Benjamani-Hochberg method.
- 33 out of 54 identifiers in the sample were found in Reactome, where 315 pathways were hit by at least one of them.
- All non-human identifiers have been converted to their human equivalent.
- This report is filtered to show only results for species 'Homo sapiens' and resource 'all resources'.
- The unique ID for this analysis (token) is MjAyMTAzMTYxNjIwMTRfNTI0ODA%3D. This ID is valid for at least 7 days in Reactome's server. Use it to access Reactome services with your data.

3. Genome-wide overview

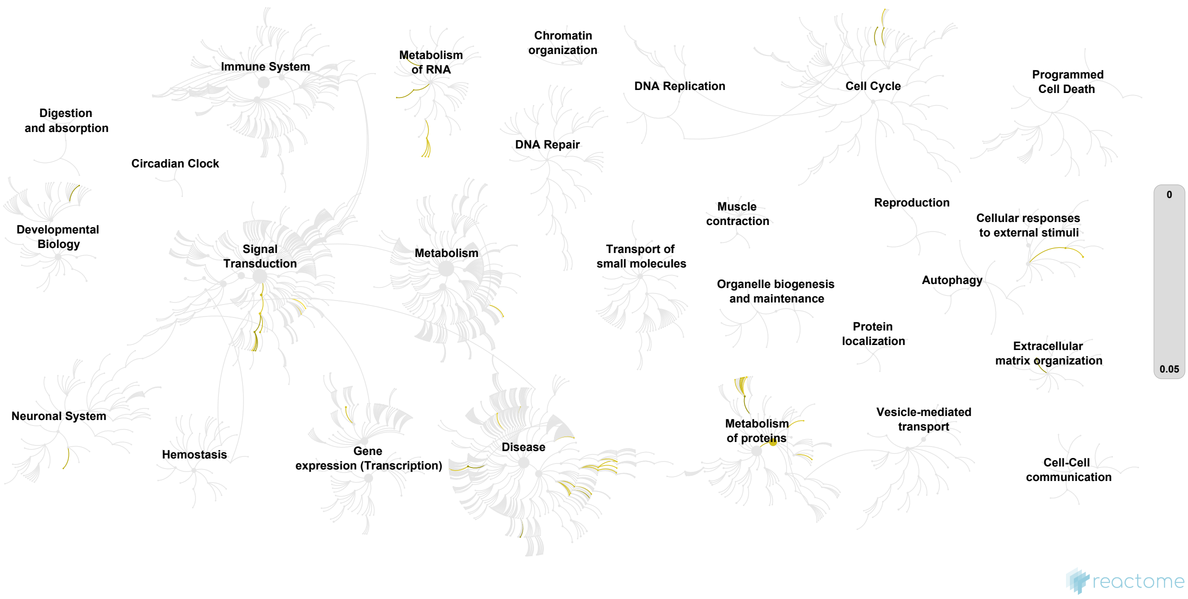

This figure shows a genome-wide overview of the results of your pathway analysis. Reactome pathways are arranged in a hierarchy. The center of each of the circular "bursts" is the root of one top-level pathway, for example "DNA Repair". Each step away from the center represents the next level lower in the pathway hierarchy. The color code denotes over-representation of that pathway in your input dataset. Light grey signifies pathways which are not significantly over-represented.

4. Most significant pathways

The following table shows the 25 most relevant pathways sorted by p-value.

Pathway name	Entities				Reactions	
	found	ratio	p-value	FDR*	found	ratio
Beta-catenin phosphorylation cascade	2 / 19	0.001	0.003	0.186	4 / 4	3.02e-04
SUMOylation of DNA damage response and repair proteins	3 / 81	0.006	0.006	0.186	5 / 24	0.002
Regulation of PTEN mRNA translation	2 / 29	0.002	0.007	0.186	13 / 24	0.002
Disassembly of the destruction complex and recruitment of AXIN to the membrane	2 / 33	0.002	0.01	0.186	3 / 7	5.29e-04
Defective TPR may confer susceptibility towards thyroid papillary carcinoma (TPC)	2 / 36	0.002	0.011	0.186	1 / 1	7.56e-05
Transport of the SLBP independent Mature mRNA	2 / 39	0.003	0.013	0.186	3 / 3	2.27e-04
Defective B3GALTL causes Peters-plus syndrome (PpS)	2 / 39	0.003	0.013	0.186	1 / 1	7.56e-05
SUMOylation of SUMOylation proteins	2 / 39	0.003	0.013	0.186	2 / 5	3.78e-04
PTEN Loss of Function in Cancer	1 / 3	2.04e-04	0.013	0.186	1 / 1	7.56e-05
PIP3 activates AKT signaling	5 / 319	0.022	0.013	0.186	45 / 86	0.007
Transport of the SLBP Dependant Mature mRNA	2 / 40	0.003	0.014	0.186	3 / 3	2.27e-04
Regulation of Glucokinase by Glucokinase Regulatory Protein	2 / 40	0.003	0.014	0.186	1 / 5	3.78e-04
Regulation of HSF1-mediated heat shock response	3 / 113	0.008	0.014	0.186	7 / 14	0.001
O-glycosylation of TSR domain-containing proteins	2 / 41	0.003	0.014	0.186	2 / 2	1.51e-04
Nuclear import of Rev protein	2 / 41	0.003	0.014	0.186	2 / 7	5.29e-04
Nuclear Pore Complex (NPC) Disassembly	2 / 42	0.003	0.015	0.186	2 / 2	1.51e-04
Ovarian tumor domain proteases	2 / 43	0.003	0.016	0.186	7 / 14	0.001
SUMOylation of ubiquitinylation proteins	2 / 43	0.003	0.016	0.186	1 / 3	2.27e-04
Vpr-mediated nuclear import of PICs	2 / 44	0.003	0.016	0.186	1 / 2	1.51e-04
Rev-mediated nuclear export of HIV RNA	2 / 44	0.003	0.016	0.186	2 / 10	7.56e-04
Transport of Mature mRNA Derived from an Intronless Transcript	2 / 46	0.003	0.018	0.186	3 / 3	2.27e-04

Pathway name	Entities				Reactions	
	found	ratio	p-value	FDR*	found	ratio
Interactions of Rev with host cellular proteins	2 / 46	0.003	0.018	0.186	4 / 16	0.001
NS1 Mediated Effects on Host Pathways	2 / 46	0.003	0.018	0.186	1 / 6	4.54e-04
TP53 Regulates Metabolic Genes	3 / 125	0.008	0.018	0.186	4 / 34	0.003
Transport of Mature mRNAs Derived from Intronless Transcripts	2 / 47	0.003	0.019	0.186	9 / 9	6.80e-04

* False Discovery Rate

5. Pathways details

For every pathway of the most significant pathways, we present its diagram, as well as a short summary, its bibliography and the list of inputs found in it.

1. Beta-catenin phosphorylation cascade (R-HSA-196299)

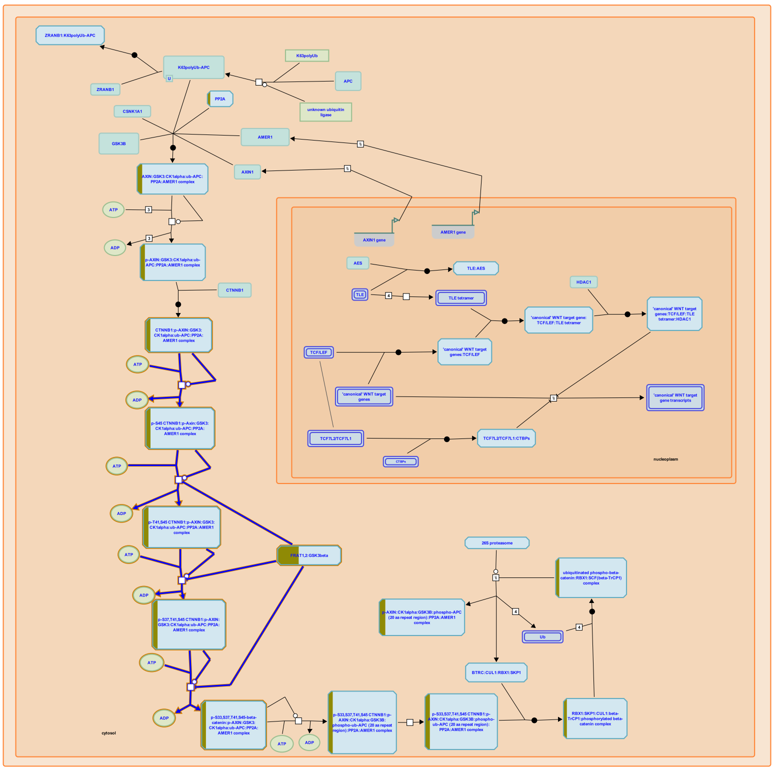

Cellular compartments: cytosol.

Degradation of beta-catenin is initiated following amino-terminal serine/threonine phosphorylation. Phosphorylation of B-catenin at S45 by CK1 alpha primes the subsequent sequential GSK-3-mediated phosphorylation at Thr41, Ser37 and Ser33 (Amit et al., 2002 ; Lui et al., 2002).

References

Liu C, Li Y, Semenov M, Han C, Baeg GH, Tan Y, ... He X (2002). Control of beta-catenin phosphorylation/degradation by a dual-kinase mechanism. Cell, 108, 837-47.

Amit S, Hatzubai A, Birman Y, Andersen JS, Ben-Shushan E, Mann M, ... Alkalay I (2002). Axin-mediated CKI phosphorylation of beta-catenin at Ser 45: a molecular switch for the Wnt pathway. Genes Dev, 16, 1066-76.

Edit history

Date	Action	Author
2007-04-03	Authored	Kimelman D
2007-04-19	Edited	Matthews L
2007-04-19	Created	Matthews L

Date	Action	Author
2007-04-27	Reviewed	Pagano M
2020-11-24	Modified	Shorser S

Entities found in this pathway (2)

Input	UniProt Id	Input	UniProt Id
FRAT2	O75474	PPP2R5E	Q16537

2. SUMOylation of DNA damage response and repair proteins (R-HSA-3108214)

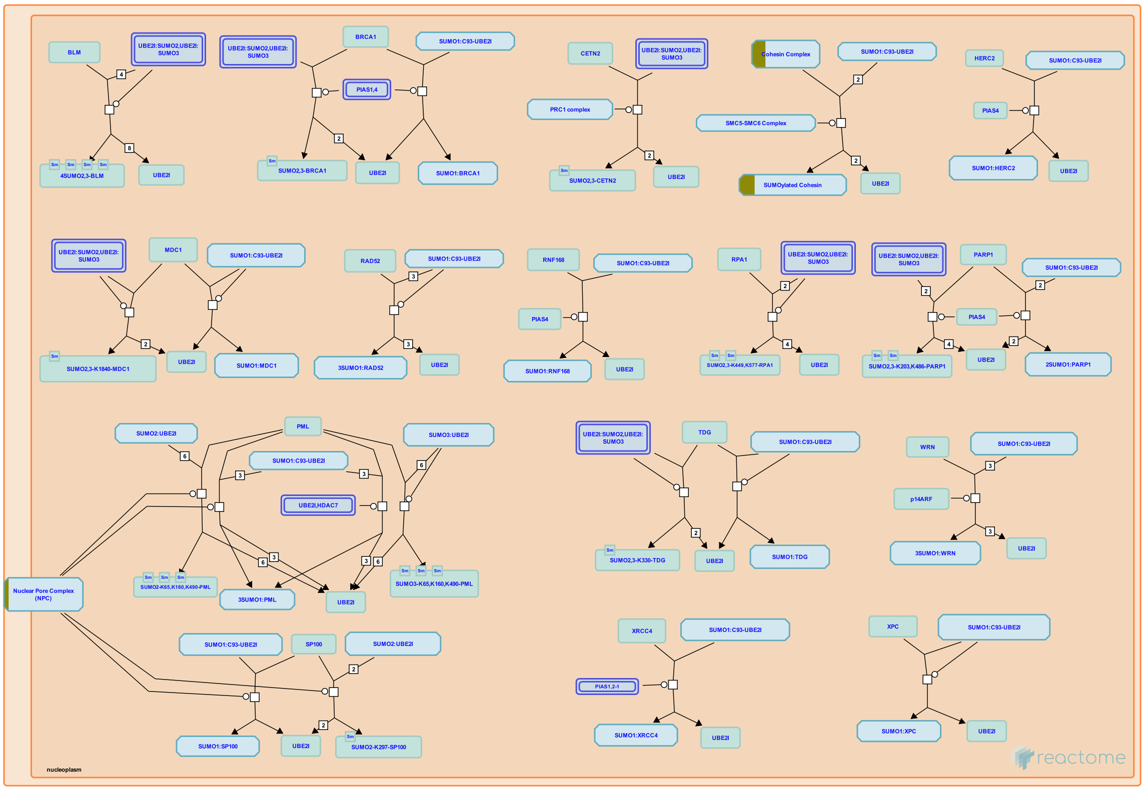

Cellular compartments: nucleoplasm.

Several factors that participate in DNA damage response and repair are SUMOylated (reviewed in Dou et al. 2011, Bekker-Jensen and Mailand 2011, Ulrich 2012, Psakhye and Jentsch 2012, Bologna and Ferrari 2013, Flotho and Melchior 2013, Jackson and Durocher 2013). SUMOylation can alter enzymatic activity and protein stability or it can serve to recruit additional factors. For example, SUMOylation of Thymine DNA glycosylase (TDG) causes TDG to lose affinity for its product, an abasic site opposite a G residue, and thus increases turnover of the enzyme. During repair of double-strand breaks SUMO1, SUMO2, SUMO3, and the SUMO E3 ligases PIAS1 and PIAS4 accumulate at double-strand breaks where BRCA1, HERC1, RNF168, MDC1, and TP53BP1 are SUMOylated. SUMOylation of BRCA1 may increase its ubiquitin ligase activity while SUMOylation of MDC1 and HERC2 appears to play a role in recruitment of proteins such as RNF4 and RNF8 to double strand breaks. Similarly SUMOylation of RPA1 (RPA70) recruits RAD51 in the homologous recombination pathway.

References

Jackson SP & Durocher D (2013). Regulation of DNA damage responses by ubiquitin and SUMO. Mol. Cell, 49, 795-807.

Ulrich HD (2012). Ubiquitin and SUMO in DNA repair at a glance. J. Cell. Sci., 125, 249-54.

Bekker-Jensen S & Mailand N (2011). The ubiquitin- and SUMO-dependent signaling response to DNA double-strand breaks. FEBS Lett., 585, 2914-9.

Dou H, Huang C, Van Nguyen T, Lu LS & Yeh ET (2011). SUMOylation and de-SUMOylation in response to DNA damage. FEBS Lett., 585, 2891-6.

Flotho A & Melchior F (2013). Sumoylation: a regulatory protein modification in health and disease. Annu. Rev. Biochem., 82, 357-85.

Edit history

Date	Action	Author
2013-02-06	Edited	May B
2013-02-06	Authored	May B
2013-02-10	Created	May B
2015-02-21	Reviewed	Ferrari S
2020-11-20	Modified	Shorser S

Entities found in this pathway (2)

Input	UniProt Id	Input	UniProt Id
POM121C	A8CG34, Q96HA1	STAG1	Q8WVM7

3. Regulation of PTEN mRNA translation (R-HSA-8943723)

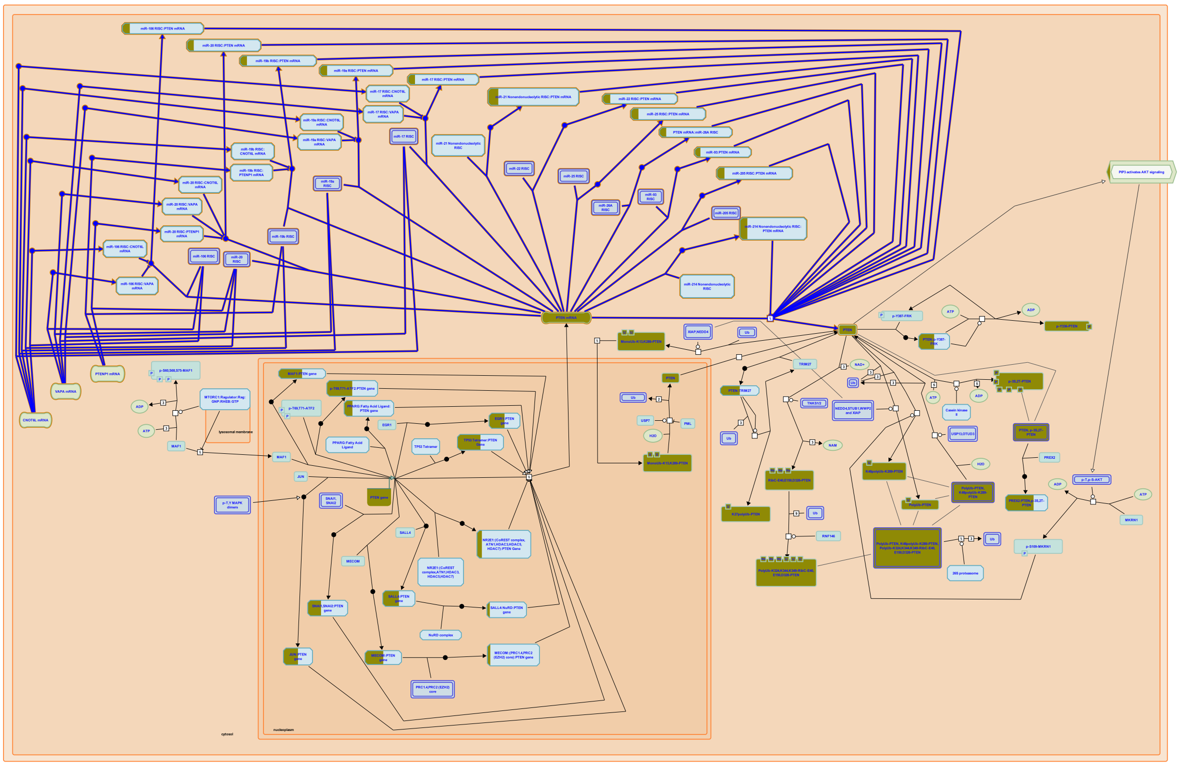

MicroRNAs miR-17, miR-19a, miR-19b, miR-20a, miR-20b, miR-21, miR-22, miR-25, miR 26A1, miR 26A2, miR-93, miR-106a, miR-106b, miR 205, and miR 214 and bind PTEN mRNA and inhibit its translation into protein. These microRNAs are altered in cancer and can account for changes in PTEN levels. There is evidence that PTEN mRNA translation is also inhibited by other microRNAs, such as miR-302 and miR-26B, and these microRNAs will be annotated when additional experimental details become available (Meng et al. 2007, Xiao et al. 2008, Yang et al. 2008, Huse et al. 2009, Kim et al. 2010, Poliseno, Salmena, Riccardi et al. 2010, Zhang et al. 2010, Tay et al. 2011, Qu et al. 2012, Cai et al. 2013). In addition, coding and non coding RNAs can prevent microRNAs from binding to PTEN mRNA. These RNAs are termed competing endogenous RNAs or ceRNAs. Transcripts of the pseudogene PTENP1 and mRNAs transcribed from SERINC1, VAPA and CNOT6L genes exhibit this activity (Poliseno, Salmena, Zhang et al. 2010, Tay et al. 2011, Tay et al. 2014).

References

Meng F, Henson R, Wehbe-Janek H, Ghoshal K, Jacob ST & Patel T (2007). MicroRNA-21 regulates expression of the PTEN tumor suppressor gene in human hepatocellular cancer. Gastroenterology, 133, 647-58.

Xiao C, Srinivasan L, Calado DP, Patterson HC, Zhang B, Wang J, ... Rajewsky K (2008). Lymphoproliferative disease and autoimmunity in mice with increased miR-17-92 expression in lymphocytes. Nat. Immunol., 9, 405-14.

Yang H, Kong W, He L, Zhao JJ, O'Donnell JD, Wang J, ... Cheng JQ (2008). MicroRNA expression profiling in human ovarian cancer: miR-214 induces cell survival and cisplatin resistance by targeting PTEN. Cancer Res., 68, 425-33.

Huse JT, Brennan C, Hambardzumyan D, Wee B, Pena J, Rouhanifard SH, ... Holland EC (2009). The PTEN-regulating microRNA miR-26a is amplified in high-grade glioma and facilitates gliomagenesis in vivo. Genes Dev., 23, 1327-37.

Kim H, Huang W, Jiang X, Pennicooke B, Park PJ & Johnson MD (2010). Integrative genome analysis reveals an oncomir/oncogene cluster regulating glioblastoma survivorship. Proc. Natl. Acad. Sci. U.S.A., 107, 2183-8.

Edit history

Date	Action	Author
2016-08-11	Authored	Salmena L, Carracedo A
2016-09-30	Reviewed	Kriplani N, Leslie N
2016-10-28	Authored	Orlic-Milacic M
2016-10-28	Created	Orlic-Milacic M
2017-05-09	Edited	Orlic-Milacic M
2017-05-12	Modified	Orlic-Milacic M

Entities found in this pathway (1)

Input	UniProt Id
PTEN	P60484

Input	Ensembl Id
PTEN	ENST00000371953

4. Disassembly of the destruction complex and recruitment of AXIN to the membrane (R-HSA-4641262)

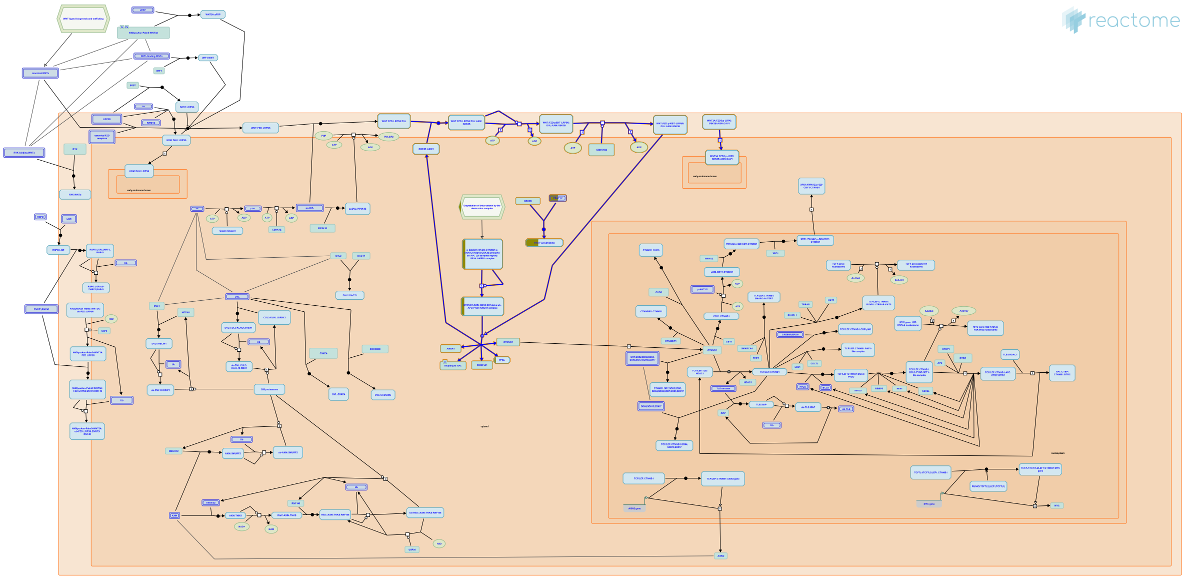

Upon stimulation with WNT ligand, AXIN and GSK3beta are recruited to the plasma membrane through interaction with DVL (Tamai et al, 2004; Mao et al, 2001; reviewed in He et al, 2004). Polymerization of membrane-associated DVL and GSK3beta- and CSNK1-mediated phosphorylation of LRP5/6 establish a feed-forward mechanism for enhanced membrane recruitment of AXIN upon WNT signaling (Tamai et al, 2004; Cong et al, 2004; Zeng et al, 2005; Bilic et al, 2007). In Xenopus oocytes, but not necessarily all sytems, AXIN is present in limiting concentrations and is considered rate limiting for the assembly of the destruction complex (Lee et al, 2003; Benchabane et al, 2008; Tan et al, 2012; reviewed in MacDonald et al, 2009). The recruitment of AXIN away from the destruction complex upon WNT stimulation effectively destabilizes the destruction complex and contributes to the accumulation of free beta-catenin (Kikuchi, 1999; Lee et al, 2003). AXIN association with the destruction complex is also regulated by phosphorylation. In the active destruction complex, AXIN is phosphorylated by GSK3beta; dephosphorylation by protein phosphatase 1 (PP1) or protein phosphatase 2A (PP2A) destabilizes the interaction of AXIN with the other components of the destruction complex and promotes its disassembly (Luo et al, 2007; Willert et al, 1999; Jho et al, 1999). Free AXIN is also subject to degradation by the 26S proteasome in a manner that depends on the poly-ADP-ribosylating enzymes tankyrase 1 and 2 (Huang et al, 2009).

References

Zeng X, Tamai K, Doble B, Li S, Huang H, Habas R, ... He X (2005). A dual-kinase mechanism for Wnt co-receptor phosphorylation and activation. Nature, 438, 873-7.

Tamai K, Zeng X, Liu C, Zhang X, Harada Y, Chang Z & He X (2004). A mechanism for Wnt coreceptor activation. Mol Cell, 13, 149-56.

Mao J, Wang J, Liu B, Pan W, Farr GH 3rd, Flynn C, ... Wu D (2001). Low-density lipoprotein receptor-related protein-5 binds to Axin and regulates the canonical Wnt signaling pathway. Mol Cell, 7, 801-9.

He X, Semenov M, Tamai K & Zeng X (2004). LDL receptor-related proteins 5 and 6 in Wnt/beta-catenin signaling: arrows point the way. Development, 131, 1663-77.

Willert K, Shibamoto S & Nusse R (1999). Wnt-induced dephosphorylation of axin releases beta-catenin from the axin complex. Genes Dev, 13, 1768-73.

Edit history

Date	Action	Author
2007-09-04	Edited	Matthews L
2013-06-25	Authored	Rothfels K
2013-09-28	Created	Rothfels K
2013-10-03	Edited	Gillespie ME
2014-01-22	Reviewed	Rajakulendran N
2014-02-15	Reviewed	van Amerongen R
2014-04-22	Reviewed	Kikuchi A
2020-11-24	Modified	Shorser S

Entities found in this pathway (2)

Input	UniProt Id	Input	UniProt Id
FRAT2	O75474	PPP2R5E	Q16537

5. Defective TPR may confer susceptibility towards thyroid papillary carcinoma (TPC) (R-HSA-5619107)

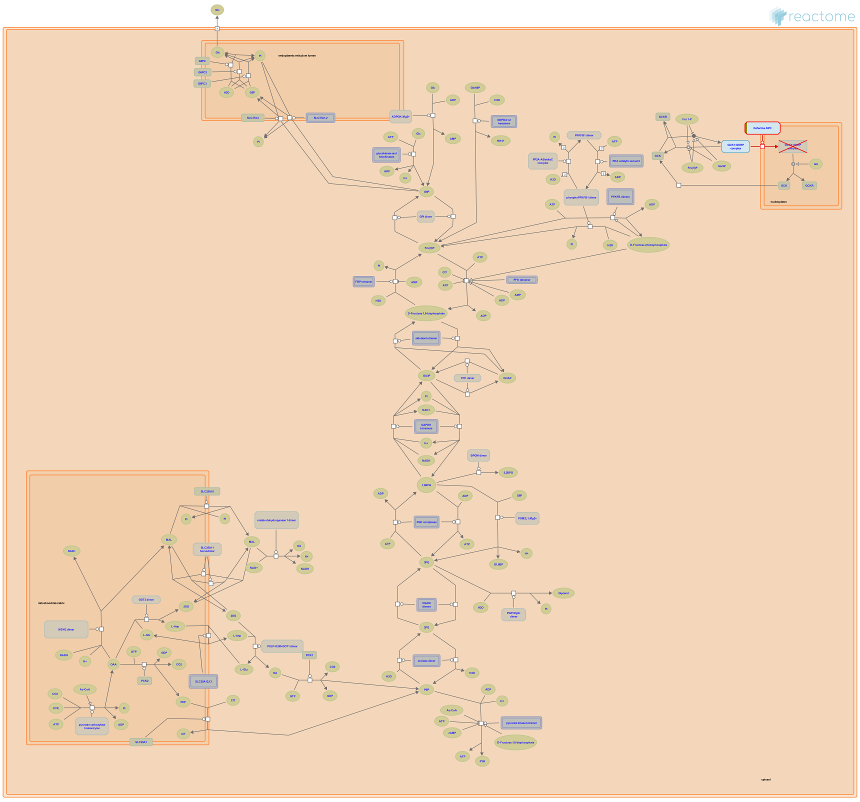

Diseases: papillary carcinoma.

The nuclear pore complex (NPC) trafficks cargo across the nuclear membrane. Nucleoprotein TPR functions as a scaffolding element in the nuclear phase of the NPC essential for normal nucleocytoplasmic transport of proteins and mRNAs. The complex glucokinase (GCK1) and glucokinase regulatory protein (GKRP) can be translocated to the nucleus via the NPC. Defects in TPR may confer susceptibility towards thyroid papillary carcinona (TPC; MIM:18850), a common tumor of the thyroid that typically arises as an irregular, solid or cystic mass from otherwise normal thyroid tissue (Vriens et al. 2009, Bonora et al. 2010).

References

Vriens MR, Suh I, Moses W & Kebebew E (2009). Clinical features and genetic predisposition to hereditary nonmedullary thyroid cancer. Thyroid, 19, 1343-9.

Bonora E, Tallini G & Romeo G (2010). Genetic Predisposition to Familial Nonmedullary Thyroid Cancer: An Update of Molecular Findings and State-of-the-Art Studies. J Oncol, 2010, 385206.

Edit history

Date	Action	Author
2014-08-22	Edited	Jassal B
2014-08-22	Authored	Jassal B
2014-08-22	Created	Jassal B
2015-08-04	Reviewed	Broer S

Date	Action	Author
2017-05-17	Modified	Jassal B

Entities found in this pathway (1)

Input	UniProt Id
POM121C	A8CG34, Q96HA1

6. Transport of the SLBP independent Mature mRNA (R-HSA-159227)

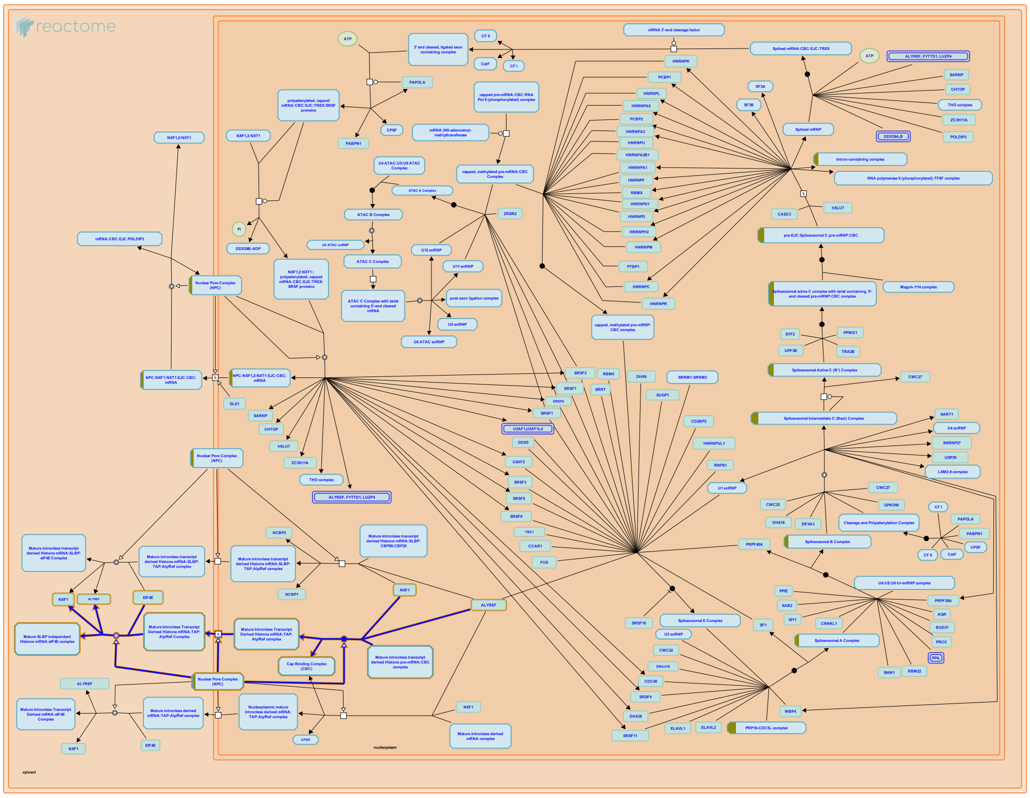

Cellular compartments: nucleoplasm, nuclear envelope, cytosol.

Transport of the SLBP independent Mature mRNA through the nuclear pore.

References

Edit history

Date	Action	Author
2005-02-27	Created	Gillespie ME
2020-11-20	Modified	Shorser S

Entities found in this pathway (1)

Input	UniProt Id
POM121C	A8CG34, Q96HA1

7. Defective B3GALTL causes Peters-plus syndrome (PpS) (R-HSA-5083635)

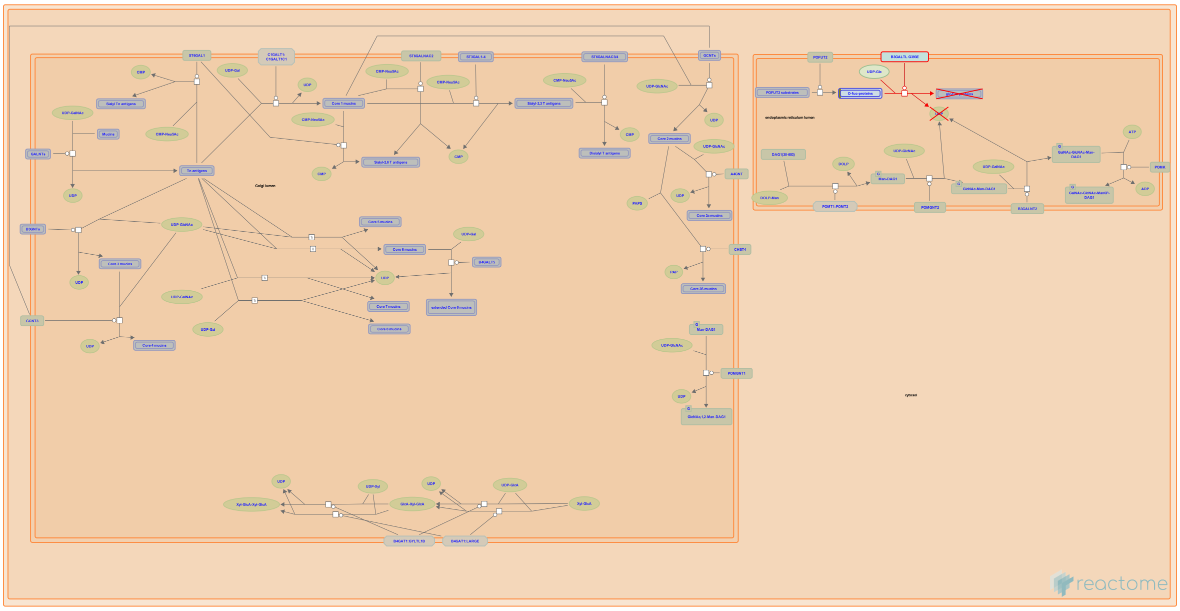

Diseases: eye disease, orofacial cleft.

Human beta-1,3-glucosyltransferase like protein (B3GALTL, HGNC Approved Gene Symbol: B3GLCT; MIM:610308; CAZy family GT31), localised on the ER membrane, glucosylates O-fucosylated proteins. The resultant glc-beta-1,3-fuc disaccharide modification on thrombospondin type 1 repeat (TSR1) domain-containing proteins is thought to assist in the secretion of many of these proteins from the ER lumen, and mediate an ER quality-control mechanism of folded TSRs (Vasudevan et al. 2015). Defects in B3GALTL can cause Peters plus syndrome (PpS; MIM:261540), an autosomal recessive disorder characterised by anterior eye chamber defects, short stature, delay in growth and mental developmental and cleft lip and/or palate (Heinonen & Maki 2009).

References

Heinonen TY & Maki M (2009). Peters'-plus syndrome is a congenital disorder of glycosylation caused by a defect in the beta1,3-glucosyltransferase that modifies thrombospondin type 1 repeats. Ann. Med., 41, 2-10.

Vasudevan D, Takeuchi H, Johar SS, Majerus E & Haltiwanger RS (2015). Peters plus syndrome mutations disrupt a noncanonical ER quality-control mechanism. Curr. Biol., 25, 286-95.

Edit history

Date	Action	Author
2013-11-07	Edited	Jassal B
2013-11-07	Authored	Jassal B
2013-11-07	Created	Jassal B
2015-12-18	Modified	Jassal B
2015-12-18	Reviewed	Hansen L, Joshi HJ

Entities found in this pathway (2)

Input	UniProt Id	Input	UniProt Id
ADAMTS18	Q8TE60	ADAMTS5	Q9UNA0

8. SUMOylation of SUMOylation proteins (R-HSA-4085377)

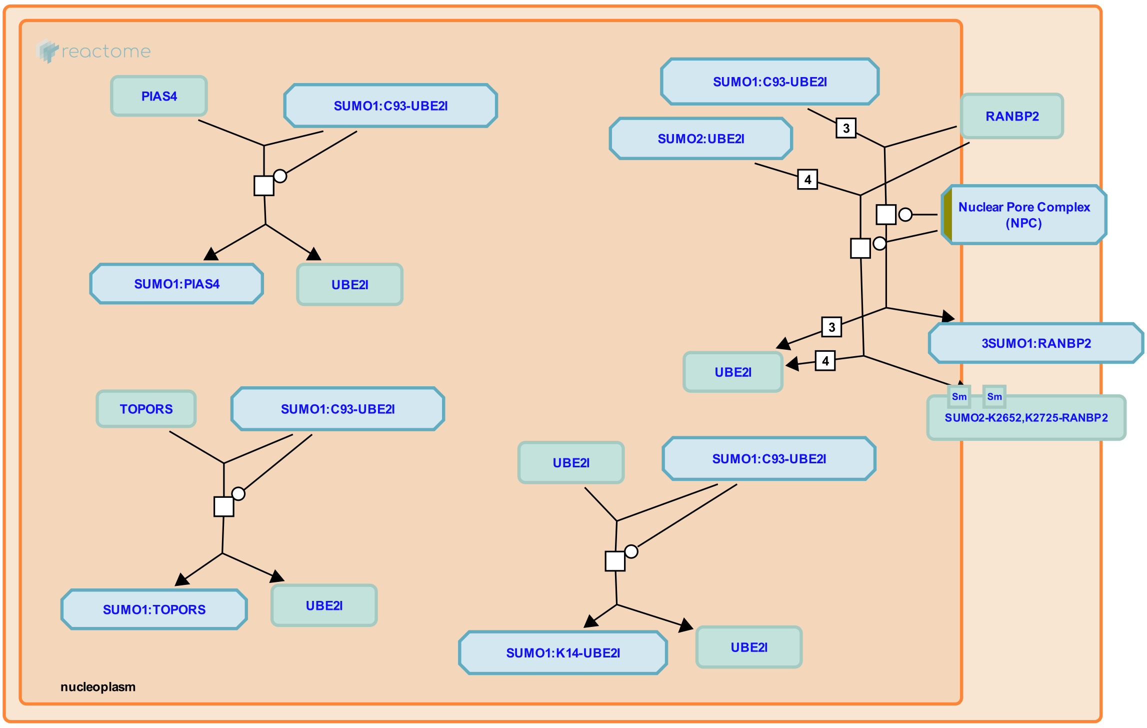

Cellular compartments: nucleoplasm, nuclear envelope.

SUMOylation processes themselves can be controlled by SUMOylation (reviewed in Wilkinson and Henley 2010). The SUMO E3 ligases PIAS4, RANBP2, and TOPORS are SUMOylated, as is the single SUMO E2 enzyme, UBE2I (UBC9). SUMOylation affects the subcellular location of PIAS4 and TOPORS and affects the activity of PIAS4 and UBE2I.

References

Wilkinson KA & Henley JM (2010). Mechanisms, regulation and consequences of protein SUMOylation. Biochem. J., 428, 133-45.

Edit history

Date	Action	Author
2013-08-03	Edited	May B
2013-08-03	Authored	May B
2013-08-05	Created	May B
2018-05-09	Reviewed	Niskanen E
2018-08-08	Reviewed	Niskanen E
2020-11-20	Modified	Shorser S

Entities found in this pathway (1)

Input	UniProt Id
POM121C	A8CG34, Q96HA1

9. PTEN Loss of Function in Cancer (R-HSA-5674404)

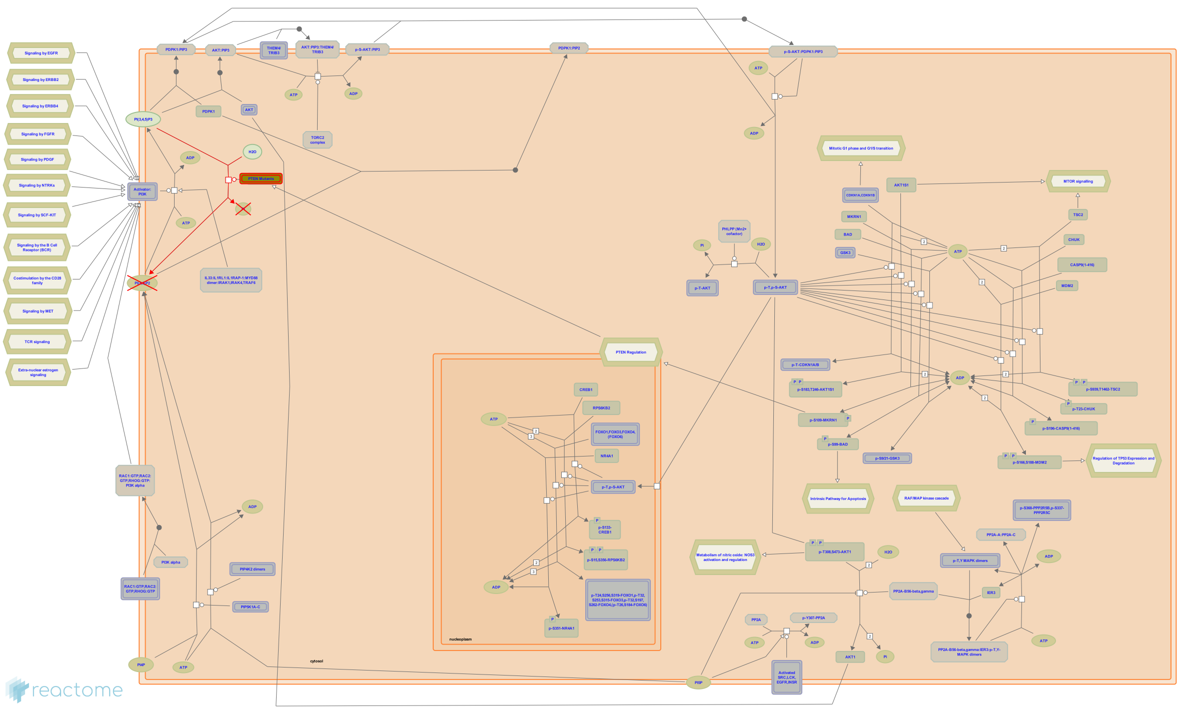

Diseases: cancer.

Loss-of-function mutations affecting the phosphatase domain of PTEN are frequently found in sporadic cancers (Kong et al. 1997, Lee et al. 1999, Han et al. 2000), as well as in PTEN hamartoma tumor syndromes (PHTS) (Marsh et al. 1998). PTEN can also be inactivated by gene deletion or epigenetic silencing, or indirectly by overexpression of microRNAs that target PTEN mRNA (Huse et al. 2009). Cells with deficient PTEN function have increased levels of PIP3, and therefore increased AKT activity. For a recent review, please refer to Hollander et al. 2011.

References

Kong D, Suzuki A, Zou TT, Sakurada A, Kemp LW, Wakatsuki S, ... Horii A (1997). PTEN1 is frequently mutated in primary endometrial carcinomas. Nat. Genet., 17, 143-4.

Lee JO, Yang H, Georgescu MM, Di Cristofano A, Maehama T, Shi Y, ... Pavletich NP (1999). Crystal structure of the PTEN tumor suppressor: implications for its phosphoinositide phosphatase activity and membrane association. Cell, 99, 323-34.

Han SY, Kato H, Kato S, Suzuki T, Shibata H, Ishii S, ... Ishioka C (2000). Functional evaluation of PTEN missense mutations using in vitro phosphoinositide phosphatase assay. Cancer Res, 60, 3147-51.

Marsh DJ, Coulon V, Lunetta KL, Rocca-Serra P, Dahia PL, Zheng Z, ... Eng C (1998). Mutation spectrum and genotype-phenotype analyses in Cowden disease and Bannayan-Zonana syndrome, two hamartoma syndromes with germline PTEN mutation. Hum. Mol. Genet., 7, 507-15.

Huse JT, Brennan C, Hambardzumyan D, Wee B, Pena J, Rouhanifard SH, ... Holland EC (2009). The PTEN-regulating microRNA miR-26a is amplified in high-grade glioma and facilitates gliomagenesis in vivo. Genes Dev., 23, 1327-37.

Edit history

Date	Action	Author
2012-07-18	Authored	Orlic-Milacic M
2012-08-13	Reviewed	Yuzugullu H, Thorpe L, Zhao JJ
2015-02-12	Created	Orlic-Milacic M
2020-11-24	Modified	Shorser S

Entities found in this pathway (1)

Input	UniProt Id
PTEN	P60484

10. PIP3 activates AKT signaling (R-HSA-1257604)

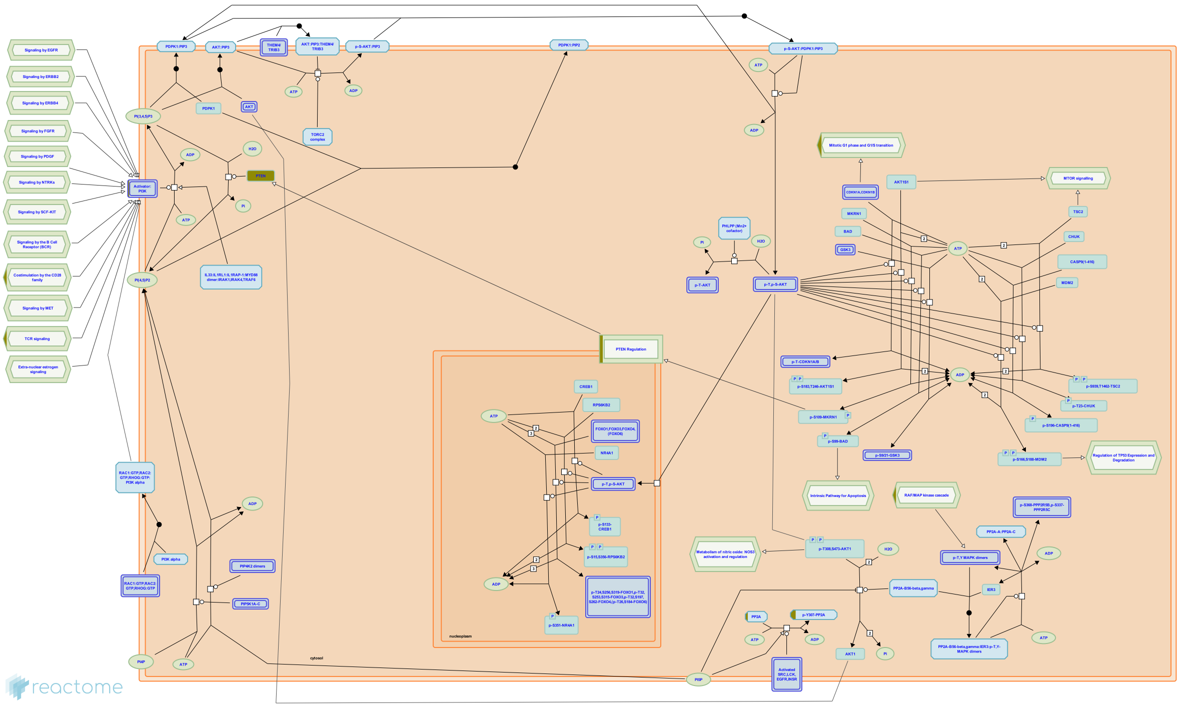

Signaling by AKT is one of the key outcomes of receptor tyrosine kinase (RTK) activation. AKT is activated by the cellular second messenger PIP3, a phospholipid that is generated by PI3K. In ustimulated cells, PI3K class IA enzymes reside in the cytosol as inactive heterodimers composed of p85 regulatory subunit and p110 catalytic subunit. In this complex, p85 stabilizes p110 while inhibiting its catalytic activity. Upon binding of extracellular ligands to RTKs, receptors dimerize and undergo autophosphorylation. The regulatory subunit of PI3K, p85, is recruited to phosphorylated cytosolic RTK domains either directly or indirectly, through adaptor proteins, leading to a conformational change in the PI3K IA heterodimer that relieves inhibition of the p110 catalytic subunit. Activated PI3K IA phosphorylates PIP2, converting it to PIP3; this reaction is negatively regulated by PTEN phosphatase. PIP3 recruits AKT to the plasma membrane, allowing TORC2 to phosphorylate a conserved serine residue of AKT. Phosphorylation of this serine induces a conformation change in AKT, exposing a conserved threonine residue that is then phosphorylated by PDPK1 (PDK1). Phosphorylation of both the threonine and the serine residue is required to fully activate AKT. The active AKT then dissociates from PIP3 and phosphorylates a number of cytosolic and nuclear proteins that play important roles in cell survival and metabolism. For a recent review of AKT signaling, please refer to Manning and Cantley, 2007.

References

Edit history

Date	Action	Author
2007-11-08	Reviewed	Greene LA
2011-05-02	Created	Orlic-Milacic M
2012-06-21	Revised	Orlic-Milacic M
2012-08-13	Reviewed	Yuzugullu H, Thorpe L, Zhao JJ
2020-11-20	Modified	Shorser S

Entities found in this pathway (3)

Input	UniProt Id	Input	UniProt Id	Input	UniProt Id
ICOS	Q9Y6W8-1	PPP2R5E	Q16537	PTEN	P60484

Input	Ensembl Id
PTEN	ENSG00000171862, ENST00000371953

11. Transport of the SLBP Dependant Mature mRNA (R-HSA-159230)

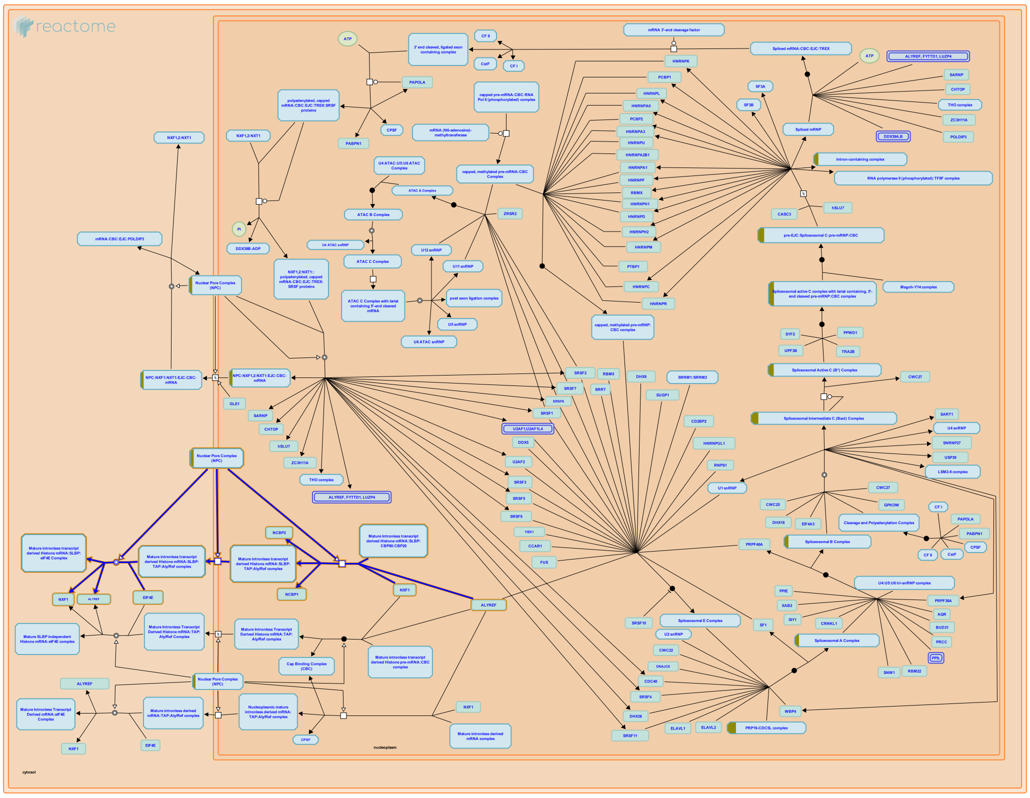

Cellular compartments: nucleoplasm, nuclear envelope, cytosol.

Transport of U7 snRNP and stem-loop binding protein (SLBP) processed mRNA.

References

Edit history

Date	Action	Author
2005-02-27	Created	Gillespie ME
2020-11-20	Modified	Shorser S

Entities found in this pathway (1)

Input	UniProt Id
POM121C	A8CG34, Q96HA1

12. Regulation of Glucokinase by Glucokinase Regulatory Protein (R-HSA-170822)

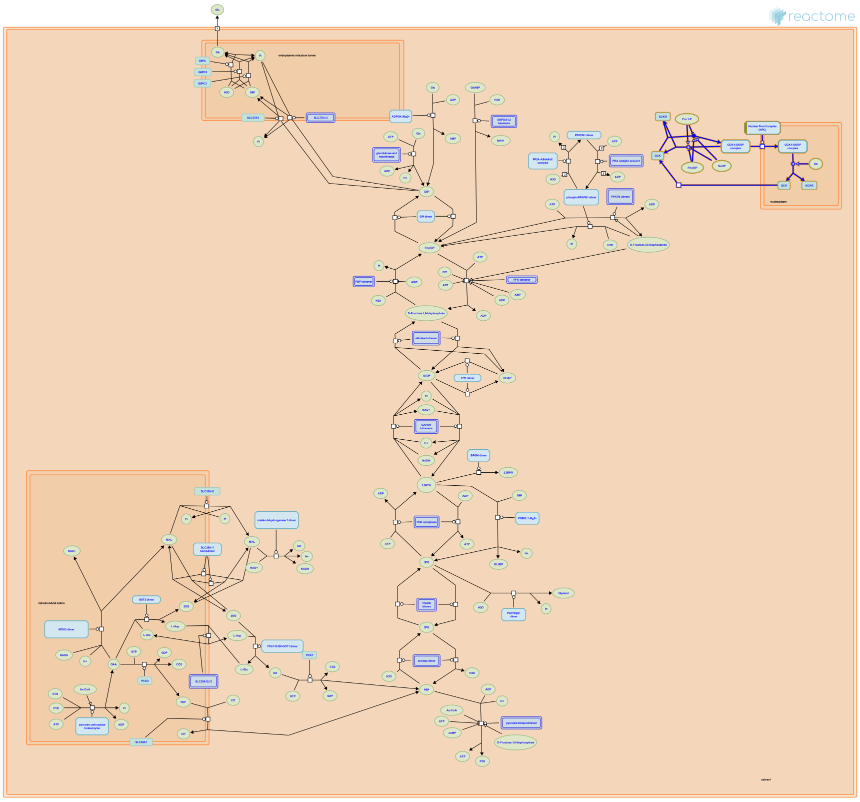

Glucokinase (GCK1) is negatively regulated by glucokinase regulatory protein (GKRP), which reversibly binds the enzyme to form an inactive complex. Binding is stimulated by fructose 6-phosphate and sorbitol 6-phosphate (hence high concentrations of these molecules tend to reduce GCK1 activity) and inhibited by fructose 1-phosphate (hence a high concentration of this molecule tends to increase GCK1 activity). Once formed, the complex is translocated to the nucleus. In the presence of high glucose concentrations, the nuclear GCK1:GKRP complex dissociates, freeing GCK1 to return to the cytosol. The free GKRP is thought also to return to the cytosol under these conditions, but this return has not been confirmed experimentally. Possible physiological roles for this sequestration process are to decrease futile cycling between glucose and glucose 6 phosphate in hepatocytes under low-glucose conditions, and to decrease the lag between a rise in intracellular glucose levels and the onset of glucose phosphorylation in both hepatocytes and pancreatic beta cells (Brocklehurst et al. 2004; Shiota et al. 1999).

References

Brocklehurst KJ, Davies RA & Agius L (2004). Differences in regulatory properties between human and rat glucokinase regulatory protein. Biochem J, 378, 693-7.

Shiota C, Coffey J, Grimsby J, Grippo JF & Magnuson MA (1999). Nuclear import of hepatic glucokinase depends upon glucokinase regulatory protein, whereas export is due to a nuclear export signal sequence in glucokinase. J Biol Chem, 274, 37125-30.

Edit history

Date	Action	Author
2006-01-17	Created	D'Eustachio P

Date	Action	Author
2006-02-20	Edited	D'Eustachio P
2009-12-12	Revised	D'Eustachio P
2020-11-20	Modified	Shorser S

Entities found in this pathway (1)

Input	UniProt Id
POM121C	A8CG34, Q96HA1

13. Regulation of HSF1-mediated heat shock response (R-HSA-3371453)

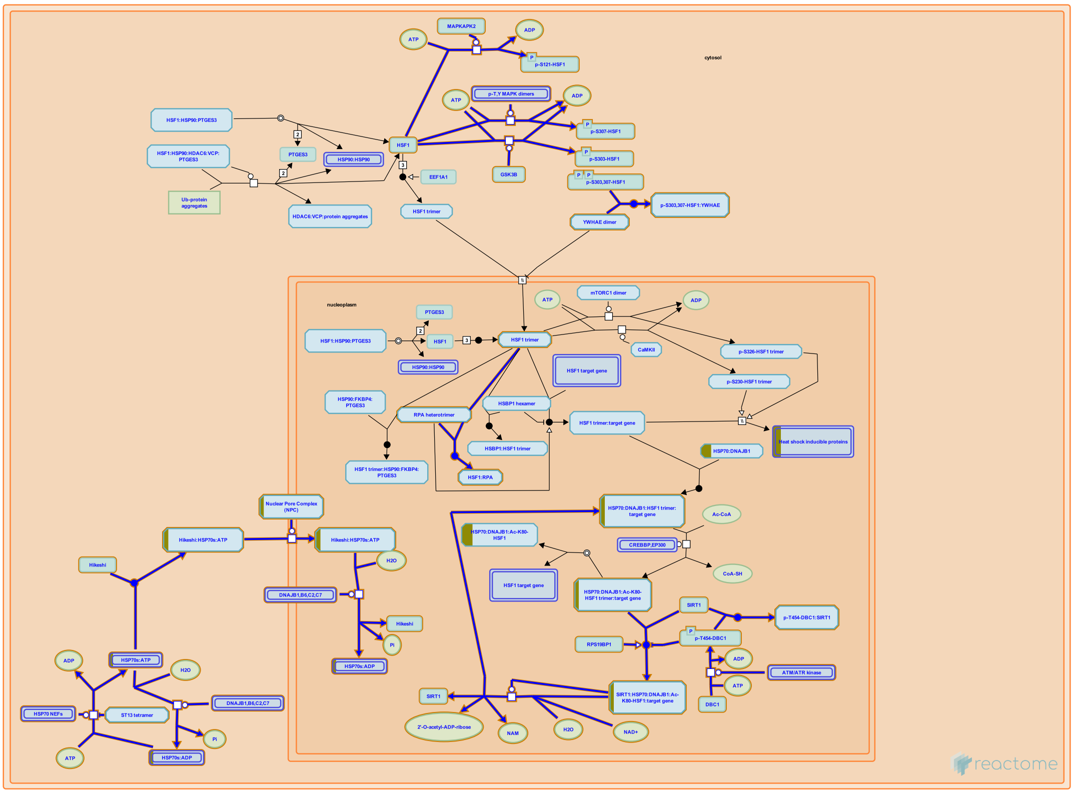

The ability of HSF1 to respond to cellular stresses is under negative regulation by chaperones, modulation of nucleocytoplasmic shuttling, post-translational modifications and transition from monomeric to trimeric state.

References

Zuo J, Rungger D & Voellmy R (1995). Multiple layers of regulation of human heat shock transcription factor 1. Mol. Cell. Biol., 15, 4319-30.

Edit history

Date	Action	Author
2013-05-13	Created	Shamovsky V
2013-10-29	Authored	Shamovsky V
2014-02-17	Edited	Shamovsky V
2014-02-17	Reviewed	Pani B
2020-11-20	Modified	Shorser S

Entities found in this pathway (2)

Input	UniProt Id	Input	UniProt Id
POM121C	A8CG34, Q96HA1	STAG1	P11142

14. O-glycosylation of TSR domain-containing proteins (R-HSA-5173214)

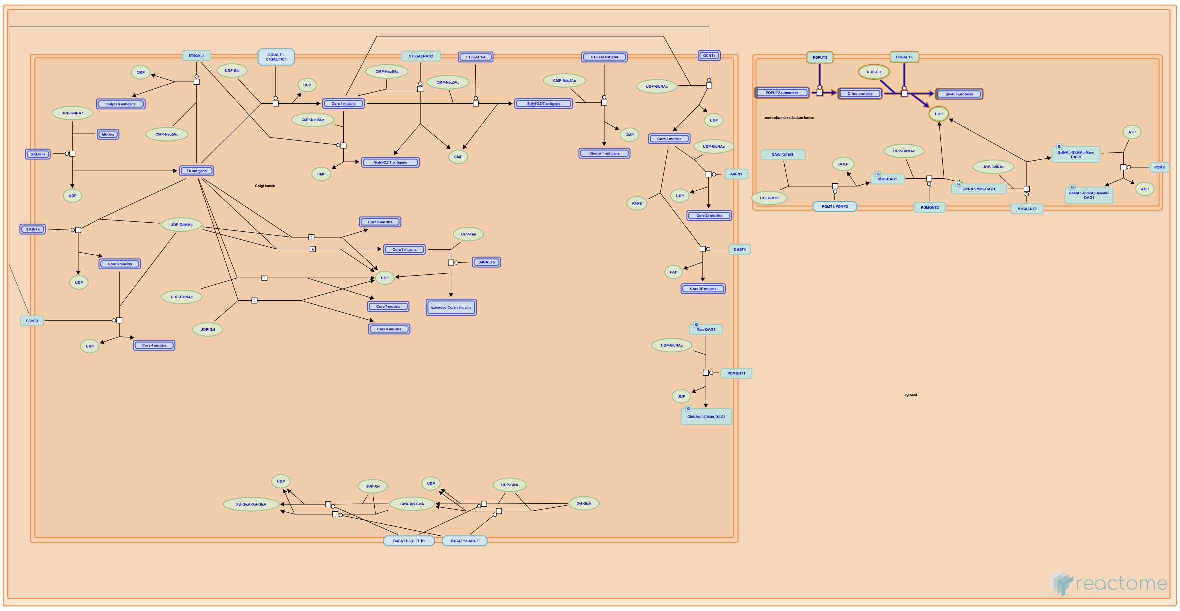

The O-fucosylation of proteins containing thrombospondin type 1 repeat (TSR) domains is an important PTM, regulating many biological processes such as Notch signalling, inflammation, wound healing, angiogenesis amd neoplasia (Adams & Tucker 2000, Moremen et al. 2012). Fucose addition is carried out by two protein fucosyltransferases, POFUT1 and 2. Only POFUT2 recognises the consensus sequence CSXS/TCG found in TSR1 domains and the fucosyl residue is attached to the hydroxyl group of conserved serine (S) or threonine (T) residues within the consensus sequence. The modification was first demonstrated on thrombospondin 1, found in platelets and the ECM (Hofsteenge et al. 2001, Luo et al. 2006). The resulting O-fucosyl-protein is subsequently a substrate for beta-1,3-glucosyltransferase-like protein (B3GALTL), which adds a glucosyl moiety to form the rare disaccharide modification Glc-beta-1,3-Fuc. More than 60 human proteins contain TSR1 domains, The disaccharide modification has been demonstrated on a small number of these TSR1 domain-containing proteins such as thrombospondin 1 (Hofsteenge et al. 2001, Luo et al. 2006), properdin (Gonzalez de Peredo et al. 2002) and F-spondin (Gonzalez de Peredo et al. 2002). The ADAMTS (a disintegrin-like and metalloprotease domain with thrombospondin type-1 repeats) superfamily consists of 19 secreted metalloproteases (ADAMTS proteases) and at lease five ADAMTS-like proteins in humans. Five members of the ADAMTS superfamily have also had experimental confirmation of the disaccharide modification. Examples are ADAMTS13 (Ricketts et al. 2007) and ADAMTSL1 (Wang et al. 2007). In the two reactions described here, the TSR1 domain-containing proteins with similarity to the experimentally confirmed ones are included as putative substrates.

References

Adams JC & Tucker RP (2000). The thrombospondin type 1 repeat (TSR) superfamily: diverse proteins with related roles in neuronal development. Dev. Dyn., 218, 280-99.

Moremen KW, Tiemeyer M & Nairn AV (2012). Vertebrate protein glycosylation: diversity, synthesis and function. Nat. Rev. Mol. Cell Biol., 13, 448-62.

Hofsteenge J, Huwiler KG, Macek B, Hess D, Lawler J, Mosher DF & Peter-Katalinic J (2001). C-mannosylation and O-fucosylation of the thrombospondin type 1 module. J. Biol. Chem., 276, 6485-98.

Luo Y, Nita-Lazar A & Haltiwanger RS (2006). Two distinct pathways for O-fucosylation of epidermal growth factor-like or thrombospondin type 1 repeats. J. Biol. Chem., 281, 9385-92.

Ricketts LM, Dlugosz M, Luther KB, Haltiwanger RS & Majerus EM (2007). O-fucosylation is required for ADAMTS13 secretion. J. Biol. Chem., 282, 17014-23.

Edit history

Date	Action	Author
2013-11-25	Edited	Jassal B
2013-11-25	Authored	Jassal B
2013-11-25	Created	Jassal B
2014-02-07	Reviewed	D'Eustachio P
2020-11-20	Modified	Shorser S

Entities found in this pathway (2)

Input	UniProt Id	Input	UniProt Id
ADAMTS18	Q8TE60	ADAMTS5	Q9UNA0

15. Nuclear import of Rev protein (R-HSA-180746)

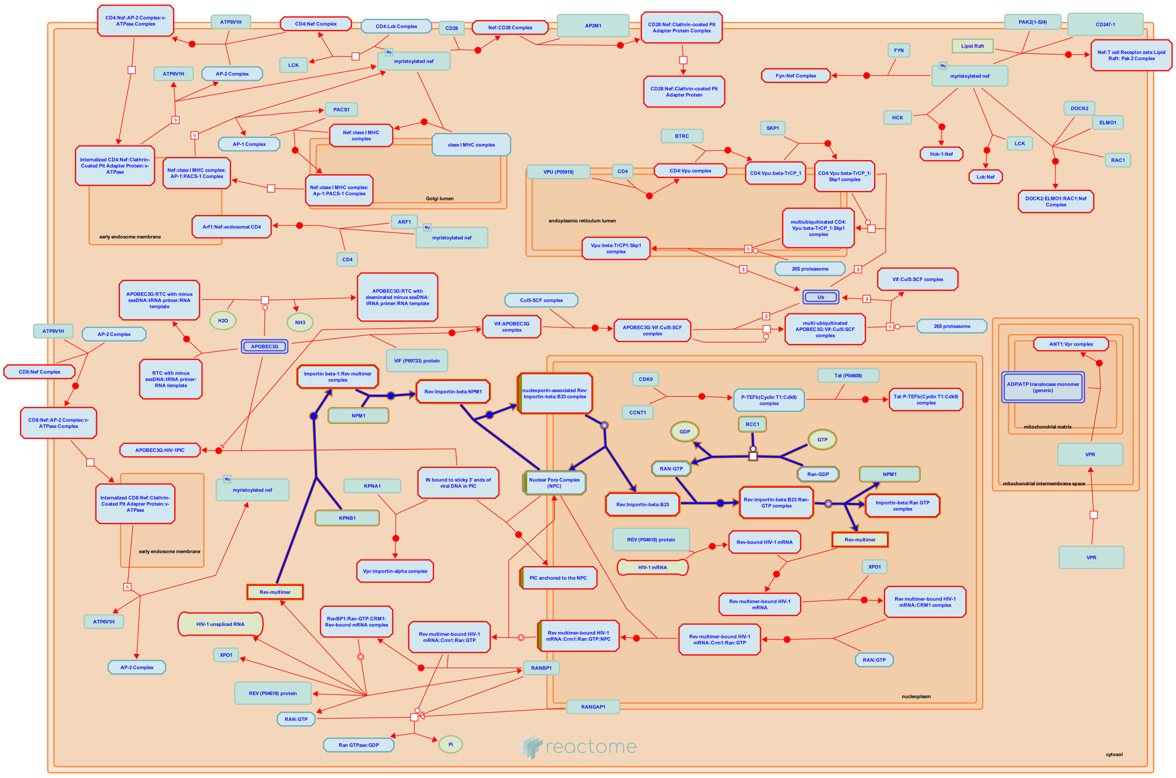

Diseases: Human immunodeficiency virus infectious disease.

Nuclear import of Rev involves the cellular proteins including importin-beta and B23 and is mediated by an arginine-rich nuclear localization signal (NLS) within the RNA binding domain of the Rev protein. The NLS of Rev associates with importin- beta as well as B23 which has been shown to function in the nuclear import of ribosomal proteins. The Rev-importin beta-B23 complex associates with the nuclear pore through interactions between importin beta and nucleoporin. Upon entry into the nucleus, Ran-GTP associates with importin beta resulting in in the disassembly of the importin beta-Rev-B23 complex and the release of Rev cargo.

References

Edit history

Date	Action	Author
2006-06-02	Created	Matthews L
2006-06-08	Authored	Matthews L
2007-02-01	Edited	Matthews L
2007-02-01	Reviewed	Kumar A
2014-12-13	Modified	Gillespie ME

Entities found in this pathway (1)

Input	UniProt Id
POM121C	A8CG34, Q96HA1

16. Nuclear Pore Complex (NPC) Disassembly (R-HSA-3301854)

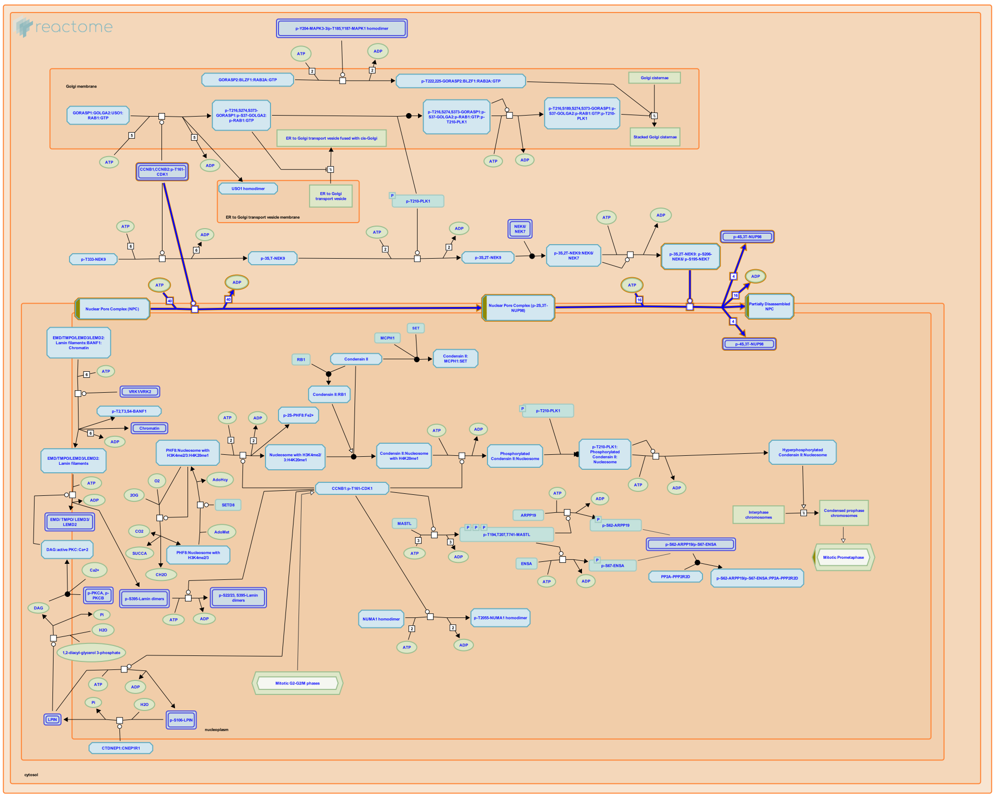

Nuclear envelope breakdown in mitosis involves permeabilization of the nuclear envelope through disassembly of the nuclear pore complex (NPC) (reviewed by Guttinger et al. 2009). Nucleoporin NUP98, located at both the cytoplasmic and the nucleoplasmic side of the NPC (Griffis et al. 2003), and involved in the formation of the transport barrier through its FG (phenylalanine glycine) repeats that protrude into the central cavity of the NPC (Hulsmann et al. 2012), is probably the first nucleoporin that dissociates from the NPC at the start of mitotic NPC disassembly (Dultz et al. 2008). NUP98 dissociation is triggered by phosphorylation. Phosphorylation of NUP98 by CDK1 and NIMA family kinases NEK6 and/or NEK7 is needed for NUP98 dissociation from the NPC (Laurell et al. 2011). While the phosphorylation of NUP98 by CDK1 and NEK6/7 is likely to occur simultaneously, CDK1 and NEK6/7-mediated phosphorylations are shown as separate events, for clarity purposes.

References

Dultz E, Zanin E, Wurzenberger C, Braun M, Rabut G, Sironi L & Ellenberg J (2008). Systematic kinetic analysis of mitotic dis- and reassembly of the nuclear pore in living cells. J. Cell Biol., 180, 857-65.

Griffis ER, Xu S & Powers MA (2003). Nup98 localizes to both nuclear and cytoplasmic sides of the nuclear pore and binds to two distinct nucleoporin subcomplexes. Mol. Biol. Cell, 14, 600-10.

Güttinger S, Laurell E & Kutay U (2009). Orchestrating nuclear envelope disassembly and reassembly during mitosis. Nat. Rev. Mol. Cell Biol., 10, 178-91.

Hülsmann BB, Labokha AA & Görlich D (2012). The permeability of reconstituted nuclear pores provides direct evidence for the selective phase model. Cell, 150, 738-51.

Laurell E, Beck K, Krupina K, Theerthagiri G, Bodenmiller B, Horvath P, ... Kutay U (2011). Phosphorylation of Nup98 by multiple kinases is crucial for NPC disassembly during mitotic entry. Cell, 144, 539-50.

Edit history

Date	Action	Author
2013-01-18	Authored	Orlic-Milacic M
2013-01-23	Edited	Gillespie ME
2013-04-22	Created	Orlic-Milacic M
2013-05-17	Reviewed	Antonin W
2020-11-20	Modified	Shorser S

Entities found in this pathway (1)

Input	UniProt Id
POM121C	A8CG34, Q96HA1

17. Ovarian tumor domain proteases (R-HSA-5689896)

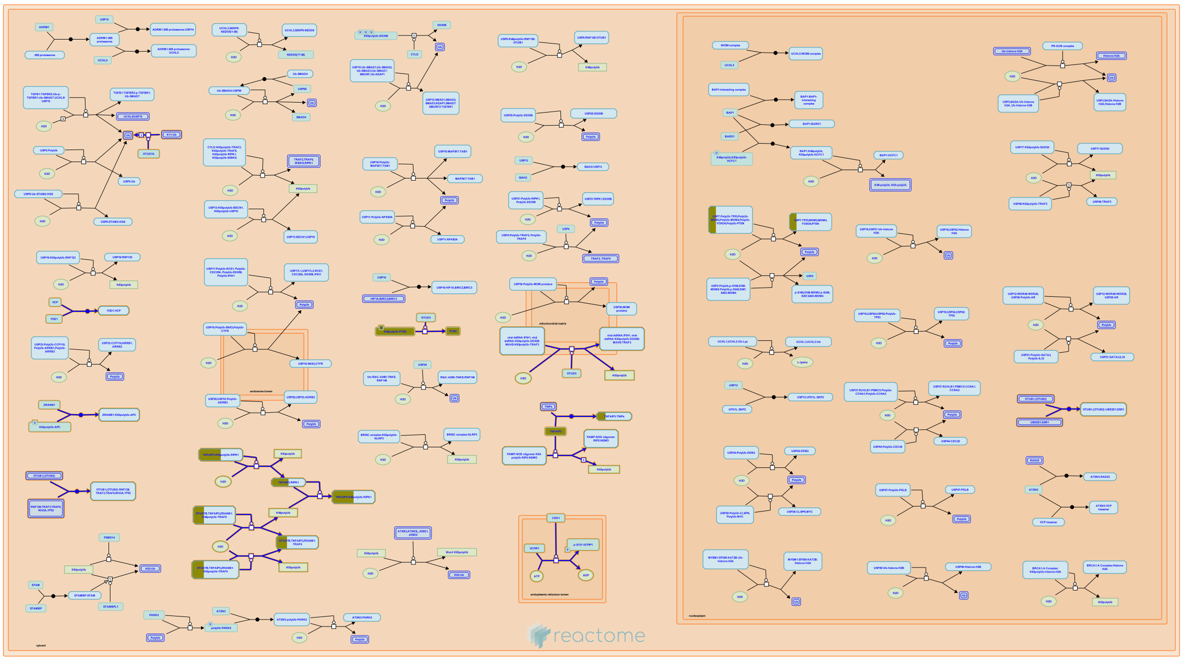

Humans have 16 Ovarian tumour domain (OTU) family DUBs that can be evolutionally divided into three classes, the OTUs, the Otubains (OTUBs), and the A20-like OTUs (Komander et al. 2009).

OTU family DUBs can be highly selective in the type of ubiquitin crosslinks they cleave. OTUB1 is specific for K48-linked chains, whereas OTUB2 can cleave K11, K63 and K48-linked poly-Ub (Wang et al. 2009, Edelmann et al. 2009, Mevissen et al. 2013). A20 prefers K48-linked chains, Cezanne is specific for K11-linked chains, and TRABID acts on both K29, K33 and K63-linked poly-Ub (Licchesi et al. 2011, Komander & Barford 2008, Bremm et al. 2010, Mevissen et al. 2013). The active site of the OTU domain contains an unusual loop not seen in other thiol-DUBs and can lack an obvious catalytic Asp/Asn (Komander & Barford 2009, Messick et al. 2008, Lin et al. 2008). A20 and OTUB1 have an unusual mode of activity, binding directly to E2 enzymes (Nakada et al. 2010, Wertz et al. 2004).

References

Mevissen TE, Hospenthal MK, Geurink PP, Elliott PR, Akutsu M, Arnaudo N, ... Komander D (2013). OTU deubiquitinases reveal mechanisms of linkage specificity and enable ubiquitin chain restriction analysis. Cell, 154, 169-84.

Edit history

Date	Action	Author
2015-04-16	Authored	Jupe S
2015-04-24	Created	Jupe S
2016-05-05	Edited	Jupe S
2016-05-16	Reviewed	Meldal BH
2020-11-20	Modified	Shorser S

Entities found in this pathway (2)

Input	UniProt Id	Input	UniProt Id
PTEN	P60484	TNFAIP3	P21580

18. SUMOylation of ubiquitinylation proteins (R-HSA-3232142)

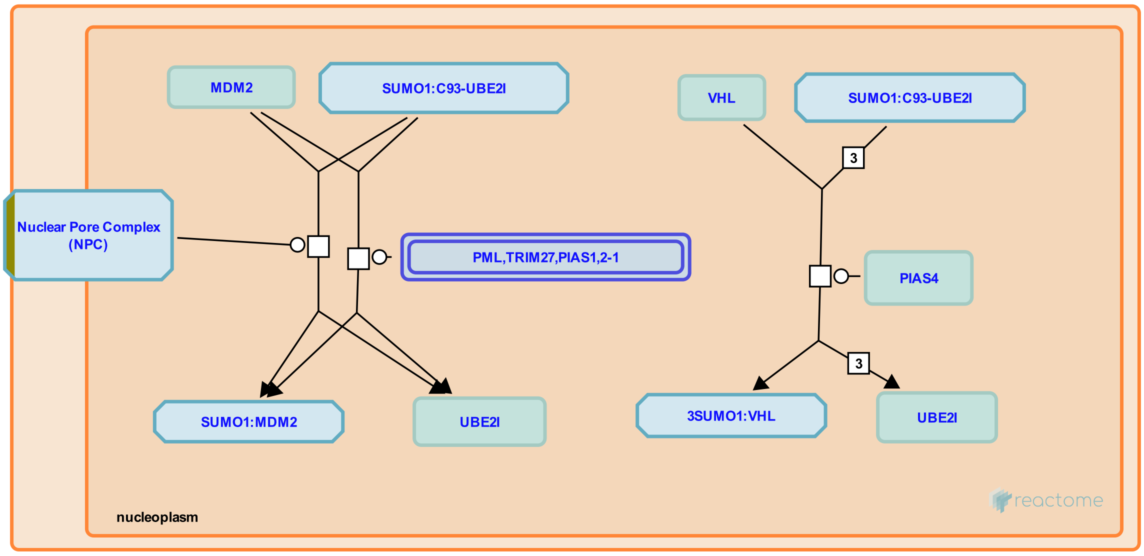

Cellular compartments: nucleoplasm.

Several ubiquitin E3 ligases are regulated by SUMOylation (reviewed in Wilson and Heaton 2008). SUMOylation appears to be necessary for nuclear import of MDM2, the E3 ligase that ubiquitinylates TP53 (p53). SUMOylation of VHL abolishes its ubiquitin ligase activity. HERC2, RNF168, and BRCA1 are ubiquitin ligases that are SUMOylated during DNA damage response and repair.

References

Wilson VG & Heaton PR (2008). Ubiquitin proteolytic system: focus on SUMO. Expert Rev Proteomics, 5, 121-35.

Edit history

Date	Action	Author
2013-03-23	Edited	May B
2013-03-23	Authored	May B
2013-03-27	Created	May B
2018-05-09	Reviewed	Niskanen E
2018-08-08	Reviewed	Niskanen E
2020-11-20	Modified	Shorser S

Entities found in this pathway (1)

Input	UniProt Id
POM121C	A8CG34, Q96HA1

19. Vpr-mediated nuclear import of PICs (R-HSA-180910)

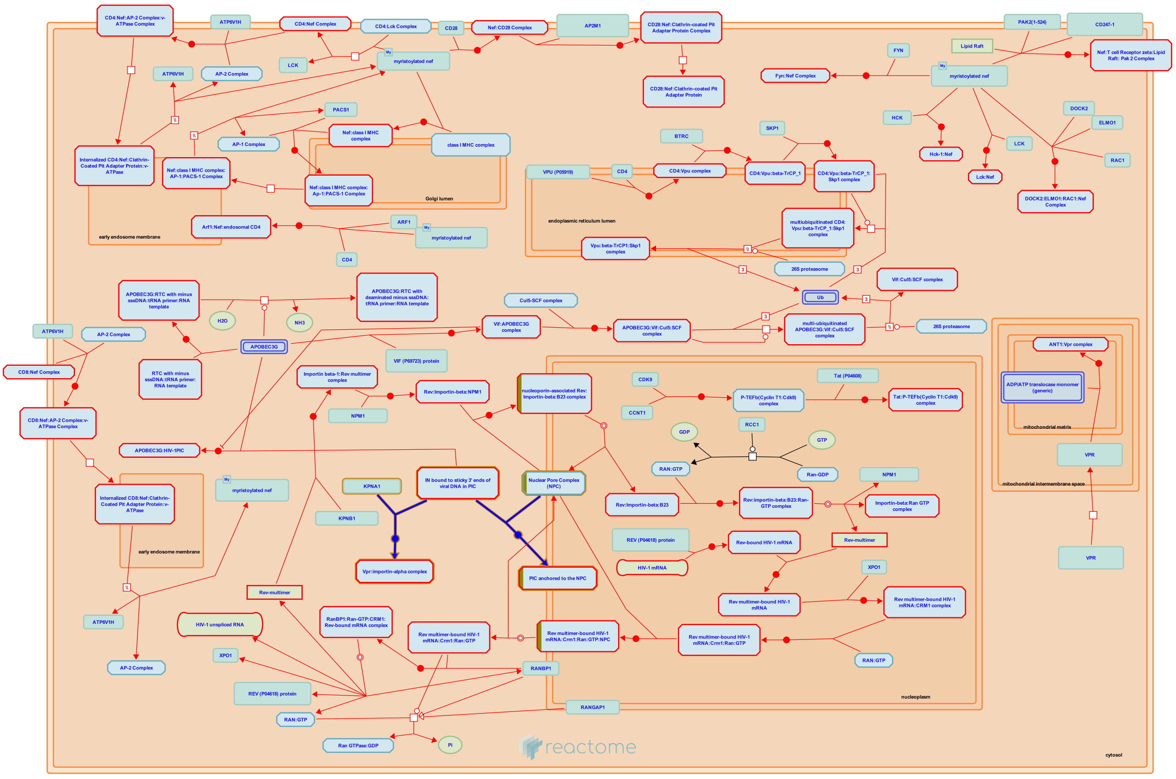

Diseases: Human immunodeficiency virus infectious disease.

Vpr appears to function in anchoring the PIC to the nuclear envelope. This anchoring likely involves interactions between Vpr and host nucleoporins.

References

Edit history

Date	Action	Author
2006-05-15	Authored	Matthews L
2006-06-08	Edited	Matthews L
2006-06-08	Created	Matthews L
2006-07-12	Reviewed	Zhao RY
2016-11-08	Modified	Shorser S

Entities found in this pathway (1)

Input	UniProt Id
POM121C	A8CG34, Q96HA1

20. Rev-mediated nuclear export of HIV RNA (R-HSA-165054)

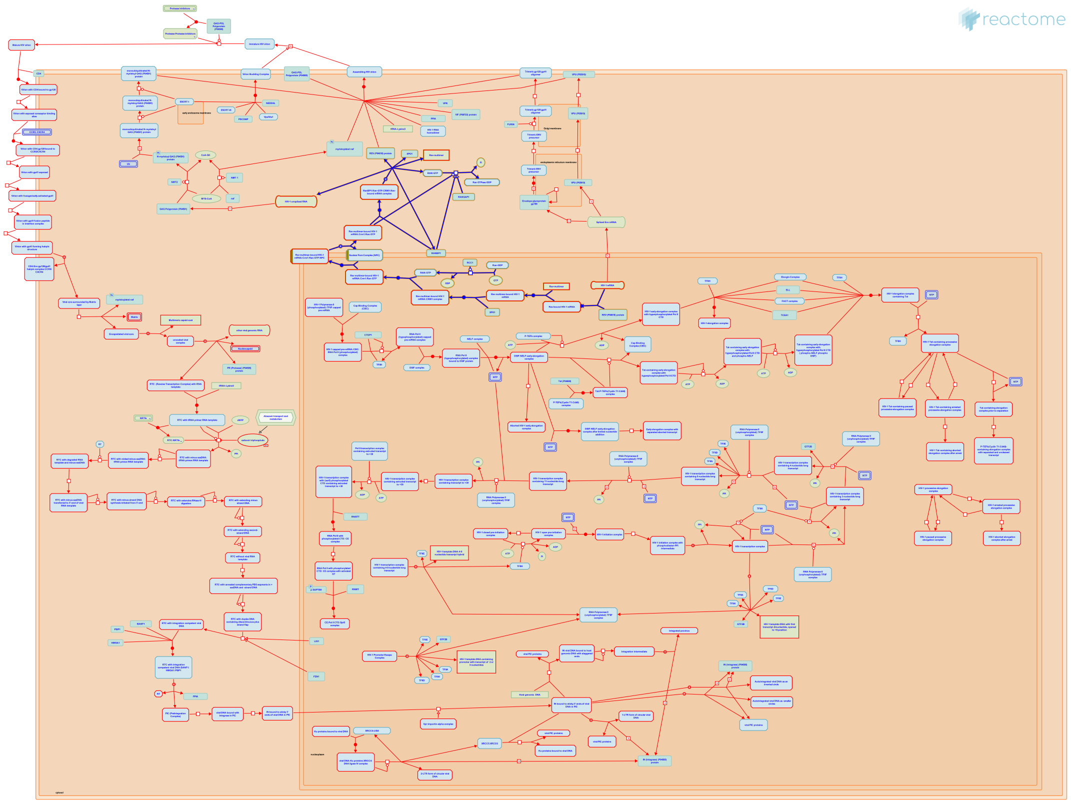

Diseases: Human immunodeficiency virus infectious disease.

The HIV-1 genome contains 9 genes encoded by a single transcript. In order for the virus to replicate, unspliced, singly-spliced and fully spliced viral mRNA must be exported from the nucleus. The HIV-1 mRNA splice sites are inefficient resulting it the accumulation of a pool of incompletely spliced RNAs (Staffa and Cochrane, 1994). In the early stages of the viral life cycle, or in the absence of the viral Rev protein, completely spliced viral mRNA which encode the regulatory proteins Tat, Nef and Rev are exported from the nucleus while the incompletely spliced structural protein encoding transcripts are held within the nucleus by cellular proteins that normally function in preventing the nuclear export of cellular pre-mRNA. Export of both unspliced and partially spliced mRNA is mediated by the viral protein Rev which is recruited, along with cellular cofactors, to the Rev Response Element (RRE) within the HIV-1 mRNA sequence (Malim et al., 1990; Fischer et al., 1994). The cellular hRIP protein is essential for correct Rev-mediated export of viral RNAs to the cytoplasm (Sanchez-Velar et al., 2004; Yu et al., 2005).

References

Fritz CC & Green MR (1996). HIV Rev uses a conserved cellular protein export pathway for the nucleocytoplasmic transport of viral RNAs. Curr Biol, 6, 848-54.

Cullen BR (1998). Retroviruses as model systems for the study of nuclear RNA export. Virology, 249, 203-10.

Cullen BR (2003). Nuclear mRNA export: insights from virology. Trends Biochem Sci, 28, 419-24.

Edit history

Date	Action	Author
2005-07-27	Authored	Rice AP, Matthews L
2005-07-27	Created	Matthews L
2006-06-08	Edited	Matthews L
2007-02-01	Reviewed	Kumar A
2020-11-24	Modified	Shorser S

Entities found in this pathway (1)

Input	UniProt Id
POM121C	A8CG34, Q96HA1

21. Transport of Mature mRNA Derived from an Intronless Transcript (R-HSA-159231)

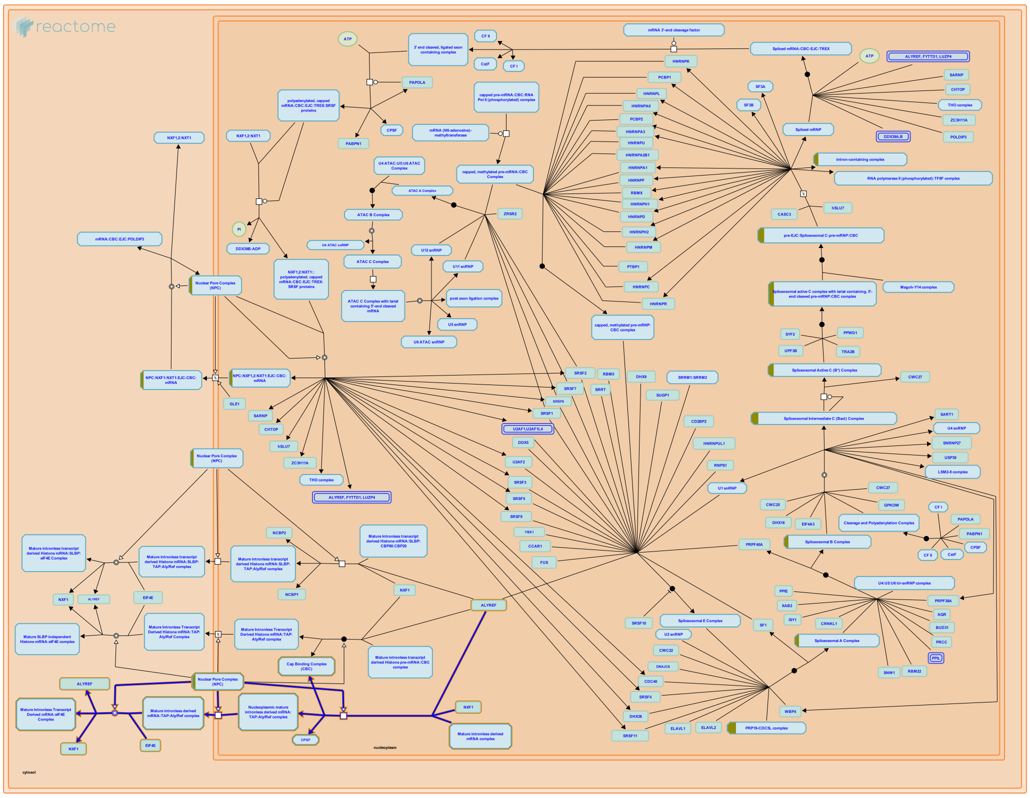

Cellular compartments: nucleoplasm, nuclear envelope, cytosol.

Transport of mRNA from the nucleus to the cytoplasm, where it is translated into protein, is highly selective and closely coupled to correct RNA processing.

References

Edit history

Date	Action	Author
2005-02-27	Created	Gillespie ME
2020-11-20	Modified	Shorser S

Entities found in this pathway (1)

Input	UniProt Id
POM121C	A8CG34, Q96HA1

22. Interactions of Rev with host cellular proteins (R-HSA-177243)

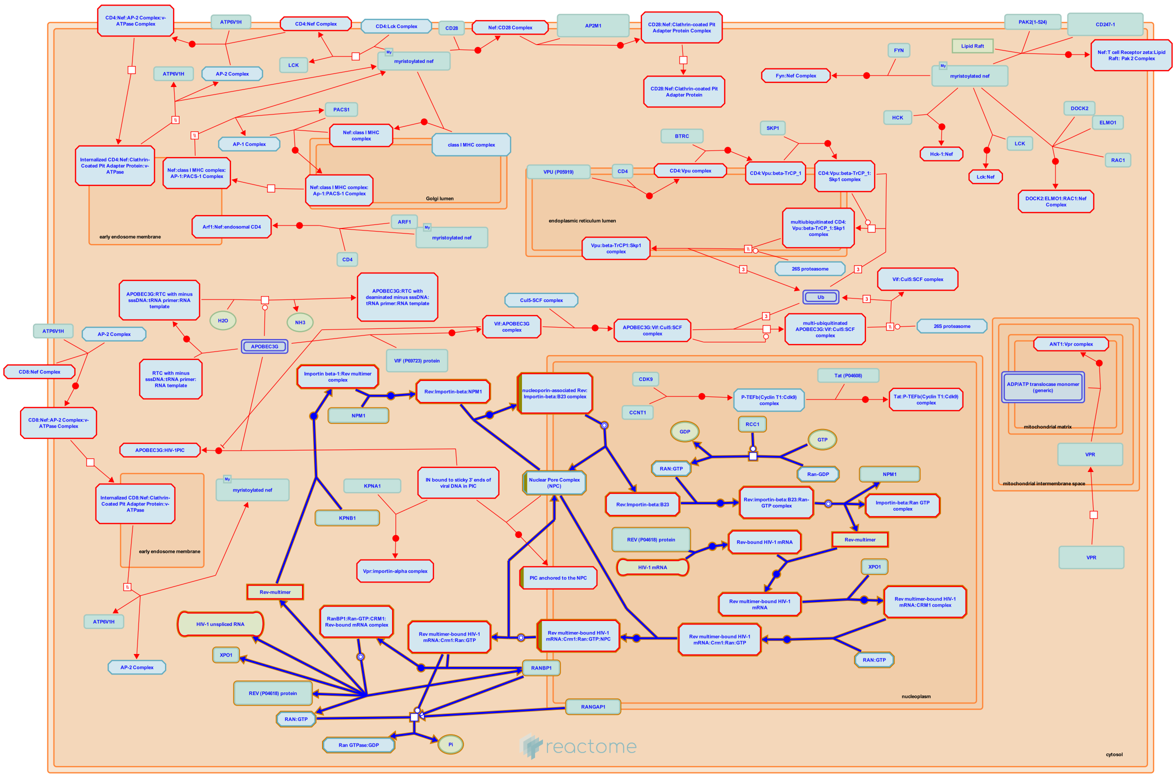

Cellular compartments: nucleoplasm.

Diseases: Human immunodeficiency virus infectious disease.

In order to facilitate the transport of incompletely spliced HIV-1 transcripts, Rev shuttles between the cytoplasm and nucleus using host cell transport mechanisms (reviewed in Li et al. 2005). Nuclear import appears to be achieved by the association of Rev with importin-beta and B23 and docking at the nuclear pore through interactions between importin-beta and nucleoporins. The dissociation of Rev with the import machinery and the subsequent export of Rev-associated HIV-1 mRNA complex requires Ran-GTP. Ran GTP associates with importin-beta, displacing its cargo. Crm1 associates with the Rev:RNA complex and Ran:GTP and is believed to interact with nucleoporins facilitating docking of the RRE-Rev-CRM1-RanGTP complex to the nuclear pore and the translocation of the complex across the nuclear pore complex. In the cytoplasm, RanBP1 associates with Ran-GTP causing the Crm1-Rev-Ran-GTP complex to disassemble. The Ran GAP protein promotes the hydrolysis of RanGTP to Ran GDP. The activities of Ran GAP in the cytoplasm and Ran-GEF, which converts RAN-GDP to Ran-GTP in the nucleus, produce a gradient of Ran-GTP/GDP required for this shuttling of Rev and other cellular transport proteins.

References

Askjaer P, Jensen TH, Nilsson J, Englmeier L & Kjems J (1998). The specificity of the CRM1-Rev nuclear export signal interaction is mediated by RanGTP. J Biol Chem, 273, 33414-22.

Edit history

Date	Action	Author
2005-07-27	Authored	Rice AP, Matthews L
2006-03-24	Edited	Matthews L

Date	Action	Author
2006-03-24	Created	Matthews L
2007-02-01	Reviewed	Kumar A
2016-11-08	Modified	Shorser S

Entities found in this pathway (1)

Input	UniProt Id
POM121C	A8CG34, Q96HA1

23. NS1 Mediated Effects on Host Pathways (R-HSA-168276)

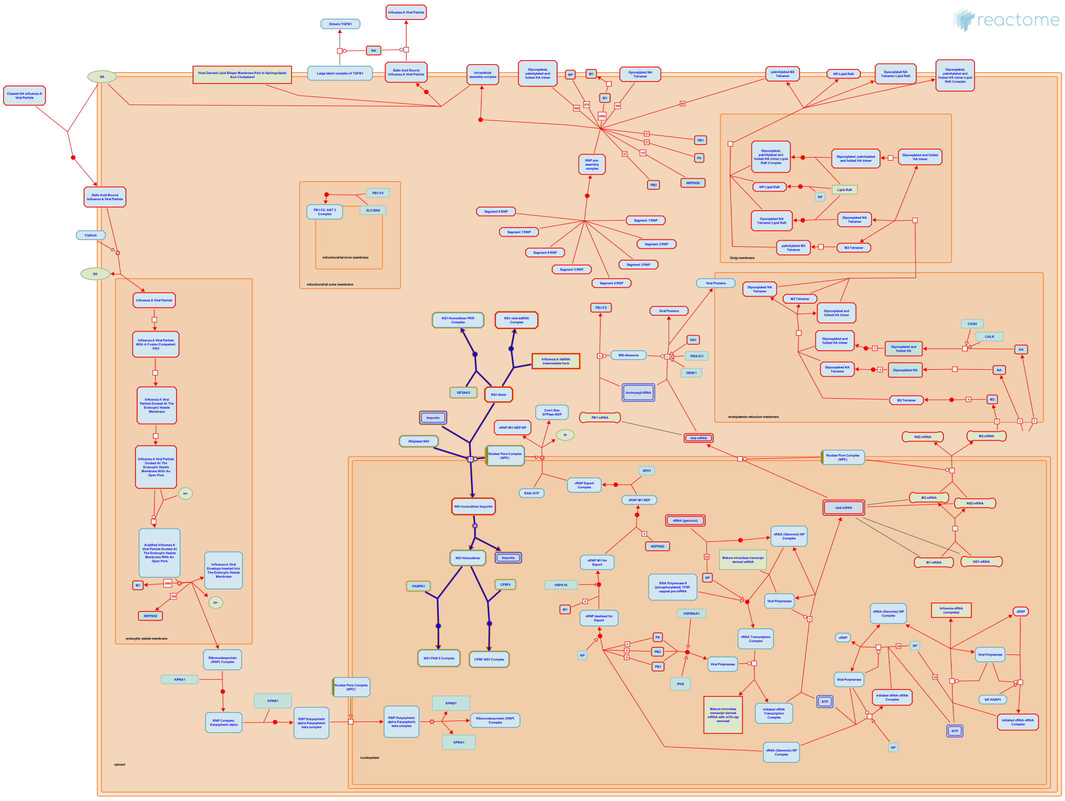

Diseases: influenza.

Viral NS1 protein is a nuclear, dimeric protein that is highly expressed in infected cells and has dsRNA-binding activity. The RNA-binding domain lies within the N-terminal portion of the protein. The NS1 RNA-binding domain forms a symmetric homodimer with a six-helical fold. Mutational analysis has demonstrated that dimer formation is crucial for RNA-binding. The basic residues are believed to make contact with the phosphate backbone of the RNA which is consistent with an observed lack of sequence specificity. Neither NS1 nor its bound RNA undergo any significant structural changes upon binding. The NS1 dimer spans the minor groove of canonical A-form dsRNA. The non-RNA binding portion of NS1 has been termed the effector domain and includes binding sites for host cell poly (A)-binding protein II (PABII) and the 30kDa subunit of cleavage and polyadenylation specificity factor (CPSF).

References

Palese P & Shaw ML (2001). Orthomyxoviridae: The Viruses and Their Replication. Fields Virology, 5th edition D.M. Knipe and P.M. Howley, Editors. 2006, Lippencott Williams and Wilkins: Philadelphia

ISBN-10: 0-7817-6060-7, 1647-1689.

Edit history

Date	Action	Author
2004-05-12	Reviewed	Gale M Jr
2005-11-14	Created	Gillespie ME
2013-11-18	Edited	Gillespie ME
2013-11-18	Authored	Gillespie ME

Date	Action	Author
2017-12-02	Modified	D'Eustachio P

Entities found in this pathway (1)

Input	UniProt Id
POM121C	A8CG34, Q96HA1

24. TP53 Regulates Metabolic Genes (R-HSA-5628897)

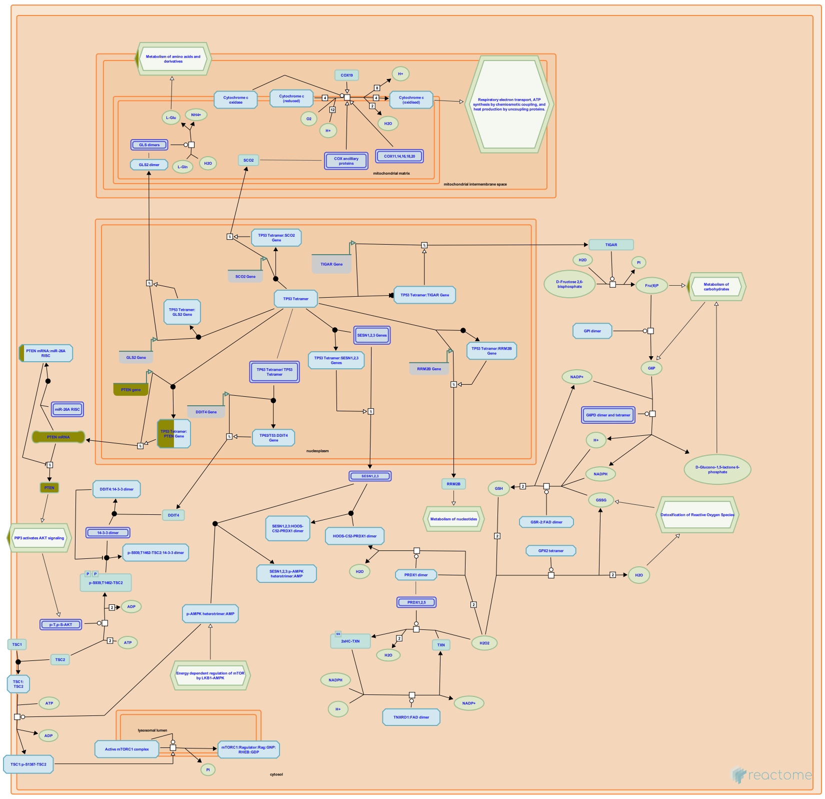

While the p53 tumor suppressor protein (TP53) is known to inhibit cell growth by inducing apoptosis, senescence and cell cycle arrest, recent studies have found that p53 is also able to influence cell metabolism to prevent tumor development. TP53 regulates transcription of many genes involved in the metabolism of carbohydrates, nucleotides and amino acids, protein synthesis and aerobic respiration.

TP53 stimulates transcription of TIGAR, a D-fructose 2,6-bisphosphatase. TIGAR activity decreases glycolytic rate and lowers ROS (reactive oxygen species) levels in cells (Bensaad et al. 2006). TP53 may also negatively regulate the rate of glycolysis by inhibiting the expression of glucose transporters GLUT1, GLUT3 and GLUT4 (Kondoh et al. 2005, Schwartzenberg-Bar-Yoseph et al. 2004, Kawauchi et al. 2008).

TP53 negatively regulates several key points in PI3K/AKT signaling and downstream mTOR signaling, decreasing the rate of protein synthesis and, hence, cellular growth. TP53 directly stimulates transcription of the tumor suppressor PTEN, which acts to inhibit PI3K-mediated activation of AKT (Stambolic et al. 2001). TP53 stimulates transcription of sestrin genes, SESN1, SESN2, and SESN3 (Velasco-Miguel et al. 1999, Budanov et al. 2002, Brynczka et al. 2007). One of sestrin functions may be to reduce and reactivate overoxidized peroxiredoxin PRDX1, thereby reducing ROS levels (Budanov et al. 2004, Papadia et al. 2008, Essler et al. 2009). Another function of sestrins is to bind the activated AMPK complex and protect it from AKT-mediated inactivation. By enhancing AMPK activity, sestrins negatively regulate mTOR signaling (Budanov and Karin 2008, Cam et al. 2014). The expression of DDIT4 (REDD1), another negative regulator of mTOR signaling, is directly stimulated by TP63 and TP53. DDIT4 prevents AKT-mediated inactivation of TSC1:TSC2 complex, thus inhibiting mTOR cascade (Cam et al. 2014, Ellisen et al. 2002, DeYoung et al. 2008). TP53 may also be involved, directly or indirectly, in regulation of expression of other participants of PI3K/AKT/mTOR signaling, such as PIK3CA (Singh et al. 2002), TSC2 and AMPKB (Feng et al. 2007).

TP53 regulates mitochondrial metabolism through several routes. TP53 stimulates transcription of SCO2 gene, which encodes a mitochondrial cytochrome c oxidase assembly protein (Matoba et al. 2006). TP53 stimulates transcription of RRM2B gene, which encodes a subunit of the ribonucleotide reductase complex, responsible for the conversion of ribonucleotides to deoxyribonucleotides and essential for the maintenance of mitochondrial DNA content in the cell (Tanaka et al. 2000, Bourdon et al. 2007, Kulawiec et al. 2009). TP53 also transactivates mitochondrial transcription factor A (TFAM), a nuclear-encoded gene important for mitochondrial DNA (mtDNA) transcription and maintenance (Park et al. 2009). Finally, TP53 stimulates transcription of the mitochondrial glutaminase GLS2, leading to increased mitochondrial respiration rate and reduced ROS levels (Hu et al. 2010).

The great majority of tumor cells generate energy through aerobic glycolysis, rather than the much more efficient aerobic mitochondrial respiration, and this metabolic change is known as the Warburg effect (Warburg 1956). Since the majority of tumor cells have impaired TP53 function, and TP53 regulates a number of genes involved in glycolysis and mitochondrial respiration, it is likely that TP53 inactivation plays an important role in the metabolic derangement of cancer cells such as the Warburg effect and the concomitant increased tumorigenicity (reviewed by Feng and Levine 2010). On the other hand, some mutations of TP53 in Li-Fraumeni syndrome may result in the retention of its wild-type metabolic activities while losing cell cycle and apoptosis functions (Wang et al. 2013). Consistent with such human data, some mutations of p53, unlike p53 null state, retain the ability to regulate energy metabolism while being inactive in regulating its classic gene targets involved in cell cycle, apoptosis and senescence. Retention of metabolic and antioxidant functions of p53 protects p53 mutant mice from early onset tumorigenesis (Li et al. 2012).

References

Feng Z & Levine AJ (2010). The regulation of energy metabolism and the IGF-1/mTOR pathways by the p53 protein. Trends Cell Biol., 20, 427-34.

WARBURG O (1956). On the origin of cancer cells. Science, 123, 309-14.

Bensaad K, Tsuruta A, Selak MA, Vidal MN, Nakano K, Bartrons R, ... Vousden KH (2006). TIGAR, a p53-inducible regulator of glycolysis and apoptosis. Cell, 126, 107-20.

Kondoh H, Lleonart ME, Gil J, Wang J, Degan P, Peters G, ... Beach D (2005). Glycolytic enzymes can modulate cellular life span. Cancer Res., 65, 177-85.

Schwartzenberg-Bar-Yoseph F, Armoni M & Karnieli E (2004). The tumor suppressor p53 down-regulates glucose transporters GLUT1 and GLUT4 gene expression. Cancer Res., 64, 2627-33.

Edit history

Date	Action	Author
2014-10-24	Created	Orlic-Milacic M
2014-12-23	Edited	Orlic-Milacic M
2014-12-23	Authored	Orlic-Milacic M
2014-12-30	Reviewed	Hwang PM, Kang JG, Wang PY
2016-02-04	Reviewed	Zaccara S, Inga A
2020-11-20	Modified	Shorser S

Entities found in this pathway (1)

Input	UniProt Id
PTEN	P60484

Input	Ensembl Id
PTEN	ENSG00000171862, ENST00000371953

25. Transport of Mature mRNAs Derived from Intronless Transcripts (R-HSA-159234)

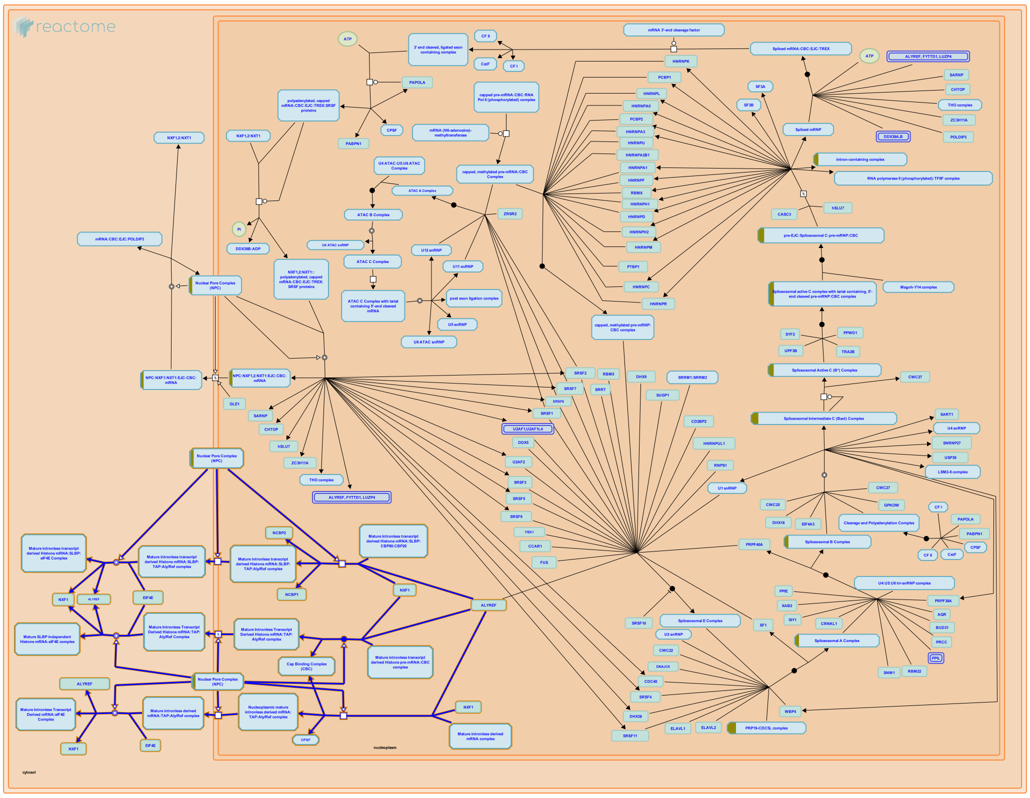

Cellular compartments: nucleoplasm, nuclear envelope, cytosol.

Transport of mature mRNAs derived from intronless transcripts require some of the same protein complexes as mRNAs derived from intron containing complexes, including TAP and Aly/Ref. However a number of the splicing related factors are lacking from the intronless derived mRNAs, as they required no splicing.

References

Edit history

Date	Action	Author
2005-02-27	Created	Gillespie ME
2005-03-13	Authored	Gillespie ME
2020-11-17	Edited	Gillespie ME
2020-11-20	Modified	Shorser S

Entities found in this pathway (1)

Input	UniProt Id
POM121C	A8CG34, Q96HA1

6. Identifiers found

Below is a list of the input identifiers that have been found or mapped to an equivalent element in Reactome, classified by resource.

Entities (33)

Input	UniProt Id	Input	UniProt Id	Input	UniProt Id
ADAMTS18	Q8TE60	ADAMTS5	Q9UNA0	AQP11	Q8NBQ7
ATP11C	Q8NB49	CAMSAP2	Q6UN15	CDK8	P49336
CHD9	Q3L8U1	DAPK1	P53355	DCUN1D4	Q92564
DLG2	Q15700	EPC1	Q9H2F5	EXOC5	O00471
FOXP1	Q9H334-8	FRAT2	O75474	GOLM1	Q8NBJ4
ICOS	Q9Y6W8-1	JARID2	Q92833	LRP4	O75096
MMS19	Q96T76	MTMR10	Q9NXD2	PDK4	Q16654
POM121C	A8CG34, Q96HA1	PPP2R5E	Q16537	PTEN	P60484
RAB30	Q15771	SLC25A37	Q9NYZ2	SLC2A14	Q8TDB8
SMS	P52788	STAG1	Q8WVM7	SYT2	Q8N9I0
TGFB2	P61812	TNFAIP3	P21580	ZBTB16	Q05516

Input	Ensembl Id
PTEN	ENST00000371953

7. Identifiers not found

These 21 identifiers were not found neither mapped to any entity in Reactome.

AMMECR1	AZI2	CNNM2	EMX2	LIN28B	OTUD4	PCGF3	PDIK1L
PRR14L	RNF38	RUNX1T1	SEC31B	SERBP1	SIPA1L2	SOX5	STOX2
TGFBR3	TIGD5	TRIB2	ZBTB37	ZCCHC2			
