# Supplementary material for: In silico detection of dysregulated genes and molecular pathways in Alzheimer’s disease as basis for food restoring approach
Source: PeerJ. 2025 Apr 7;13:e19100. doi: 10.7717/peerj.19100 (PMC11984471; doi:10.7717/peerj.19100)
Supplement: Supplemental Information 9 — The 25 most relevant pathways sorted by p-value, related to the target gene of up-regulated miRs in AD. [file peerj-13-19100-s009.pdf]

reactome

Pathway Analysis Report

This report contains the pathway analysis results for the submitted sample ''. Analysis was performed against Reactome version 75 on 17/03/2021. The web link to these results is:

https://reactome.org/PathwayBrowser/#/ANALYSIS=MjAyMTAzMTcwOTQ0MjdfNTI5MDc%3D

Please keep in mind that analysis results are temporarily stored on our server. The storage period depends on usage of the service but is at least 7 days. As a result, please note that this URL is only valid for a limited time period and it might have expired.

Table of Contents

1. Introduction
2. Properties
3. Genome-wide overview
4. Most significant pathways
5. Pathways details
6. Identifiers found
7. Identifiers not found

1. Introduction

Reactome is a curated database of pathways and reactions in human biology. Reactions can be considered as pathway 'steps'. Reactome defines a 'reaction' as any event in biology that changes the state of a biological molecule. Binding, activation, translocation, degradation and classical biochemical events involving a catalyst are all reactions. Information in the database is authored by expert biologists, entered and maintained by Reactome's team of curators and editorial staff. Reactome content frequently cross-references other resources e.g. NCBI, Ensembl, UniProt, KEGG (Gene and Compound), ChEBI, PubMed and GO. Orthologous reactions inferred from annotation for Homo sapiens are available for 17 non-human species including mouse, rat, chicken, puffer fish, worm, fly, yeast, rice, and Arabidopsis. Pathways are represented by simple diagrams following an SBGN-like format.

Reactome's annotated data describe reactions possible if all annotated proteins and small molecules were present and active simultaneously in a cell. By overlaying an experimental dataset on these annotations, a user can perform a pathway over-representation analysis. By overlaying quantitative expression data or time series, a user can visualize the extent of change in affected pathways and its progression. A binomial test is used to calculate the probability shown for each result, and the p-values are corrected for the multiple testing (Benjamini–Hochberg procedure) that arises from evaluating the submitted list of identifiers against every pathway.

To learn more about our Pathway Analysis, please have a look at our relevant publications:

Fabregat A, Sidiropoulos K, Garapati P, Gillespie M, Hausmann K, Haw R, … D'Eustachio P (2016). The reactome pathway knowledgebase. Nucleic Acids Research, 44(D1), D481–D487. https://doi.org/10.1093/nar/gkv1351.

Fabregat A, Sidiropoulos K, Viteri G, Forner O, Marin-Garcia P, Arnau V, … Hermjakob H (2017). Reactome pathway analysis: a high-performance in-memory approach. BMC Bioinformatics, 18.

2. Properties

- This is an **overrepresentation** analysis: A statistical (hypergeometric distribution) test that determines whether certain Reactome pathways are over-represented (enriched) in the submitted data. It answers the question 'Does my list contain more proteins for pathway X than would be expected by chance?' This test produces a probability score, which is corrected for false discovery rate using the Benjamani-Hochberg method.
- 8 out of 14 identifiers in the sample were found in Reactome, where 49 pathways were hit by at least one of them.
- All non-human identifiers have been converted to their human equivalent.
- This report is filtered to show only results for species 'Homo sapiens' and resource 'all resources'.
- The unique ID for this analysis (token) is MjAyMTAzMTcwOTQ0MjdfNTI5MDc%3D. This ID is valid for at least 7 days in Reactome's server. Use it to access Reactome services with your data.

3. Genome-wide overview

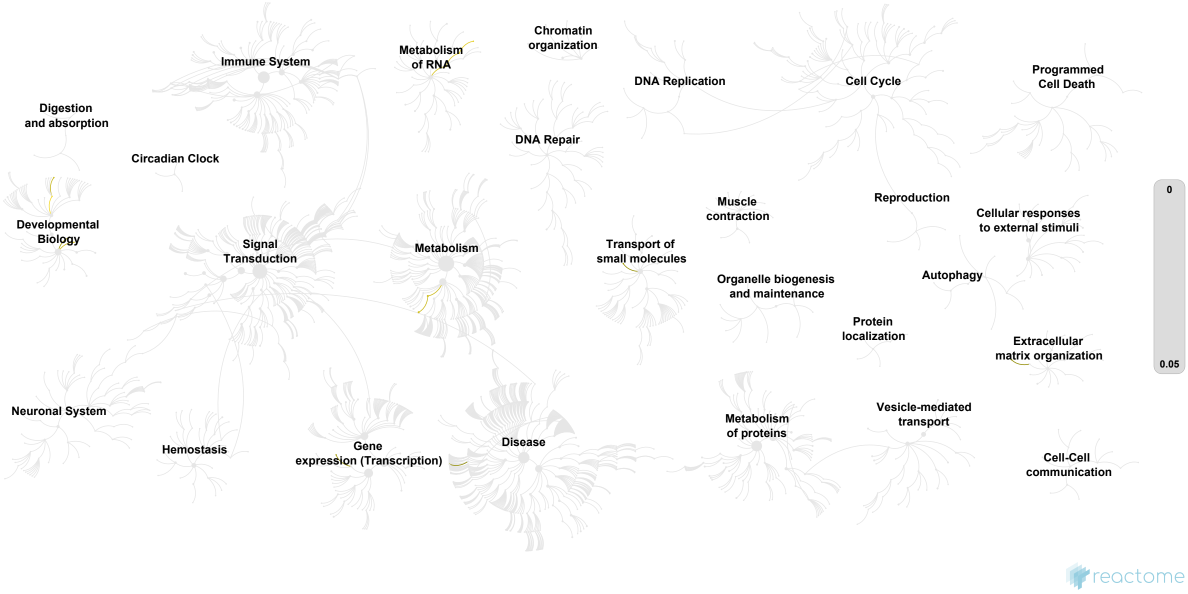

This figure shows a genome-wide overview of the results of your pathway analysis. Reactome pathways are arranged in a hierarchy. The center of each of the circular "bursts" is the root of one top-level pathway, for example "DNA Repair". Each step away from the center represents the next level lower in the pathway hierarchy. The color code denotes over-representation of that pathway in your input dataset. Light grey signifies pathways which are not significantly over-represented.

4. Most significant pathways

The following table shows the 25 most relevant pathways sorted by p-value.

Pathway name	Entities				Reactions	
	found	ratio	p-value	FDR*	found	ratio
Netrin-1 signaling	2 / 59	0.004	0.003	0.098	10 / 37	0.003
Formation of the Editosome	1 / 8	5.43e-04	0.01	0.098	2 / 2	1.51e-04
mRNA Editing: C to U Conversion	1 / 10	6.79e-04	0.013	0.098	3 / 3	2.27e-04
mRNA Editing	1 / 12	8.15e-04	0.015	0.098	3 / 9	6.80e-04
LGI-ADAM interactions	1 / 14	9.51e-04	0.018	0.098	4 / 5	3.78e-04
PPARA activates gene expression	2 / 174	0.012	0.021	0.098	1 / 41	0.003
Regulation of lipid metabolism by PPARalpha	2 / 176	0.012	0.021	0.098	1 / 44	0.003
DCC mediated attractive signaling	1 / 19	0.001	0.024	0.098	7 / 12	9.07e-04
Myogenesis	1 / 32	0.002	0.041	0.098	1 / 14	0.001
RNA Polymerase I Transcription Termination	1 / 33	0.002	0.042	0.098	3 / 4	3.02e-04
Miscellaneous transport and binding events	1 / 36	0.002	0.045	0.098	1 / 13	9.83e-04
Molecules associated with elastic fibres	1 / 38	0.003	0.048	0.098	2 / 10	7.56e-04
Defective B3GALTL causes Peters-plus syndrome (PpS)	1 / 39	0.003	0.049	0.098	1 / 1	7.56e-05
O-glycosylation of TSR domain-containing proteins	1 / 41	0.003	0.052	0.098	2 / 2	1.51e-04
Elastic fibre formation	1 / 46	0.003	0.058	0.098	2 / 17	0.001
RNA Polymerase I Transcription Initiation	1 / 50	0.003	0.063	0.098	3 / 6	4.54e-04
Extracellular matrix organization	2 / 330	0.022	0.067	0.098	4 / 319	0.024
mRNA Splicing - Minor Pathway	1 / 56	0.004	0.07	0.098	5 / 5	3.78e-04
E3 ubiquitin ligases ubiquitinate target proteins	1 / 61	0.004	0.076	0.098	2 / 16	0.001
B-WICH complex positively regulates rRNA expression	1 / 62	0.004	0.077	0.098	3 / 3	2.27e-04
RNA Polymerase I Promoter Escape	1 / 64	0.004	0.079	0.098	2 / 2	1.51e-04
Ion transport by P-type ATPases	1 / 76	0.005	0.094	0.098	2 / 15	0.001
Diseases associated with O-glycosylation of proteins	1 / 78	0.005	0.096	0.098	1 / 9	6.80e-04
Positive epigenetic regulation of rRNA expression	1 / 80	0.005	0.098	0.098	3 / 7	5.29e-04

Pathway name	Entities				Reactions	
	found	ratio	p-value	FDR*	found	ratio
NoRC negatively regulates rRNA expression	1 / 80	0.005	0.098	0.098	1 / 7	5.29e-04

* False Discovery Rate

5. Pathways details

For every pathway of the most significant pathways, we present its diagram, as well as a short summary, its bibliography and the list of inputs found in it.

1. Netrin-1 signaling (R-HSA-373752)

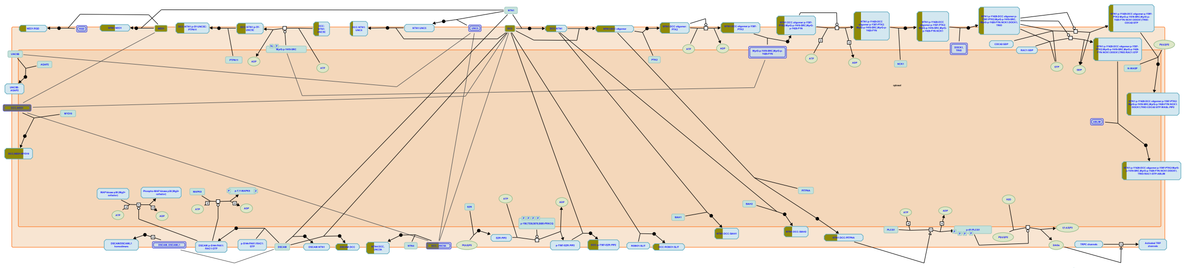

Netrins are secreted proteins that play a crucial role in neuronal migration and in axon guidance during the development of the nervous system. To date, several Netrins have been described in mouse and humans: Netrin-1, -3/NTL2, -4/h and G-Netrins. Netrin-1 is the most studied member of the family and has been shown to play a crucial role in neuronal navigation during nervous system development mainly through its interaction with its receptors DCC and UNC5. Members of the Deleted in colorectal cancer (DCC) family- which includes DCC and Neogenin in vertebrates- mediate netrin-induced axon attraction, whereas the C. elegans UNC5 receptor and its four vertebrate homologs Unc5a-Unc5d mediate repulsion.

References

Moore SW, Tessier-Lavigne M & Kennedy TE (2007). Netrins and their receptors. Adv Exp Med Biol, 621, 17-31.

Cooper HM, Gad JM & Keeling SL (1999). The Deleted in Colorectal Cancer netrin guidance system: a molecular strategy for neuronal navigation. Clin Exp Pharmacol Physiol, 26, 749-51.

Edit history

Date	Action	Author
2008-07-16	Authored	Garapati P V
2008-07-16	Created	Garapati P V
2008-07-30	Edited	Garapati P V
2010-02-16	Reviewed	Cooper HM
2020-11-20	Modified	Shorser S

Entities found in this pathway (1)

Input	UniProt Id
NEO1	P43146, Q92859

2. Formation of the Editosome (R-HSA-75094)

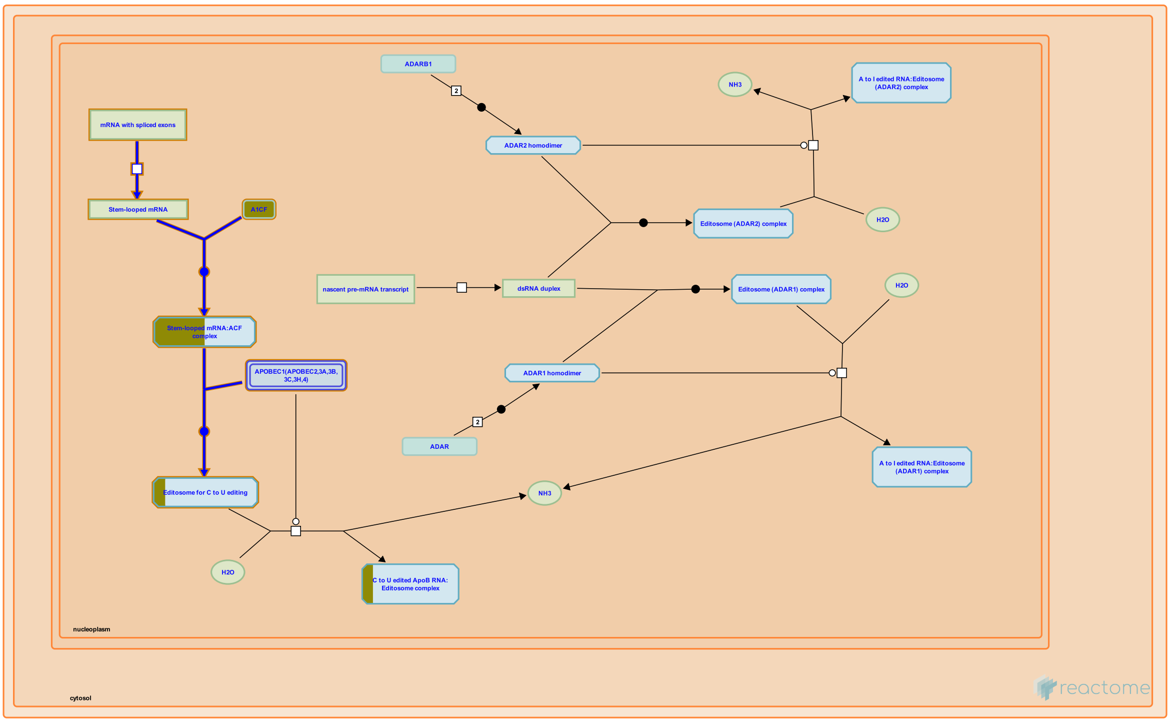

Cellular compartments: nucleoplasm.

The editosome for C to U editing in mammals consist of a member of cytidine deaminase family of enzymes, apoB mRNA editig catalytic polypeptide 1 (APOBEC-1) and a complementing specificity factor (ACF) in addition to the target mRNA.

References

Edit history

Date	Action	Author
2003-08-22	Created	Carmichael GG
2003-12-05	Authored	Gopinathrao G
2020-11-20	Modified	Shorser S

Entities found in this pathway (1)

Input	UniProt Id
A1CF	Q9NQ94

3. mRNA Editing: C to U Conversion (R-HSA-72200)

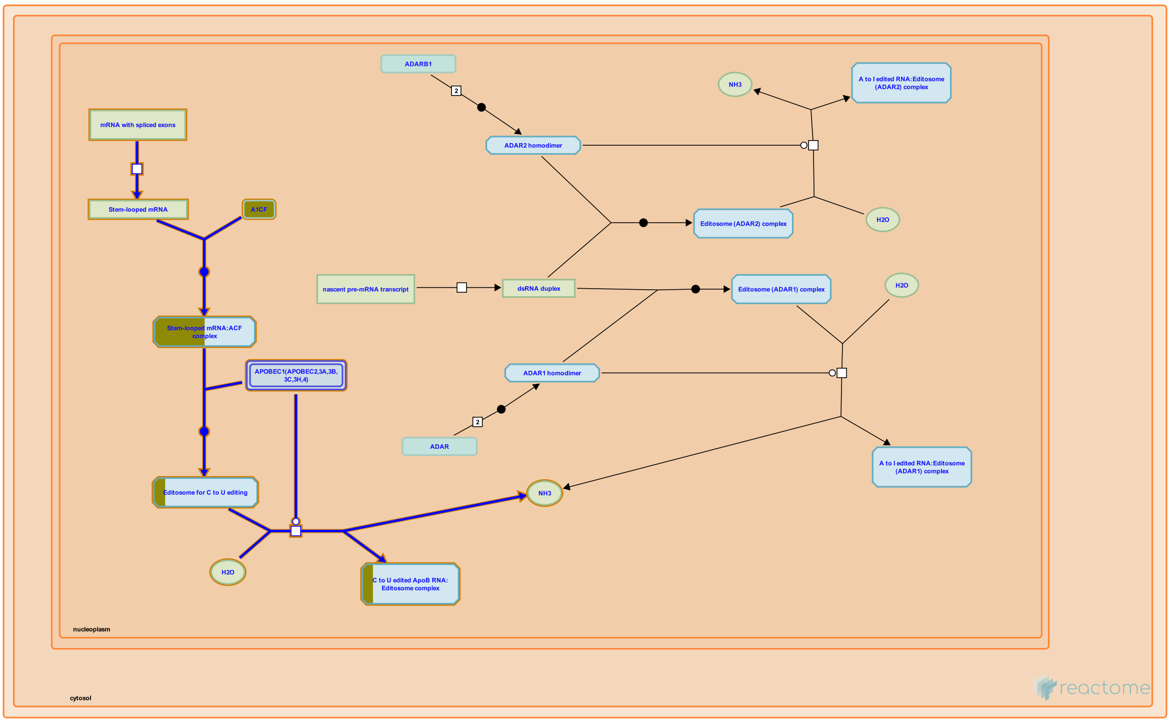

Cellular compartments: nucleoplasm.

The best characterized case of C to U editing is in the intestinal apolipoprotein B transcript, where the editing event creates a premature translation stop codon and consequently leads to a shorter form of the protein. In the liver, C to U editing is important in the expression of specific isoforms of the apolipoprotein B enzyme. ApoB mRNA editing is a posttranscriptional, nuclear process that can be initiated after splicing, at the time of polyadenylation and is completed by the time pre-mRNA matures fully (reviewed by Blanc and Davidson, 2003).

This editing event is a simple hydrolytic cytidine deamination to uridine, and is carried out by the Apobec-1 enzyme, along with the Apobec-1 complementing factor, ACF. The editing of apo-B mRNA involves the site-specific deamination of (C6666 to U), which converts codon 2153 from a glutamine codon, CAA, to a premature stop codon, UAA. As ACF is distributed in a variety of tissues, and these genes contain multiple family members, it is possible that editing events in additional targets will be found.

The cis-acting regulatory elements for C to U editing include: 22 nt editing site within ApoB mRNA, 5' tripartite motif with an enhancer element adjacent to the target cytidine, a spacer element and mooring sequence both 3' to the cytidine (reviewed by Smith et al., 1997).

References

Gott JM & Emeson RB (2001). Functions and mechanisms of RNA editing. Annu Rev Genet, 34, 499-531.

Blanc V & Davidson NO (2003). C-to-U RNA editing: mechanisms leading to genetic diversity. J Biol Chem, 278, 1395-8.

Wedekind JE, Dance GS, Sowden MP & Smith HC (2003). Messenger RNA editing in mammals: new members of the APOBEC family seeking roles in the family business. Trends Genet, 19, 207-16.

Chester A, Scott J, Anant S & Navaratnam N (2000). RNA editing: cytidine to uridine conversion in apolipoprotein B mRNA. Biochim Biophys Acta, 1494, 1-13.

Edit history

Date	Action	Author
2003-08-22	Created	Carmichael GG
2020-11-20	Modified	Shorser S

Entities found in this pathway (1)

Input	UniProt Id
A1CF	Q9NQ94

4. mRNA Editing (R-HSA-75072)

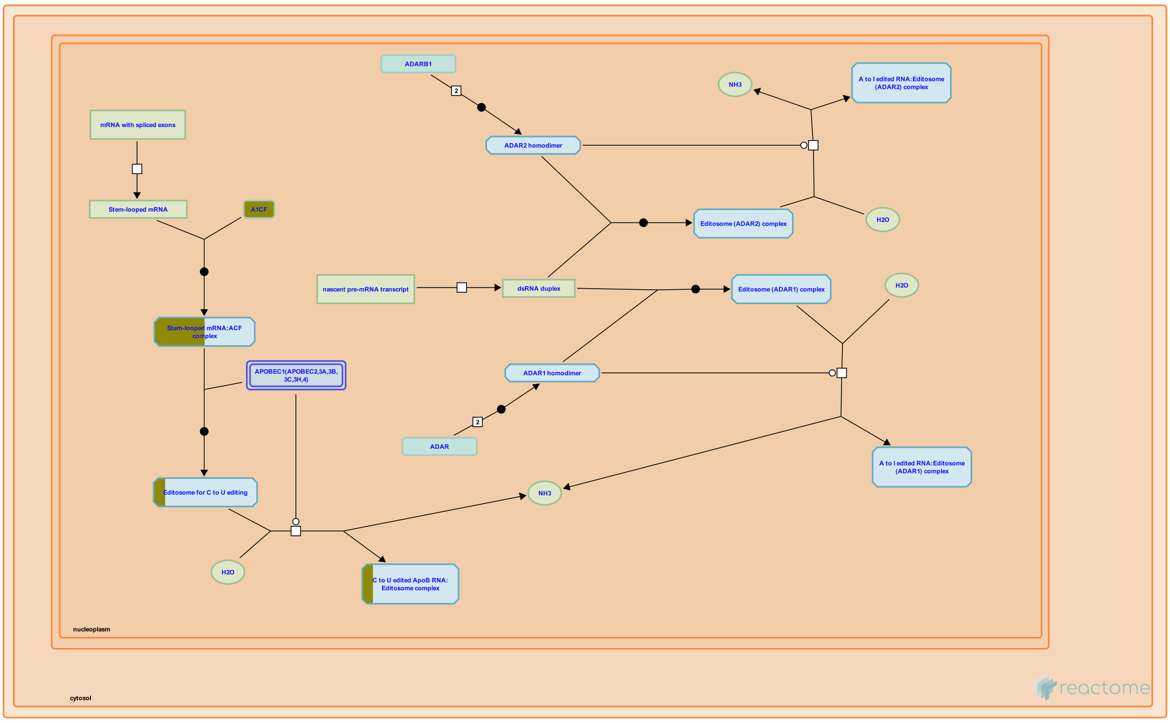

Cellular compartments: nucleoplasm.

After transcription, some RNA molecules are altered to contain bases not encoded in the genome. Most often this involves the editing or modification of one base to another, but in some organisms can involve the insertion or deletion of a base. Such editing events alter the coding properties of mRNA.

RNA editing can be generally defined as the co- or post transcriptional modification of the primary sequence of RNA from that encoded in the genome through nucleotide deletion, insertion, or base modification mechanisms.

There are two pathways of RNA editing: the substitution/conversion pathway and the insertion/deletion pathway. The insertion/deletion editing occurs in protozoans like Trypanosoma, Leishmania; in slime molds like Physarum spp., and in some viral categories like paramyxoviruses, Ebola virus etc. To date, the substitution/conversion pathway has been observed in human along with other mammals, Drosophila, and some plants. The RNA editing processes are known to create diversity in proteins involved in various pathways like lipid transport, metabolism etc. and may act as potential targets for therapeutic intervention (Smith et al., 1997).

The reaction mechanisms of cytidine and adenosine deaminases is represented below. In both these reactions, NH3 is presumed to be released:

References

Stuart K & Panigrahi AK (2002). RNA editing: complexity and complications. Mol Microbiol, 45, 591-6.

Gott JM & Emeson RB (2001). Functions and mechanisms of RNA editing. Annu Rev Genet, 34, 499-531.

Edit history

Date	Action	Author
2003-08-22	Authored	Carmichael GG
2003-08-22	Created	Carmichael GG
2020-11-17	Edited	Gopinathrao G
2020-11-20	Modified	Shorser S

Entities found in this pathway (1)

Input	UniProt Id
A1CF	Q9NQ94

5. LGI-ADAM interactions (R-HSA-5682910)

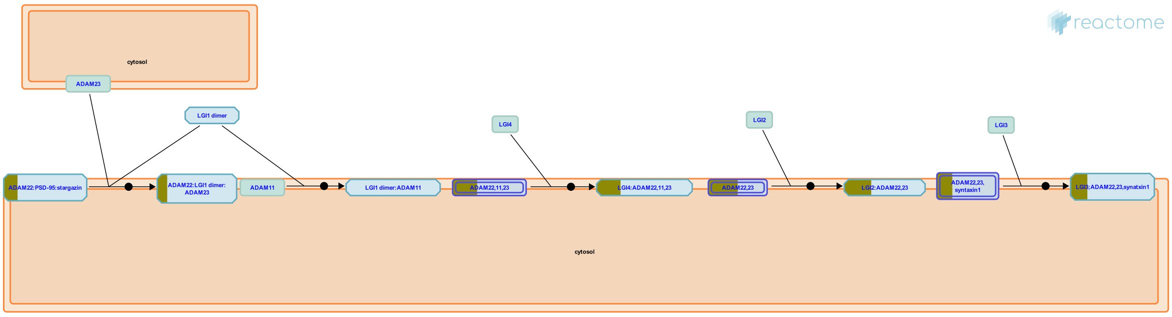

Cellular compartments: extracellular region.

Synapse formation and maturation require multiple interactions between presynaptic and postsynaptic neurons. These interactions are mediated by a diverse set of synaptogenic proteins (Kegel et al. 2013, Siddiqui & Craig 2011). Initial synapse formation needs both the binding of secreted proteins to presynaptic and postsynaptic receptors, and the direct binding between presynaptic and postsynaptic transmembrane proteins. One class of molecules that plays an important role in cellular interactions in nervous system development and function is the leucine-rich glioma inactivated (LGI) protein family. These are secreted synaptogenic proteins consisting of an LRR (leucine-rich repeat) domain and a epilepsy-associated or EPTP (epitempin) domain (Gu et al. 2002). Both protein domains are generally involved in protein-protein interactions. Genetic and biochemical evidence suggests that the mechanism of action of LGI proteins involves binding to a subset of cell surface receptors belonging to the ADAM (a disintegrin and metalloproteinase) family, i.e. ADAM11, ADAM22 and ADAM23. These interactions play crucial role in the development and function of the vertebrate nervous system mainly mediating synaptic transmission and myelination (Kegel et al. 2013, Novak 2004, Seals & Courtneidge 2003).

References

Kegel L, Aunin E, Meijer D & Bermingham JR (2013). LGI proteins in the nervous system. ASN Neuro, 5, 167-81.

Edit history

Date	Action	Author
2015-03-11	Edited	Garapati P V
2015-03-11	Authored	Garapati P V
2015-03-11	Created	Garapati P V
2015-04-20	Reviewed	Meijer D
2020-11-20	Modified	Shorser S

Entities found in this pathway (1)

Input	UniProt Id
MMD	Q9P0K1

6. PPARA activates gene expression (R-HSA-1989781)

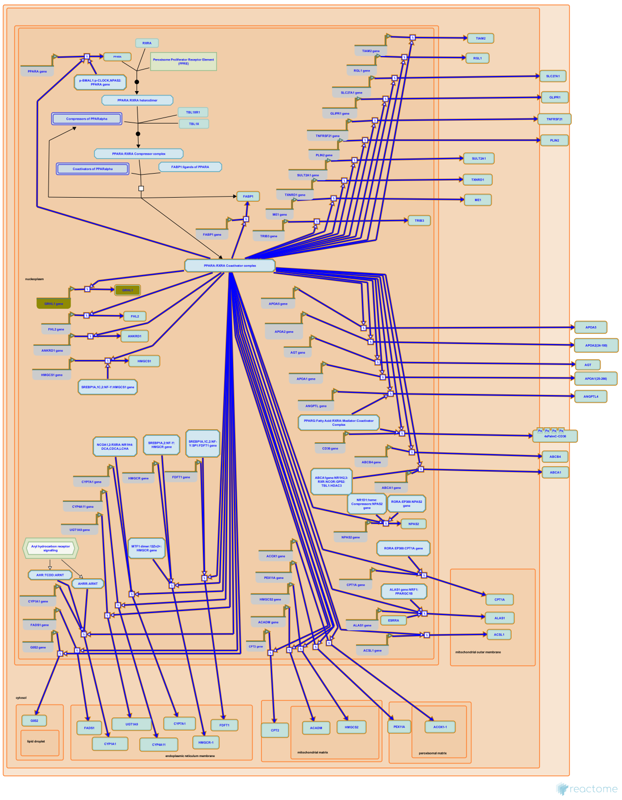

Cellular compartments: cytosol, endoplasmic reticulum membrane, extracellular region, lipid droplet, mitochondrial inner membrane, mitochondrial matrix, mitochondrial outer membrane, nucleoplasm, peroxisomal matrix, peroxisomal membrane, plasma membrane.

The set of genes regulated by PPAR-alpha is not fully known in humans, however many examples have been found in mice. Genes directly activated by PPAR-alpha contain peroxisome proliferator receptor elements (PPREs) in their promoters and include:

1) genes involved in fatty acid oxidation and ketogenesis (Acox1, Cyp4a, Acadm, Hmgcs2);

2) genes involved in fatty acid transport (Cd36, , Slc27a1, Fabp1, Cpt1a, Cpt2);

3) genes involved in producing fatty acids and very low density lipoproteins (Me1, Scd1);

4) genes encoding apolipoproteins (Apoa1, Apoa2, Apoa5);

5) genes involved in triglyceride clearance (Angptl4);

6) genes involved in glycerol metabolism (Gpd1 in mouse);

7) genes involved in glucose metabolism (Pdk4);

8) genes involved in peroxisome proliferation (Pex11a);

9) genes involved in lipid storage (Plin, Adfp).

Many other genes are known to be regulated by PPAR-alpha but whether their regulation is direct or indirect remains to be found. These genes include: ACACA, FAS, SREBP1, FADS1, DGAT1, ABCA1, PLTP, ABCB4, UGT2B4, SULT2A1, Pnpla2, Acsl1, Slc27a4, many Acot genes, and others (reviewed in Rakhshandehroo et al. 2010).

References

Kersten S (2008). Peroxisome proliferator activated receptors and lipoprotein metabolism. PPAR Res, 2008, 132960.

Mandard S, Muller M & Kersten S (2004). Peroxisome proliferator-activated receptor alpha target genes. Cell Mol Life Sci, 61, 393-416.

Qi C, Zhu Y & Reddy JK (2000). Peroxisome proliferator-activated receptors, coactivators, and downstream targets. Cell Biochem Biophys, 32, 187-204.

Desvergne B & Wahli W (1999). Peroxisome proliferator-activated receptors: nuclear control of metabolism. Endocr Rev, 20, 649-88.

Rakhshandehroo M, Knoch B, Müller M & Kersten S (2010). Peroxisome proliferator-activated receptor alpha target genes. PPAR Res, 2010.

Edit history

Date	Action	Author
2009-06-08	Reviewed	Kersten S
2011-11-08	Edited	May B
2011-11-08	Authored	May B
2011-11-13	Created	May B
2020-11-10	Modified	D'Eustachio P

Entities found in this pathway (1)

Input	UniProt Id
GRHL1	Q9NZI5

Input	Ensembl Id
GRHL1	ENSG00000134317

7. Regulation of lipid metabolism by PPARalpha (R-HSA-400206)

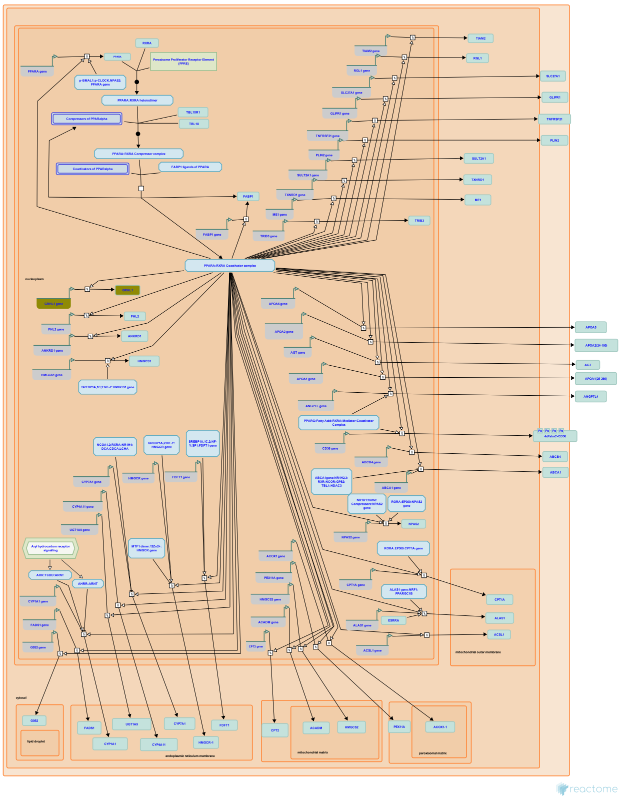

Cellular compartments: nucleoplasm, cytosol.

Peroxisome proliferator-activated receptor alpha (PPAR-alpha) is the major regulator of fatty acid oxidation in the liver. PPARalpha is also the target of fibrate drugs used to treat abnormal plasma lipid levels.

PPAR-alpha is a type II nuclear receptor (its subcellular location does not depend on ligand binding). PPAR-alpha forms heterodimers with Retinoid X receptor alpha (RXR-alpha), another type II nuclear receptor. PPAR-alpha is activated by binding fatty acid ligands, especially polyunsaturated fatty acids having 18-22 carbon groups and 2-6 double bonds.

The PPAR-alpha:RXR-alpha heterodimer binds peroxisome proliferator receptor elements (PPREs) in and around target genes. Binding of fatty acids and synthetic ligands causes a conformational change in PPAR-alpha such that it releases the corepressors and binds coactivators (CBP-SRC-HAT complex, ASC complex, and TRAP-Mediator complex) which initiate transcription of the target genes.

Target genes of PPAR-alpha participate in fatty acid transport, fatty acid oxidation, triglyceride clearance, lipoprotein production, and cholesterol homeostasis.

References

Feige JN, Gelman L, Michalik L, Desvergne B & Wahli W (2006). From molecular action to physiological outputs: peroxisome proliferator-activated receptors are nuclear receptors at the crossroads of key cellular functions. Prog Lipid Res, 45, 120-59.

Kersten S (2008). Peroxisome proliferator activated receptors and lipoprotein metabolism. PPAR Res, 2008, 132960.

Gouni-Berthold I & Krone W (2005). Peroxisome proliferator-activated receptor alpha (PPARalpha) and athero-sclerosis. Curr Drug Targets Cardiovasc Haematol Disord, 5, 513-23.

Desvergne B & Wahli W (1999). Peroxisome proliferator-activated receptors: nuclear control of metabolism. Endocr Rev, 20, 649-88.

Edit history

Date	Action	Author
2009-03-24	Created	May B
2009-05-30	Edited	May B
2009-05-30	Authored	May B
2009-06-08	Edited	May B
2009-06-08	Reviewed	Kersten S
2011-11-08	Edited	May B
2011-11-13	Revised	May B
2020-11-20	Modified	Shorser S

Entities found in this pathway (1)

Input	UniProt Id
GRHL1	Q9NZI5

Input	Ensembl Id
GRHL1	ENSG00000134317

8. DCC mediated attractive signaling (R-HSA-418885)

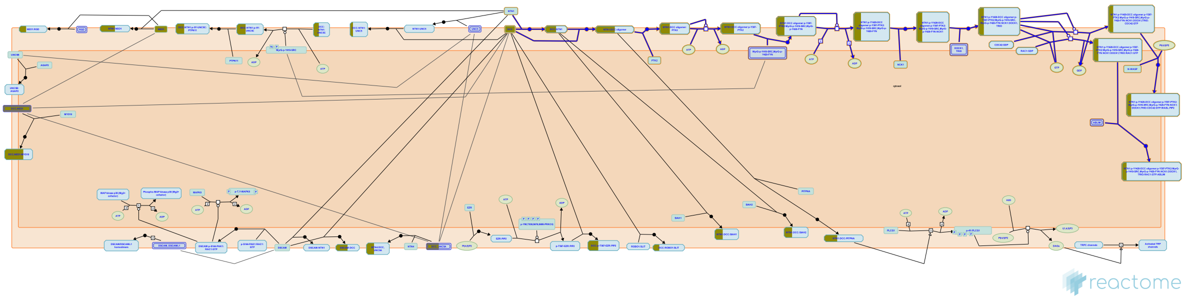

Cellular compartments: plasma membrane.

The DCC family includes DCC and neogenin in vertebrates. DCC is required for netrin-induced axon attraction. DCC is a transmembrane protein lacking any identifiable catalytic activity. Protein tyrosine kinase 2/FAK and src family kinases bind constitutively to the cytoplasmic domain of DCC and their activation couples to downstream intracellular signaling complex that directs the organization of actin.

References

Round J & Stein E (2007). Netrin signaling leading to directed growth cone steering. Curr Opin Neurobiol, 17, 15-21.

Ren XR, Ming GL, Xie Y, Hong Y, Sun DM, Zhao ZQ, ... Xiong WC (2004). Focal adhesion kinase in netrin-1 signaling. Nat Neurosci, 7, 1204-12.

Stein E, Zou Y, Poo M & Tessier-Lavigne M (2001). Binding of DCC by netrin-1 to mediate axon guidance independent of adenosine A2B receptor activation. Science, 291, 1976-82.

Shekarabi M & Kennedy TE (2002). The netrin-1 receptor DCC promotes filopodia formation and cell spreading by activating Cdc42 and Rac1. Mol Cell Neurosci, 19, 1-17.

Barallobre MJ, Pascual M, Del Rio JA & Soriano E (2005). The Netrin family of guidance factors: emphasis on Netrin-1 signalling. Brain Res Brain Res Rev, 49, 22-47.

Edit history

Date	Action	Author
2008-07-16	Authored	Garapati P V
2008-07-30	Edited	Garapati P V
2009-04-27	Created	Garapati P V
2010-02-16	Reviewed	Cooper HM
2020-11-24	Modified	Shorser S

Entities found in this pathway (1)

Input	UniProt Id
NEO1	P43146

9. Myogenesis (R-HSA-525793)

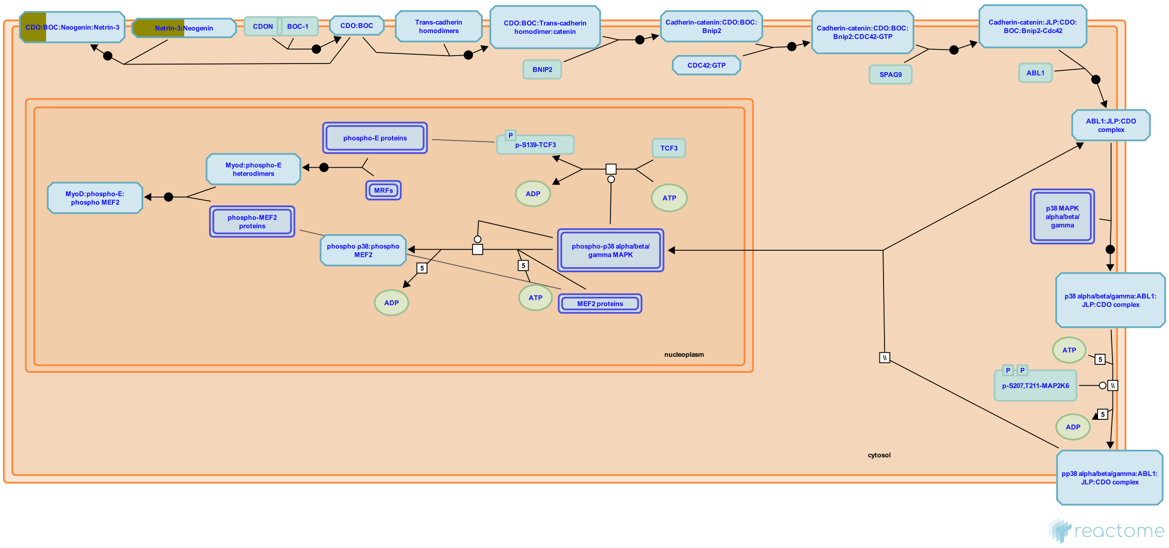

Myogenesis, the formation of muscle tissue, is a complex process involving steps of cell proliferation mediated by growth factor signaling, cell differentiation, reorganization of cells to form myotubes, and cell fusion. Here, one regulatory feature of this process has been annotated, the signaling cascade initiated by CDO (cell-adhesion-molecule-related/downregulated by oncogenes) and associated co-receptors.

CDO/Cdon is a type I transmembrane multifunctional co-receptor consisting of five immunoglobulin and three fibronectin type III (FNIII) repeats in the extracellular domain, and an intracellular domain with no identifiable motifs. It has been implicated in enhancing muscle differentiation in promyogenic cells. CDO exert its promyogenic effects as a component of multiprotein complexes that include the closely related factor Boc, the Ig superfamily receptor neogenin and its ligand netrin-3, and the adhesion molecules N- and M-cadherin. CDO modulates the Cdc42 and p38 mitogen-activated protein kinase (MAPK) pathways via a direct association with two scaffold-type proteins, JLP and Bnip-2, to regulate activities of myogenic bHLH factors and myogenic differentiation. CDO activates myogenic bHLH factors via enhanced heterodimer formation, most likely by inducing hyper-phosphorylation of E proteins.

Myogenic basic helix-loop-helix (bHLH) proteins are master regulatory proteins that activate the transcription of many muscle-specific genes during myogenesis. These myogenic bHLH proteins also referred to as MyoD family includes four members, MyoD, myogenin, myf5 and MRF4. These myogenic factors dimerize with E-proteins such as E12/E47, ITF-2 and HEB to form heterodimeric complexes that bind to a conserved DNA sequence known as the E box, which is present in the promoters and enhancers of most muscle-specific genes. Myocyte enhancer binding factor 2 (MEF2), which is a member of the MADS box family, also plays an important role in muscle differentiation. MEF2 activates transcription by binding to the consensus sequence, called the MEF2-binding site, which is also found in the control regions of numerous muscle-specific genes. MEF2 and myogenic bHLH proteins synergistically activate expression of muscle-specific genes via protein-protein interactions between DNA-binding domains of these heterologous classes of transcription factors. Members of the MyoD and MEF2 family of transcription factors associate combinatorially to control myoblast specification, differentiation and proliferation.

References

Krauss RS, Cole F, Gaio U, Takaesu G, Zhang W & Kang JS (2005). Close encounters: regulation of vertebrate skeletal myogenesis by cell-cell contact. J Cell Sci, 118, 2355-62.

Edit history

Date	Action	Author
2010-02-09	Reviewed	Krauss RS
2010-02-16	Edited	Garapati P V
2010-02-16	Authored	Garapati P V
2010-02-22	Created	Matthews L
2020-11-20	Modified	Shorser S

Entities found in this pathway (1)

Input	UniProt Id
NEO1	Q92859

10. RNA Polymerase I Transcription Termination (R-HSA-73863)

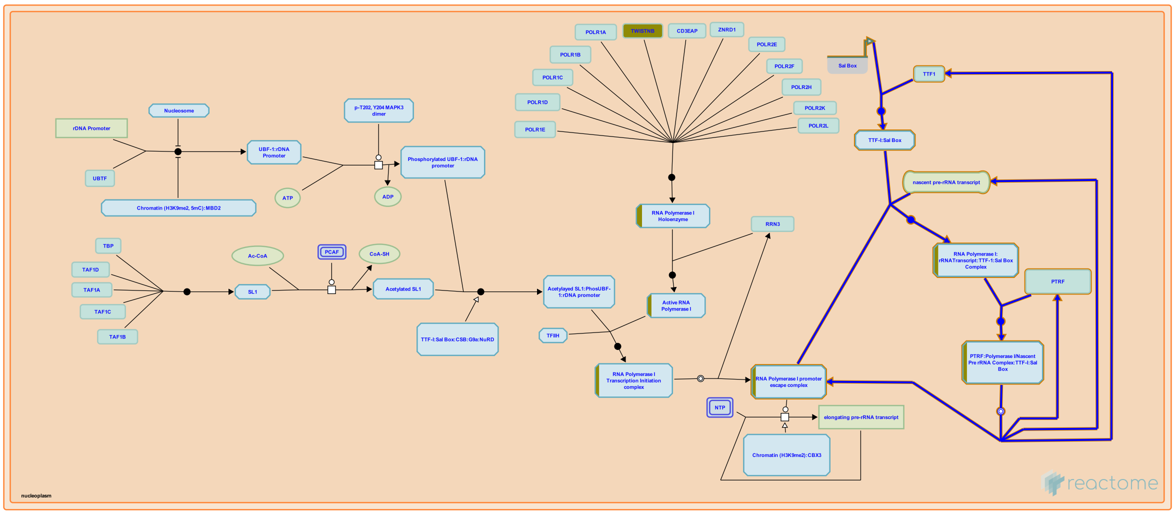

Cellular compartments: nucleolus.

Termination of transcription by RNA polymerase I is a 4 step process. Initially TTF-1 binds the template rDNA. This complex pauses polymerase I allowing PTRF to interact with the quaternary complex releasing both pre-rRNA and Pol I from the template and TTF-1.

References

Comai L (2004). Mechanism of RNA polymerase I transcription. Adv. Protein Chem., 67, 123-55.

Edit history

Date	Action	Author
2003-07-03	Authored	Comai L
2003-07-03	Created	Comai L
2020-11-17	Edited	Gillespie ME
2020-11-20	Modified	Shorser S

Entities found in this pathway (1)

Input	UniProt Id
MMD	Q3B726

11. Miscellaneous transport and binding events (R-HSA-5223345)

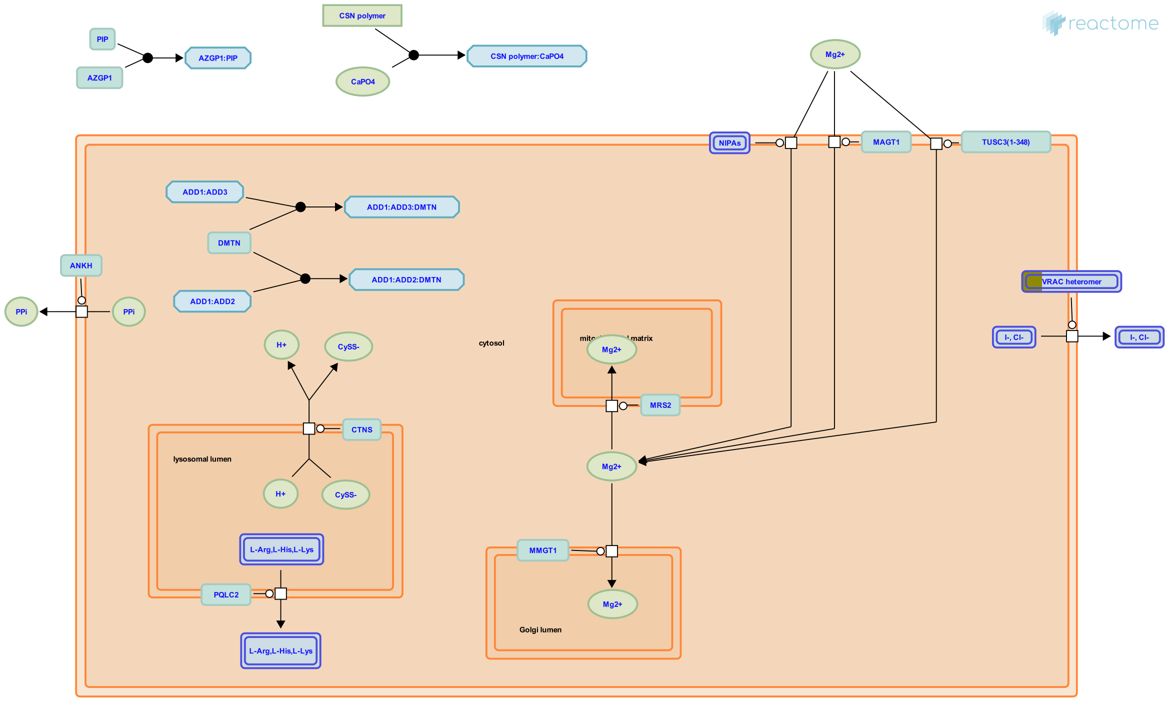

This section contains known transport and binding events that as of yet cannot be placed in exisiting pathways (Purves 2001, He et al. 2009, Rees et al. 2009).

References

Purves D (2001). *Chapter 4: Channels and Transporters, Neuroscience (2nd ed.)* .

He L, Vasiliou K & Nebert DW (2009). Analysis and update of the human solute carrier (SLC) gene superfamily. Hum Genomics, 3, 195-206.

Rees DC, Johnson E & Lewinson O (2009). ABC transporters: the power to change. Nat Rev Mol Cell Biol, 10, 218-27.

Edit history

Date	Action	Author
2014-01-06	Edited	Jassal B
2014-01-06	Authored	Jassal B
2014-01-06	Created	Jassal B
2015-02-11	Reviewed	D'Eustachio P
2020-11-20	Modified	Shorser S

Entities found in this pathway (1)

Input	UniProt Id
LRRC8B	Q6P9F7

12. Molecules associated with elastic fibres (R-HSA-2129379)

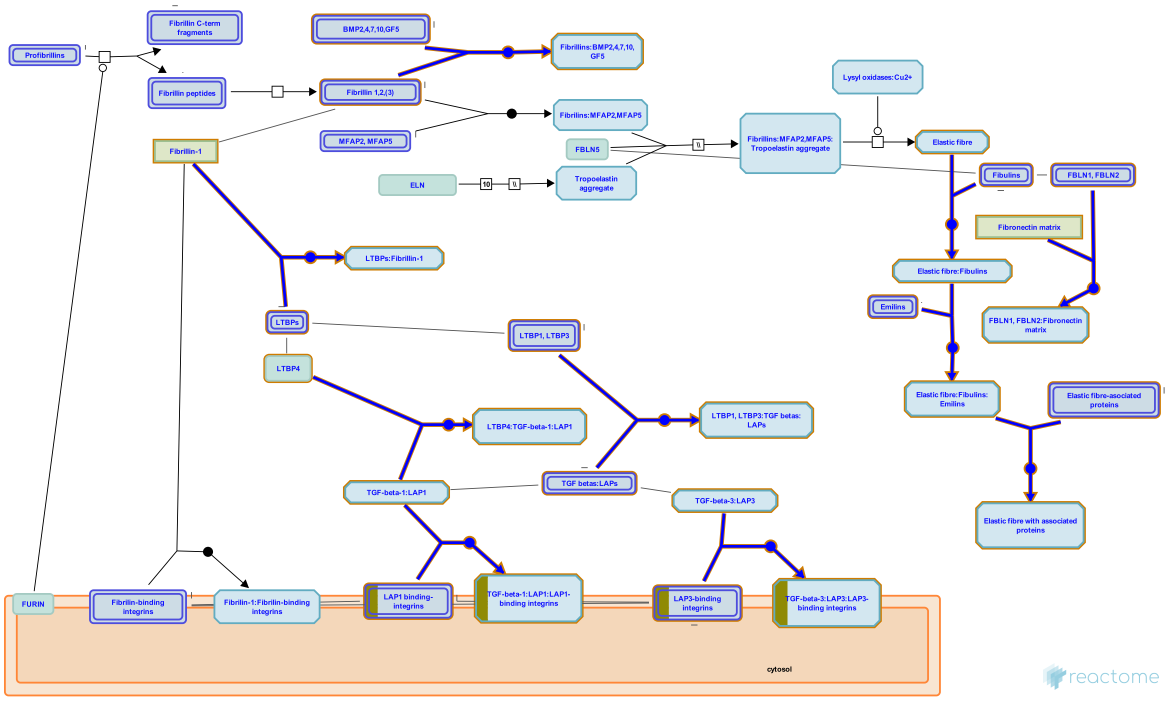

Proteins found associated with microfibrils include vitronectin (Dahlback et al. 1990), latent transforming growth factor beta-binding proteins (Kielty et al. 2002, Munger & Sheppard 2011), emilin (Bressan et al. 1993, Mongiat et al. 2000), members of the microfibrillar-associated proteins (MFAPs, Gibson et al.1996), and fibulins (Roark et al. 1995, Yanagisawa et al. 2002). The significance of these interactions is not well understood but may help mediate elastin-fibrillin interactions during elastic fibre assembly.

Proteoglycans such as versican (Isogai et al. 2002), biglycan, and decorin (Reinboth et al. 2002) can interact with the microfibrils. They confer specific properties including hydration, impact absorption, molecular sieving, regulation of cellular activities, mediation of growth factor association, and release and transport within the extracellular matrix (Buczek-Thomas et al. 2002). In addition, glycosaminoglycans have been shown to interact with tropoelastin through its lysine side chains (Wu et al. 1999) regulating tropoelastin assembly (Tu and Weiss, 2008).

References

Wagenseil JE & Mecham RP (2007). New insights into elastic fiber assembly. Birth Defects Res. C Embryo Today, 81, 229-40.

Kielty CM, Sherratt MJ & Shuttleworth CA (2002). Elastic fibres. J Cell Sci, 115, 2817-28.

Edit history

Date	Action	Author
2012-02-21	Created	Jupe S
2012-04-30	Authored	Jupe S
2012-11-02	Reviewed	Muiznieks LD
2012-11-12	Edited	Jupe S

Date	Action	Author
2020-11-20	Modified	Shorser S

Entities found in this pathway (1)

Input	UniProt Id
MMD	P26012

13. Defective B3GALTL causes Peters-plus syndrome (PpS) (R-HSA-5083635)

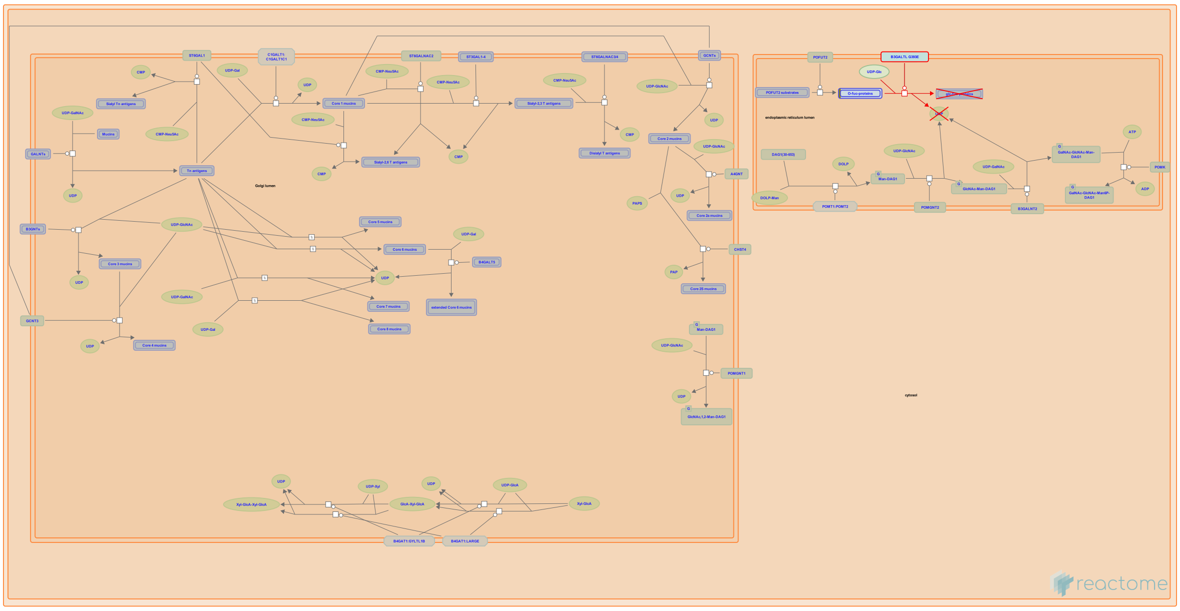

Diseases: eye disease, orofacial cleft.

Human beta-1,3-glucosyltransferase like protein (B3GALTL, HGNC Approved Gene Symbol: B3GLCT; MIM:610308; CAZy family GT31), localised on the ER membrane, glucosylates O-fucosylated proteins. The resultant glc-beta-1,3-fuc disaccharide modification on thrombospondin type 1 repeat (TSR1) domain-containing proteins is thought to assist in the secretion of many of these proteins from the ER lumen, and mediate an ER quality-control mechanism of folded TSRs (Vasudevan et al. 2015). Defects in B3GALTL can cause Peters plus syndrome (PpS; MIM:261540), an autosomal recessive disorder characterised by anterior eye chamber defects, short stature, delay in growth and mental developmental and cleft lip and/or palate (Heinonen & Maki 2009).

References

Heinonen TY & Maki M (2009). Peters'-plus syndrome is a congenital disorder of glycosylation caused by a defect in the beta1,3-glucosyltransferase that modifies thrombospondin type 1 repeats. Ann. Med., 41, 2-10.

Vasudevan D, Takeuchi H, Johar SS, Majerus E & Haltiwanger RS (2015). Peters plus syndrome mutations disrupt a noncanonical ER quality-control mechanism. Curr. Biol., 25, 286-95.

Edit history

Date	Action	Author
2013-11-07	Edited	Jassal B
2013-11-07	Authored	Jassal B
2013-11-07	Created	Jassal B
2015-12-18	Modified	Jassal B
2015-12-18	Reviewed	Hansen L, Joshi HJ

Entities found in this pathway (1)

Input	UniProt Id
ADAMTS9	Q9P2N4

14. O-glycosylation of TSR domain-containing proteins (R-HSA-5173214)

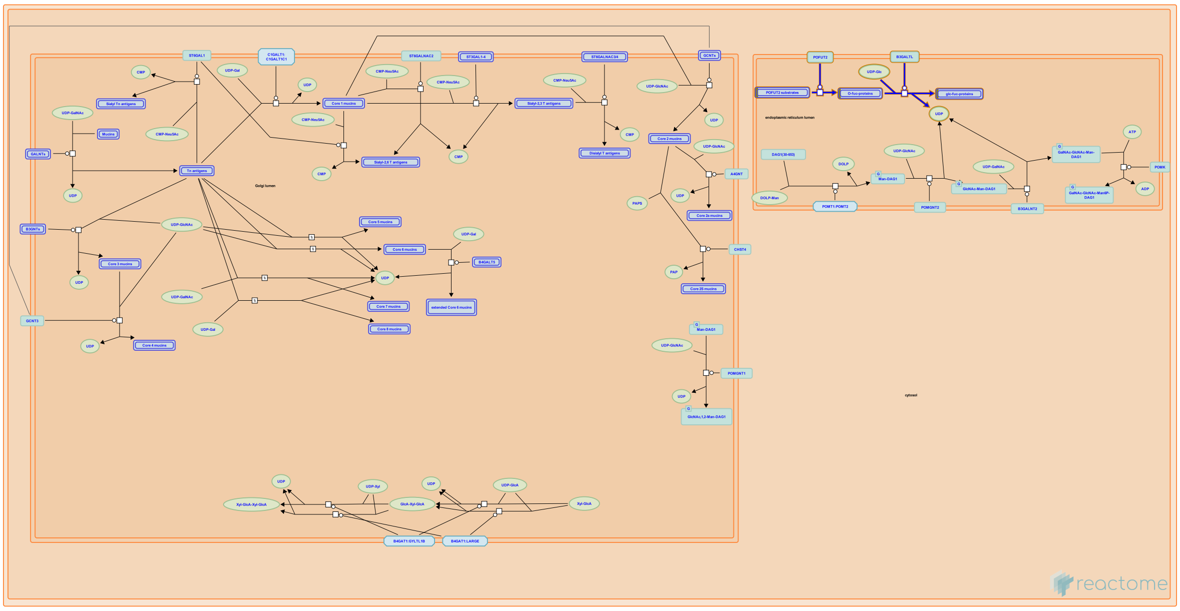

The O-fucosylation of proteins containing thrombospondin type 1 repeat (TSR) domains is an important PTM, regulating many biological processes such as Notch signalling, inflammation, wound healing, angiogenesis amd neoplasia (Adams & Tucker 2000, Moremen et al. 2012). Fucose addition is carried out by two protein fucosyltransferases, POFUT1 and 2. Only POFUT2 recognises the consensus sequence CSXS/TCG found in TSR1 domains and the fucosyl residue is attached to the hydroxyl group of conserved serine (S) or threonine (T) residues within the consensus sequence. The modification was first demonstrated on thrombospondin 1, found in platelets and the ECM (Hofsteenge et al. 2001, Luo et al. 2006). The resulting O-fucosyl-protein is subsequently a substrate for beta-1,3-glucosyltransferase-like protein (B3GALTL), which adds a glucosyl moiety to form the rare disaccharide modification Glc-beta-1,3-Fuc. More than 60 human proteins contain TSR1 domains, The disaccharide modification has been demonstrated on a small number of these TSR1 domain-containing proteins such as thrombospondin 1 (Hofsteenge et al. 2001, Luo et al. 2006), properdin (Gonzalez de Peredo et al. 2002) and F-spondin (Gonzalez de Peredo et al. 2002). The ADAMTS (a disintegrin-like and metalloprotease domain with thrombospondin type-1 repeats) superfamily consists of 19 secreted metalloproteases (ADAMTS proteases) and at lease five ADAMTS-like proteins in humans. Five members of the ADAMTS superfamily have also had experimental confirmation of the disaccharide modification. Examples are ADAMTS13 (Ricketts et al. 2007) and ADAMTSL1 (Wang et al. 2007). In the two reactions described here, the TSR1 domain-containing proteins with similarity to the experimentally confirmed ones are included as putative substrates.

References

Adams JC & Tucker RP (2000). The thrombospondin type 1 repeat (TSR) superfamily: diverse proteins with related roles in neuronal development. Dev. Dyn., 218, 280-99.

Moremen KW, Tiemeyer M & Nairn AV (2012). Vertebrate protein glycosylation: diversity, synthesis and function. Nat. Rev. Mol. Cell Biol., 13, 448-62.

Hofsteenge J, Huwiler KG, Macek B, Hess D, Lawler J, Mosher DF & Peter-Katalinic J (2001). C-mannosylation and O-fucosylation of the thrombospondin type 1 module. J. Biol. Chem., 276, 6485-98.

Luo Y, Nita-Lazar A & Haltiwanger RS (2006). Two distinct pathways for O-fucosylation of epidermal growth factor-like or thrombospondin type 1 repeats. J. Biol. Chem., 281, 9385-92.

Ricketts LM, Dlugosz M, Luther KB, Haltiwanger RS & Majerus EM (2007). O-fucosylation is required for ADAMTS13 secretion. J. Biol. Chem., 282, 17014-23.

Edit history

Date	Action	Author
2013-11-25	Edited	Jassal B
2013-11-25	Authored	Jassal B
2013-11-25	Created	Jassal B
2014-02-07	Reviewed	D'Eustachio P
2020-11-20	Modified	Shorser S

Entities found in this pathway (1)

Input	UniProt Id
ADAMTS9	Q9P2N4

15. Elastic fibre formation (R-HSA-1566948)

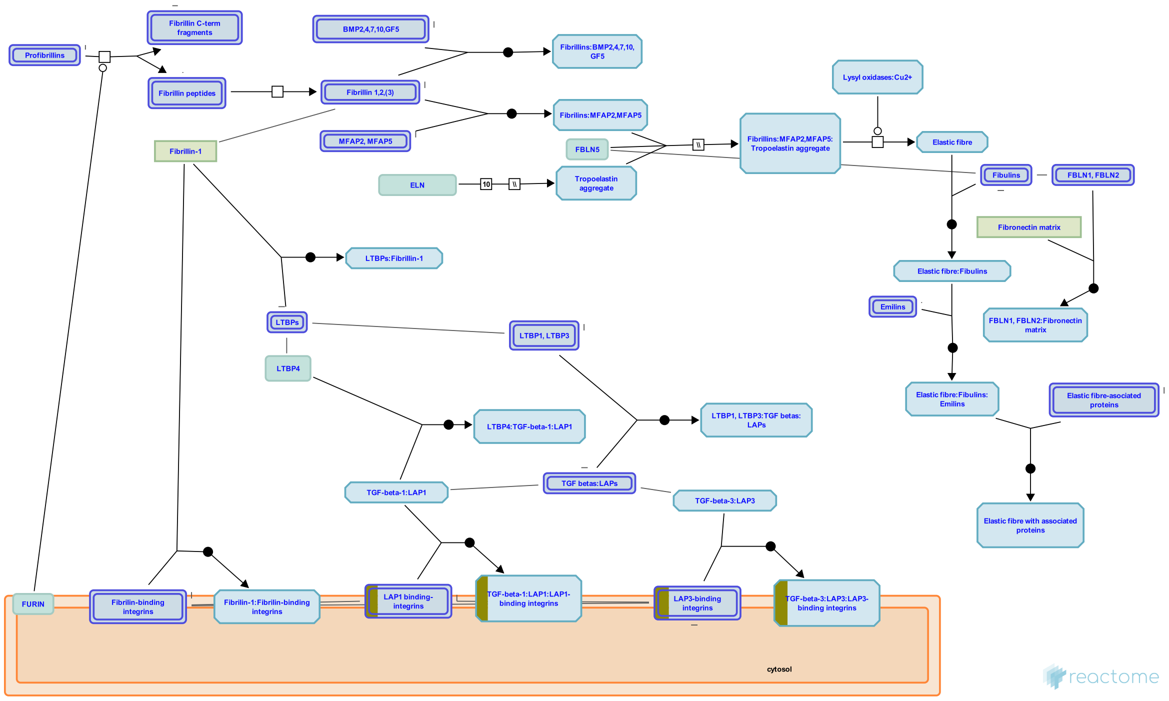

Cellular compartments: extracellular region.

Elastic fibres (EF) are a major structural constituent of dynamic connective tissues such as large arteries and lung parenchyma, where they provide essential properties of elastic recoil and resilience. EF are composed of a central cross-linked core of elastin, surrounded by a mesh of microfibrils, which are composed largely of fibrillin. In addition to elastin and fibrillin-1, over 30 ancillary proteins are involved in mediating important roles in elastic fibre assembly as well as interactions with the surrounding environment. These include fibulins, elastin microfibril interface located proteins (EMILINs), microfibril-associated glycoproteins (MAGPs) and Latent TGF-beta binding proteins (LTBPs). Fibulin-5 for example, is expressed by vascular smooth muscle cells and plays an essential role in the formation of elastic fibres through mediating interactions between elastin and fibrillin (Yanigasawa et al. 2002, Freeman et al. 2005). In addition, it plays a role in cell adhesion through integrin receptors and has been shown to influence smooth muscle cell proliferation (Yanigasawa et al. 2002, Nakamura et al. 2002). EMILINs are a family of homologous glycoproteins originally identified in extracts of aortas. Found at the elastin-fibrillin interface, early studies showed that antibodies to EMILIN can affect the process of elastic fibre formation (Bressan et al. 1993). EMILIN1 has been shown to bind elastin and fibulin-5 and appears to coordinate their common interaction (Zanetti et al. 2004). MAGPs are found to co-localize with microfibrils. MAGP-1, for example, binds strongly to an N-terminal sequence of fibrillin-1. Other proteins found associated with microfibrils include vitronectin (Dahlback et al. 1990).

Fibrillin is most familiar as a component of elastic fibres but microfibrils with no elastin are found in the ciliary zonules of the eye and invertebrate circulatory systems. The addition of elastin to microfibrils is a vertebrate adaptation to high pulsatile pressures in their closed circulatory systems (Faury et al. 2003). Elastin appears to have emerged after the divergence of jawless vertebrates from other vertebrates (Sage 1982).

Fibrillin-1 is the major structural component of microfibrils. Fibrillin-2 is expressed earlier in development than fibrillin-1 and may be important for elastic fiber formation (Zhang et al. 1994). Fibrillin-3 arose as a duplication of fibrillin-2 that did not occur in the rodent lineage. It was first isolated from human brain (Corson et al. 2004).

Fibrillin assembly is not as well defined as elastin assembly. The primary structure of fibrillin is dominated by calcium binding epidermal growth factor like repeats (Kielty et al. 2002). Fibrillin may form dimers or trimers before secretion. However, multimerisation predominantly occurs outside the cell. Formation of fibrils appears to require cell surface structures suggesting an involvement of cell surface receptors. Fibrillin is assembled pericellularly (i.e. on or close to the cell surface) into microfibrillar arrays that undergo time dependent maturation into microfibrils with beaded-string appearance. Transglutaminase forms gamma glutamyl epsilon lysine isopeptide bonds within or between peptide chains. Additionally, intermolecular disulfide bond formation between fibrillins is an important contributor to fibril maturation (Reinhardt et al. 2000).

Models of fibrillin-1 microfibril structure suggest that the N-terminal half of fibrillin-1 is asymmetrically exposed in outer filaments, while the C-terminal half is buried in the interior (Kuo et al. 2007). Fibrillinopathies include Marfan syndrome, familial ectopia lentis, familial thoracic aneurysm, all due to mutations in the fibrillin-1 gene FBN1, and congenital contractural arachnodactyly which is caused by mutation of FBN2 (Maslen & Glanville 1993, Davis & Summers 2012).

In vivo assembly of fibrillin requires the presence of extracellular fibronectin fibres (Sabatier et al. 2009). Fibrillins have Arg-Gly-Asp (RGD) sequences that interact with integrins (Pfaff et al. 1996, Sakamoto et al. 1996, Bax et al., 2003, Jovanovic et al. 2008) and heparin-binding domains that interact with a cell-surface heparan sulfate proteoglycan (Tiedemann et al. 2001) possibly a syndecan (Ritty et al. 2003). Fibrillins also have a major role in binding and sequestering growth factors such as TGF beta into the ECM (Neptune et al. 2003). Proteoglycans such as versican (Isogai et al. 2002), biglycan, and decorin (Reinboth et al. 2002) can interact with the microfibrils. They confer specific properties including hydration, impact absorption, molecular sieving, regulation of cellular activities, mediation of growth factor association, and release and transport within the extracellular matrix (Buczek-Thomas et al. 2002). In addition, glycosaminoglycans have been shown to interact with tropoelastin through its lysine side chains (Wu et al. 1999), regulating tropoelastin assembly (Tu & Weiss 2008).

Elastin is synthesized as a 70kDa monomer called tropoelastin, a highly hydrophobic protein composed largely of two types of domains that alternate along the polypeptide chain. Hydrophobic domains are rich in glycine, proline, alanine, leucine and valine. These amino acids occur in characteristic short (3-9 amino acids) tandem repeats, with a flexible and highly dynamic structure (Floquet et al. 2004). Unlike collagen, glycine in elastin is not rigorously positioned every 3 residues. However, glycine is distributed frequently throughout all hydrophobic domains of elastin, and displays a strong preference for inter-glycine spacing of 0-3 residues (Rauscher et al. 2006).

Elastic fibre formation involves the deposition of tropoelastin onto a template of fibrillin rich microfibrils. Recent results suggest that the first step of elastic fiber formation is the organization of small globules of elastin on the cell surface followed by globule aggregation into microfibres (Kozel et al. 2006). An important contribution to the initial stages assembly is thought to be made by the intrinsic ability of the protein to direct its own polymeric organization in a process termed 'coacervation' (Bressan et al. 1986). This self-assembly process appears to be determined by interactions between hydrophobic domains (Bressan et al. 1986, Vrhovski et al. 1997, Bellingham et al. 2003, Cirulis & Keeley 2010) which result in alignment of the cross-linking domains, allowing the stabilization of elastin through the formation of cross-links generated through the oxidative deamination of lysine residues, catalyzed by members of the lysyl oxidase (LOX) family (Reiser et al. 1992, Mithieux & Weiss 2005). The first step in the cross-linking reaction is the oxidative formation of the delta aldehyde, known as alpha aminoadipic semialdehyde or allysine (Partridge 1963). Subsequent reactions that are probably spontaneous lead to the formation of cross-links through dehydrolysinonorleucine and allysine aldol, a trifunctional cross-link dehydromerodesmosine and two tetrafunctional cross-links desmosine and isodesmosine (Lucero & Kagan 2006), which are unique to elastin. These cross-links confer mechanical integrity and high durability. In addition to their role in self-assembly, hydrophobic domains provide elastin with its elastomeric properties, with initial studies suggesting that the elastomeric propereties of elastin are driven through changes in entropic interactions with surrounding water molecules (Hoeve & Flory 1974).

A very specific set of proteases, broadly grouped under the name elastases, is responsible for elastin remodelling (Antonicelli et al. 2007). The matrix metalloproteinases (MMPs) are particularly important in elastin breakdown, with MMP2, 3, 9 and 12 explicitly shown to degrade elastin (Ra & Parks 2007). Nonetheless, elastin typically displays a low turnover rate under normal conditions over a lifetime (Davis 1993).

References

Kozel BA, Rongish BJ, Czirok A, Zach J, Little CD, Davis EC, ... Mecham RP (2006). Elastic fiber formation: a dynamic view of extracellular matrix assembly using timer reporters. J Cell Physiol , 207, 87-96.

Edit history

Date	Action	Author
2011-09-06	Created	Jupe S
2012-04-30	Authored	Jupe S
2012-11-02	Reviewed	Muiznieks LD
2012-11-12	Edited	Jupe S
2013-02-27	Reviewed	Parkinson J
2020-11-20	Modified	Shorser S

Entities found in this pathway (1)

Input	UniProt Id
MMD	P26012

16. RNA Polymerase I Transcription Initiation (R-HSA-73762)

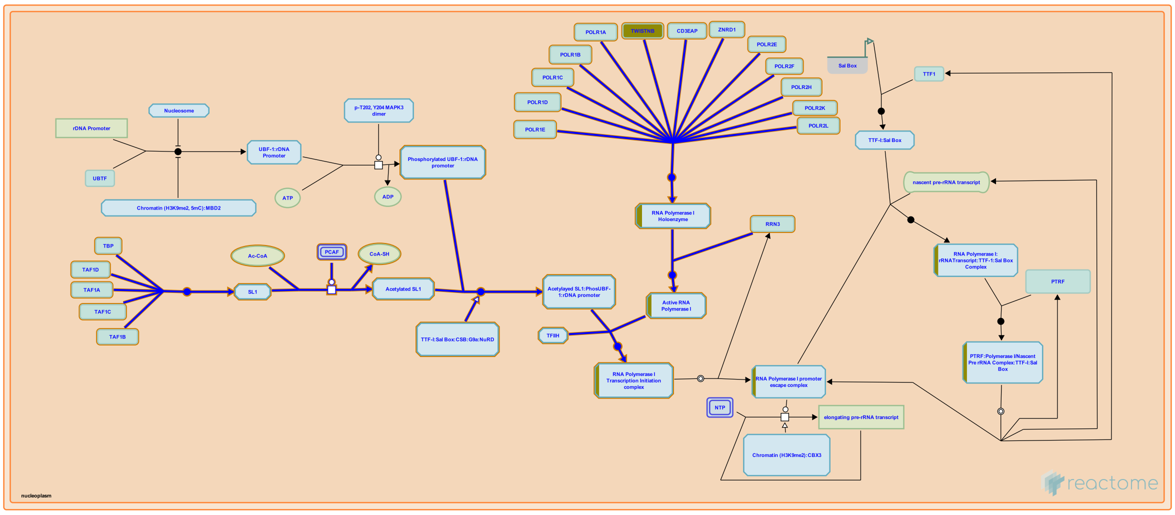

Cellular compartments: nucleolus.

During initiation the double-stranded DNA must be melted and transcription begins. SL1 forms and interacts with UBF-1 and the rDNA promoter. It is this platform that will recruit active RNA polymerase I to the SL1:phosphorlated UBF-1:rDNA promoter complex.

Mammalian rRNA genes are preceded by a terminator element that is recognized by the SL1 complex. This SL1 modulated acetylation of the basal Pol I transcription machinery has functional consequences suggesting that the reversible acetylation may be one way to regulate rDNA transcription.

References

Edit history

Date	Action	Author
2003-07-03	Authored	Comai L
2003-07-03	Created	Comai L
2020-11-17	Edited	Gillespie ME
2020-11-20	Modified	Shorser S

Entities found in this pathway (1)

Input	UniProt Id
MMD	Q3B726

17. Extracellular matrix organization (R-HSA-1474244)

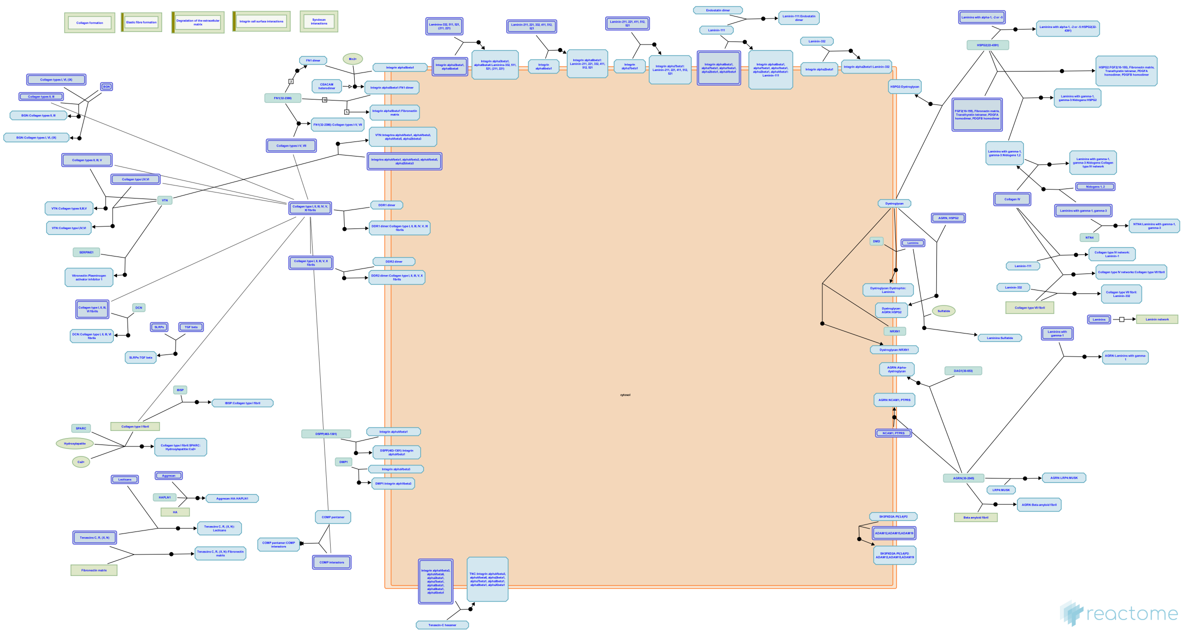

The extracellular matrix is a component of all mammalian tissues, a network consisting largely of the fibrous proteins collagen, elastin and associated-microfibrils, fibronectin and laminins embedded in a viscoelastic gel of anionic proteoglycan polymers. It performs many functions in addition to its structural role; as a major component of the cellular microenvironment it influences cell behaviours such as proliferation, adhesion and migration, and regulates cell differentiation and death (Hynes 2009).

ECM composition is highly heterogeneous and dynamic, being constantly remodeled (Frantz et al. 2010) and modulated, largely by matrix metalloproteinases (MMPs) and growth factors that bind to the ECM influencing the synthesis, crosslinking and degradation of ECM components (Hynes 2009). ECM remodeling is involved in the regulation of cell differentiation processes such as the establishment and maintenance of stem cell niches, branching morphogenesis, angiogenesis, bone remodeling, and wound repair. Redundant mechanisms modulate the expression and function of ECM modifying enzymes. Abnormal ECM dynamics can lead to deregulated cell proliferation and invasion, failure of cell death, and loss of cell differentiation, resulting in congenital defects and pathological processes including tissue fibrosis and cancer.

Collagen is the most abundant fibrous protein within the ECM constituting up to 30% of total protein in multicellular animals. Collagen provides tensile strength. It associates with elastic fibres, composed of elastin and fibrillin microfibrils, which give tissues the ability to recover after stretching. Other ECM proteins such as fibronectin, laminins, and matricellular proteins participate as connectors or linking proteins (Daley et al. 2008).

Chondroitin sulfate, dermatan sulfate and keratan sulfate proteoglycans are structural components associated with collagen fibrils (Scott & Haigh 1985; Scott & Orford 1981), serving to tether the fibril to the surrounding matrix. Decorin belongs to the small leucine-rich repeat proteoglycan family (SLRPs) which also includes biglycan, fibromodulin, lumican and asporin. All appear to be involved in collagen fibril formation and matrix assembly (Ameye & Young 2002).

ECM proteins such as osteonectin (SPARC), osteopontin and thrombospondins -1 and -2, collectively referred to as matricellular proteins (reviewed in Mosher & Adams 2012) appear to modulate cell-matrix interactions. In general they induce de-adhesion, characterized by disruption of focal adhesions and a reorganization of actin stress fibers (Bornstein 2009). Thrombospondin (TS)-1 and -2 bind MMP2. The resulting complex is endocytosed by the low-density lipoprotein receptor-related protein (LRP), clearing MMP2 from the ECM (Yang et al. 2001).

Osteopontin (SPP1, bone sialoprotein-1) interacts with collagen and fibronectin (Mukherjee et al. 1995). It also contains several cell adhesive domains that interact with integrins and CD44.

Aggrecan is the predominant ECM proteoglycan in cartilage (Hardingham & Fosang 1992). Its relatives include versican, neurocan and brevican (Iozzo 1998). In articular cartilage the major non-fibrous macromolecules are aggrecan, hyaluronan and hyaluronan and proteoglycan link protein 1 (HAPLN1). The high negative charge density of these molecules leads to the binding of large amounts of water (Bruckner 2006). Hyaluronan is bound by several large proteoglycans proteoglycans belonging to the hyalectan family that form high-molecular weight aggregates (Roughley 2006), accounting for the turgid nature of cartilage.

The most significant enzymes in ECM remodeling are the Matrix Metalloproteinase (MMP) and A disintegrin and metalloproteinase with thrombospondin motifs (ADAMTS) families (Cawston & Young 2010). Other notable ECM degrading enzymes include plasmin and cathepsin G. Many ECM proteinases are initially present as precursors, activated by proteolytic processing. MMP precursors include an amino prodomain which masks the catalytic Zn-binding motif (Page-McCawet al. 2007). This can be removed by other proteinases, often other MMPs. ECM proteinases can be inactivated by degradation, or blocked by inhibitors. Some of these inhibitors, including alpha2-macroglobulin, alpha1-proteinase inhibitor, and alpha1-chymotrypsin can inhibit a large variety of proteinases (Woessner & Nagase 2000). The tissue inhibitors of metalloproteinases (TIMPs) are potent MMP inhibitors (Brew & Nagase 2010).

References

Frantz C, Stewart KM & Weaver VM (2010). The extracellular matrix at a glance. J Cell Sci, 123, 4195-200.

Lu P, Takai K, Weaver VM & Werb Z (2011). Extracellular matrix degradation and remodeling in development and disease. Cold Spring Harb Perspect Biol, 3.

Bosman FT & Stamenkovic I (2003). Functional structure and composition of the extracellular matrix. J Pathol, 200, 423-8.

Edit history

Date	Action	Author
2011-08-05	Created	Jupe S
2011-09-09	Authored	Jupe S
2012-02-21	Edited	Jupe S
2012-02-28	Reviewed	D'Eustachio P
2013-05-21	Reviewed	Venkatesan N
2013-05-22	Reviewed	Ricard-Blum S

Date	Action	Author
2020-11-20	Modified	Shorser S

Entities found in this pathway (2)

Input	UniProt Id	Input	UniProt Id
ADAMTS9	Q9P2N4	MMD	P26012

18. mRNA Splicing - Minor Pathway (R-HSA-72165)

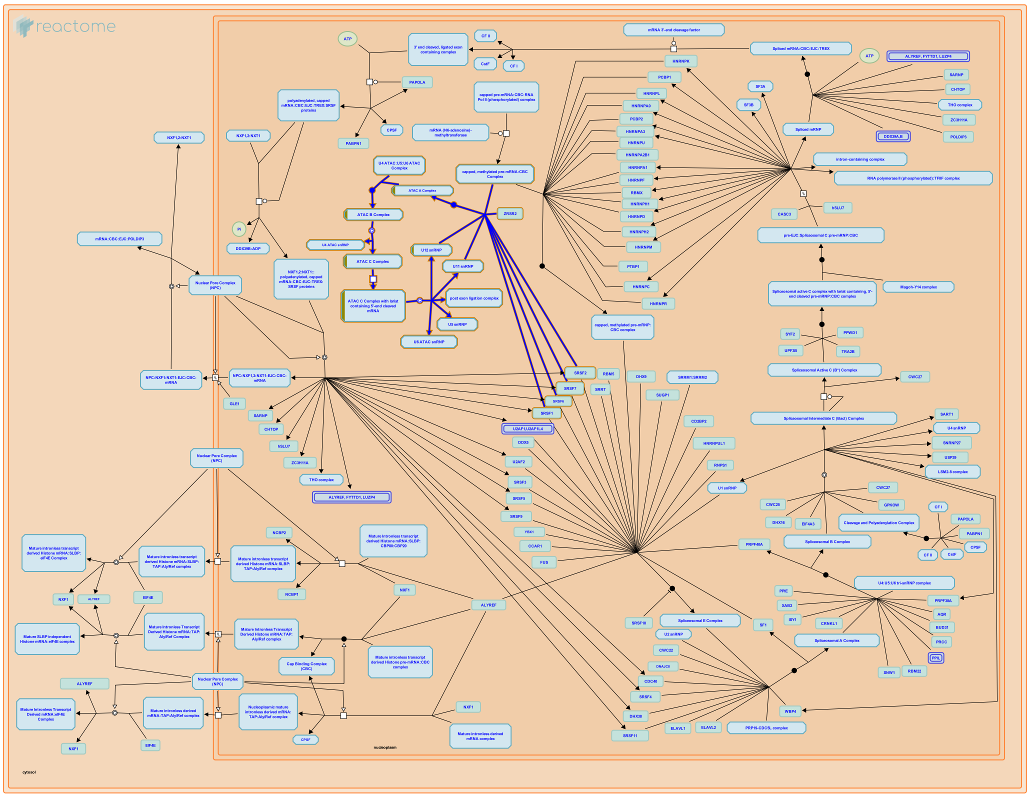

Cellular compartments: nucleoplasm.

The splicing of a subset of pre-mRNA introns occurs by a second pathway, designated the AT-AC or U12-dependent splicing pathway. AT-AC introns have highly conserved, non-canonical splice sites that are removed by the AT-AC spliceosome, which contains distinct snRNAs (U11, U12, U4atac, U6atac) that are structurally and functionally analogous to the major spliceosome. U5 snRNA as well as many of the protein factors appear to be conserved between the two spliceosomes.

References

Tarn WY & Steitz JA (1997). Pre-mRNA splicing: the discovery of a new spliceosome doubles the challenge. Trends Biochem Sci, 22, 132-7.

Wu Q & Krainer AR (1999). AT-AC pre-mRNA splicing mechanisms and conservation of minor introns in voltage-gated ion channel genes. Mol Cell Biol, 19, 3225-36.

Edit history

Date	Action	Author
2003-08-22	Created	Hastings ML
2020-11-17	Edited	Joshi-Tope G
2020-11-20	Modified	Shorser S

Entities found in this pathway (1)

Input	UniProt Id
BAHD1	Q96LT9

19. E3 ubiquitin ligases ubiquitinate target proteins (R-HSA-8866654)

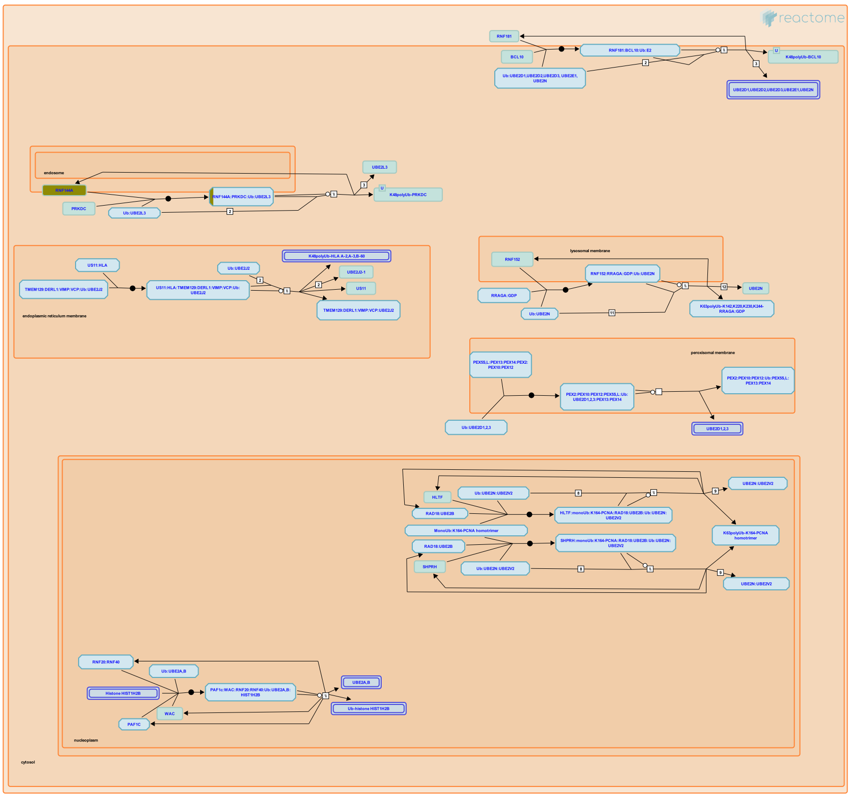

E3 ubiquitin ligases catalyze the transfer of an ubiquitin from an E2-ubiquitin conjugate to a target protein. Generally, ubiquitin is transferred via formation of an amide bond to a particular lysine residue of the target protein, but ubiquitylation of cysteine, serine and threonine residues in a few targeted proteins has also been demonstrated (reviewed in McDowell and Philpott 2013, Berndsen and Wolberger 2014). Based on protein homologies, families of E3 ubiquitin ligases have been identified that include RING-type ligases (reviewed in Deshaies et al. 2009, Metzger et al. 2012, Metzger et al. 2014), HECT-type ligases (reviewed in Rotin et al. 2009, Metzger et al. 2012), and RBR-type ligases (reviewed in Dove et al. 2016). A subset of the RING-type ligases participate in CULLIN-RING ligase complexes (CRLs which include SCF complexes, reviewed in Lee and Zhou 2007, Genschik et al. 2013, Skaar et al. 2013, Lee et al. 2014).

Some E3-E2 combinations catalyze mono-ubiquitination of the target protein (reviewed in Nakagawa and Nakayama 2015). Other E3-E2 combinations catalyze conjugation of further ubiquitin monomers to the initial ubiquitin, forming polyubiquitin chains. (It may also be possible for some E3-E2 combinations to preassemble polyubiquitin and transfer it as a unit to the target protein.) Ubiquitin contains several lysine (K) residues and a free alpha amino group to which further ubiquitin can be conjugated. Thus different types of polyubiquitin are possible: K11 linked polyubiquitin is observed in endoplasmic reticulum-associated degradation (ERAD), K29 linked polyubiquitin is observed in lysosomal degradation, K48 linked polyubiquitin directs target proteins to the proteasome for degradation, whereas K63 linked polyubiquitin generally acts as a scaffold to recruit other proteins in several cellular processes, notably DNA repair (reviewed in Komander et al. 2009).

References

Berndsen CE & Wolberger C (2014). New insights into ubiquitin E3 ligase mechanism. Nat. Struct. Mol. Biol., 21, 301-7.

Deshaies RJ & Joazeiro CA (2009). RING domain E3 ubiquitin ligases. Annu Rev Biochem, 78, 399-434.

Metzger MB, Hristova VA & Weissman AM (2012). HECT and RING finger families of E3 ubiquitin ligases at a glance. J. Cell. Sci., 125, 531-7.

Metzger MB, Pruneda JN, Klevit RE & Weissman AM (2014). RING-type E3 ligases: master manipulators of E2 ubiquitin-conjugating enzymes and ubiquitination. Biochim. Biophys. Acta, 1843, 47-60.

Rotin D & Kumar S (2009). Physiological functions of the HECT family of ubiquitin ligases. Nat. Rev. Mol. Cell Biol., 10, 398-409.

Edit history

Date	Action	Author
2016-04-02	Edited	May B
2016-04-02	Authored	May B
2016-04-02	Created	May B
2016-11-03	Reviewed	Azevedo JE
2020-11-24	Modified	Shorser S

Entities found in this pathway (1)

Input	UniProt Id
RNF144A	P50876

20. B-WICH complex positively regulates rRNA expression (R-HSA-5250924)

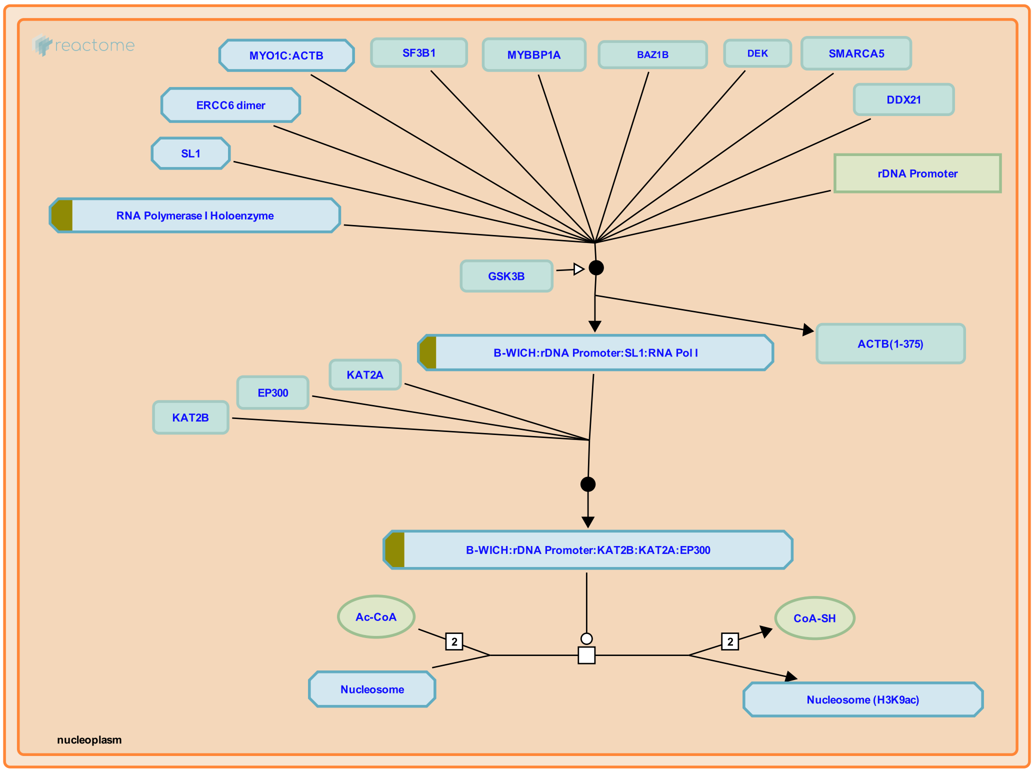

Cellular compartments: nucleoplasm.

The B-WICH complex is a large 3 Mdalton complex containing SMARCA5 (SNF2H), BAZ1B (WSTF), ERCC6 (CSB), MYO1C (Nuclear myosin 1c), SF3B1, DEK, MYBBP1A, and DDX21 (Cavellan et al. 2006, Percipalle et al. 2006, Vintermist et al. 2001, Sarshad et al. 2013, Shen et al. 2013, reviewed in Percipalle and Farrants 2006). B-WICH is found at active rRNA genes as well as at 5S rRNA and 7SL RNA genes. B-WICH appears to remodel chromatin and recruit histone acetyltransferases that modify histones to transcriptionally active states.

References

Cavellán E, Asp P, Percipalle P & Farrants AK (2006). The WSTF-SNF2h chromatin remodeling complex interacts with several nuclear proteins in transcription. J. Biol. Chem., 281, 16264-71.

Sarshad A, Sadeghifar F, Louvet E, Mori R, Böhm S, Al-Muzzaini B, ... Percipalle P (2013). Nuclear myosin 1c facilitates the chromatin modifications required to activate rRNA gene transcription and cell cycle progression. PLoS Genet., 9, e1003397.

Percipalle P, Fomproix N, Cavellán E, Voit R, Reimer G, Krüger T, ... Farrants AK (2006). The chromatin remodelling complex WSTF-SNF2h interacts with nuclear myosin 1 and has a role in RNA polymerase I transcription. EMBO Rep., 7, 525-30.

Vintermist A, Böhm S, Sadeghifar F, Louvet E, Mansén A, Percipalle P & Ostlund Farrants AK (2011). The chromatin remodelling complex B-WICH changes the chromatin structure and recruits histone acetyl-transferases to active rRNA genes. PLoS ONE, 6, e19184.

Shen M, Zhou T, Xie W, Ling T, Zhu Q, Zong L, ... Tao W (2013). The chromatin remodeling factor CSB recruits histone acetyltransferase PCAF to rRNA gene promoters in active state for transcription initiation. PLoS ONE, 8, e62668.

Edit history

Date	Action	Author
2014-01-29	Edited	May B
2014-01-29	Authored	May B
2014-01-31	Created	May B
2015-11-07	Reviewed	Percipalle P
2020-11-20	Modified	Shorser S

Entities found in this pathway (1)

Input	UniProt Id
MMD	Q3B726

21. RNA Polymerase I Promoter Escape (R-HSA-73772)

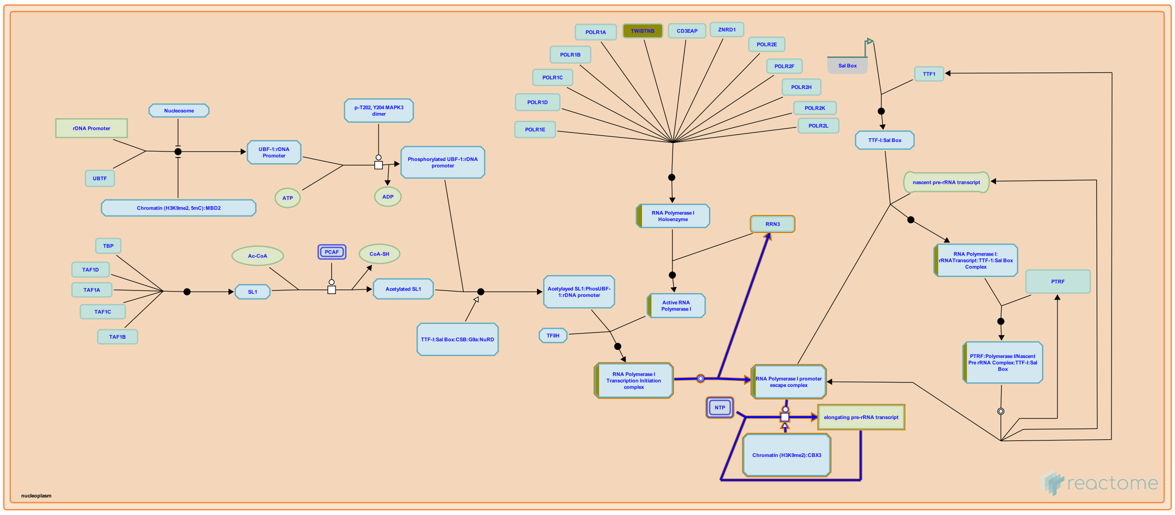

Cellular compartments: nucleolus.

As the active RNA Polymerase I complex leaves the promoter Rrn3 dissociates from the complex. RNA polymerase I Promoter Clearance is complete and Chain Elongation begins (Milkereit and Tschochner, 1998).
The assembly of the initiation complex on the promoter and the transition from a closed to an open complex is then followed by promoter clearance and transcription elongation by RNA Pol I. Unlike the RNA polymerase II system, RNA polymerase I transcription does not require a form of energy such as ATP for initiation and elongation. Regulatory mechanisms operating at both the level of transcription initiation and elongation probably concurrently to adjust the level of rRNA synthesis to the need of the cell.

References

Milkereit P & Tschochner H (1998). A specialized form of RNA polymerase I, essential for initiation and growth-dependent regulation of rRNA synthesis, is disrupted during transcription. EMBO J., 17, 3692-703.

Peyroche G, Milkereit P, Bischler N, Tschochner H, Schultz P, Sentenac A, ... Riva M (2000). The recruitment of RNA polymerase I on rDNA is mediated by the interaction of the A43 subunit with Rrn3. EMBO J., 19, 5473-82.

Edit history

Date	Action	Author
2003-07-03	Authored	Comai L
2003-07-03	Created	Comai L
2020-11-17	Edited	Gillespie ME
2020-11-20	Modified	Shorser S

Entities found in this pathway (1)

Input	UniProt Id
MMD	Q3B726

22. Ion transport by P-type ATPases (R-HSA-936837)

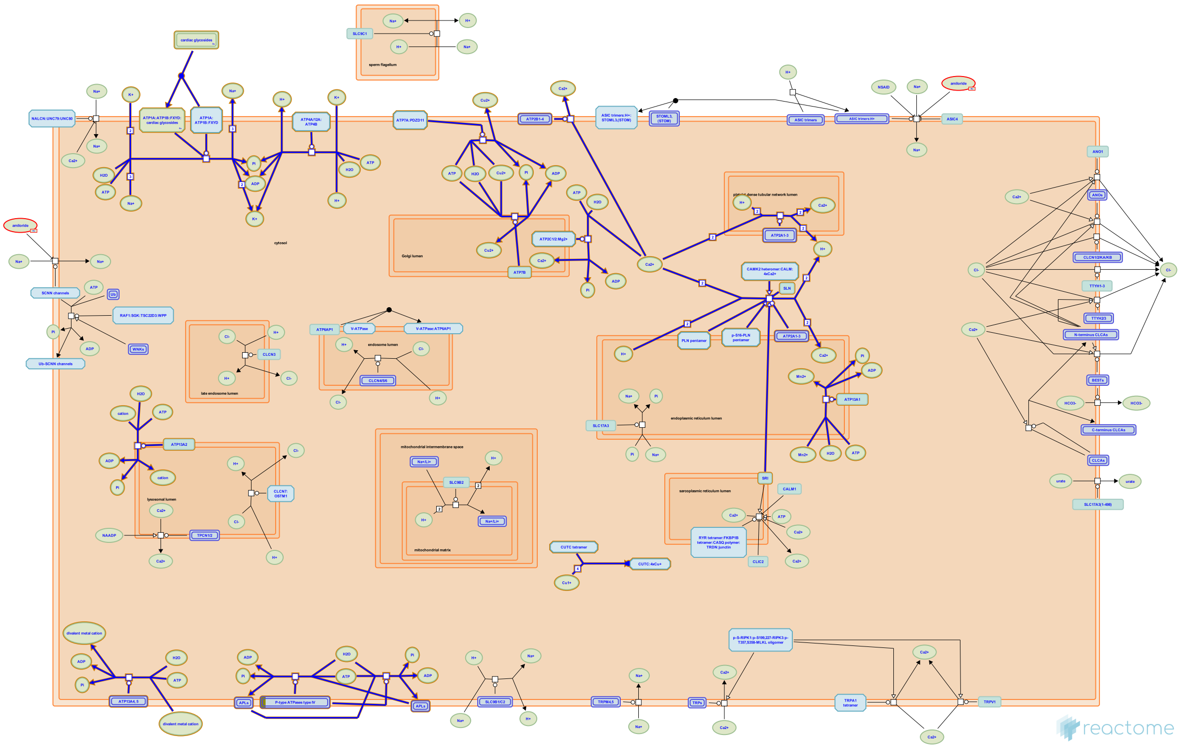

The P-type ATPases (E1-E2 ATPases) are a large group of evolutionarily related ion pumps that are found in bacteria, archaea and eukaryotes. They are referred to as P-type ATPases because they catalyze auto-phosphorylation of a key conserved aspartate residue within the pump. They all appear to interconvert between at least two different conformations, E1 and E2. Most members of this transporter family pump a large variety of cations (Kuhlbrandt W, 2004).

References

Kühlbrandt W (2004). Biology, structure and mechanism of P-type ATPases. Nat Rev Mol Cell Biol, 5 , 282-95.

Edit history

Date	Action	Author
2010-08-24	Edited	Jassal B
2010-08-24	Authored	Jassal B
2010-08-24	Created	Jassal B
2010-11-15	Reviewed	He L
2014-04-07	Revised	Jassal B
2020-11-20	Modified	Shorser S

Entities found in this pathway (1)

Input	UniProt Id
NEO1	O43861

23. Diseases associated with O-glycosylation of proteins (R-HSA-3906995)

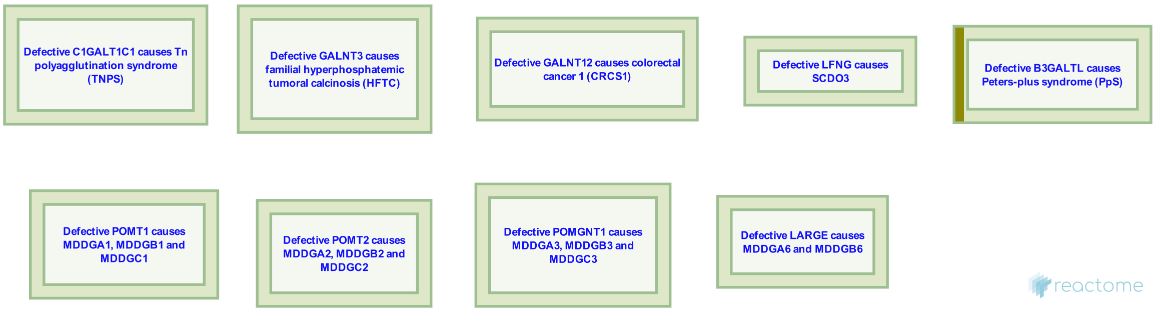

Diseases: congenital disorder of glycosylation.

Glycosylation is the most abundant modification of proteins, variations of which occur in all living cells. Glycosylation can be further categorized into N-linked (where the oligosaccharide is conjugated to Asparagine residues) and O-linked glycosylation (where the oligosaccharide is conjugated to Serine, Threonine and possibly Tyrosine residues). Within the family of O-linked glycosylation, the oligosaccharides attached can be further categorized according to their reducing end residue: GalNAc (often described as mucin-type, due to the abundance of this type of glycosylation on mucins), Mannose and Fucose. This section reviews currently known congenital disorders of glycosylation associated with defects of protein O-glycosylation (Cylwik et al. 2013, Freeze et al. 2014).

References

Cylwik B, Lipartowska K, Chrostek L & Gruszewska E (2013). Congenital disorders of glycosylation. Part II. Defects of protein O-glycosylation. Acta Biochim. Pol., 60, 361-8.

Freeze HH, Chong JX, Bamshad MJ & Ng BG (2014). Solving glycosylation disorders: fundamental approaches reveal complicated pathways. Am. J. Hum. Genet., 94, 161-75.

Edit history

Date	Action	Author
2013-07-17	Edited	Jassal B
2013-07-17	Authored	Jassal B
2013-07-17	Created	Jassal B
2015-12-18	Reviewed	Hansen L, Joshi HJ
2016-07-30	Modified	Gillespie ME

Entities found in this pathway (1)

Input	UniProt Id
ADAMTS9	Q9P2N4

24. Positive epigenetic regulation of rRNA expression (R-HSA-5250913)

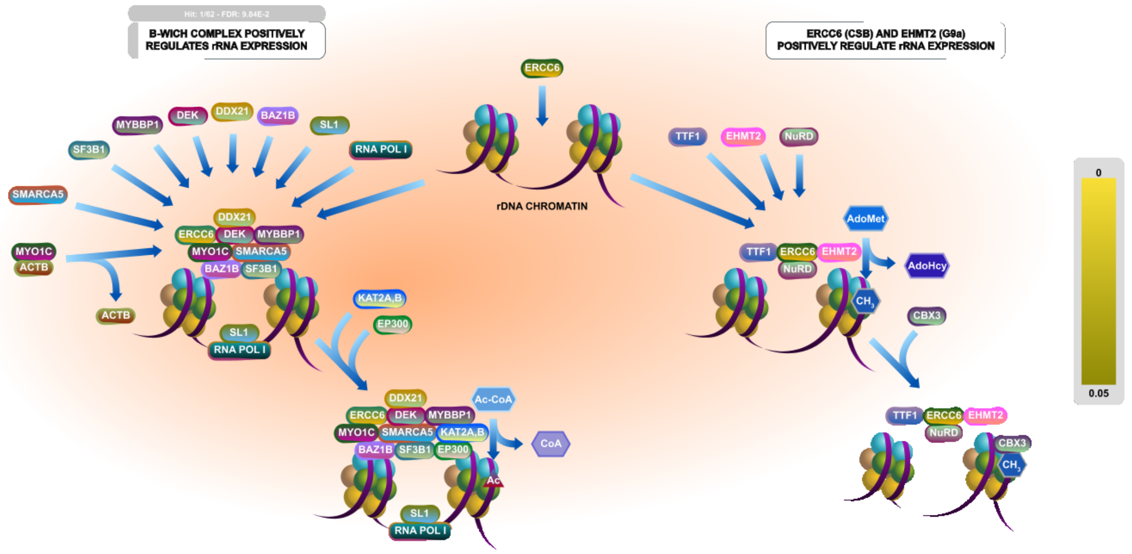

Cellular compartments: nucleoplasm.

Transcription of rRNA genes is controlled by epigenetic activation and repression according to the metabolic requirements of the cell (reviewed in Percipalle and Farrants 2006, McStay and Grummt 2008, Goodfellow and Zomerdijk 2012, Grummt and Langst 2013). Depending on the growth state of the cell, about half of the approximately 400 rRNA genes are expressed and these have the modifications characteristic of active chromatin: unmethylated DNA and acetylated histones. Repressed genes generally have methylated DNA and histone H3 methylated at lysine-9. Regulators of activation include ERCC6 (CSB), histone acetylases such as KAT2B (PCAF), and the B-WICH complex. Dysregulation of RNA polymerase I transcription plays a role in disease (reviewed in Hannan et al. 2013).

The B-WICH complex positively regulates rRNA expression by remodeling chromatin and recruiting histone acetyltransferases that modify histones to transcriptionally active states

ERCC6 (CSB) and EHMT2(G9a) positively regulate rRNA expression by ERCC6 recruiting the histone methyltransferase EHMT2 (also known as G9a) which dimethylates histone H3 at lysine-9 within the transcribed regions of rRNA genes.

ERCC6 (CSB) and KAT2B (PCAF) positively regulate rRNA expression by ERCC6 recruiting the histone acetyltransferase KAT2B to the promoter where KAT2B acetylates histone H4 at several lysine residues and histone H3 at lysine-9. The acetylated chromatin facilitates the assembly of RNA polymerase I initiation complex.

References

McStay B & Grummt I (2008). The epigenetics of rRNA genes: from molecular to chromosome biology. Annu Rev Cell Dev Biol, 24, 131-57.

Goodfellow SJ & Zomerdijk JC (2012). Basic mechanisms in RNA polymerase I transcription of the ribosomal RNA genes. Subcell. Biochem., 61, 211-36.

Grummt I & Längst G (2013). Epigenetic control of RNA polymerase I transcription in mammalian cells. Biochim. Biophys. Acta, 1829, 393-404.

Hannan KM, Sanij E, Rothblum LI, Hannan RD & Pearson RB (2013). Dysregulation of RNA polymerase I transcription during disease. Biochim. Biophys. Acta, 1829, 342-60.

Percipalle P & Farrants AK (2006). Chromatin remodelling and transcription: be-WICHed by nuclear myosin 1. Curr. Opin. Cell Biol., 18, 267-74.

Edit history

Date	Action	Author
2014-01-29	Edited	May B
2014-01-29	Authored	May B
2014-01-31	Created	May B
2015-11-07	Reviewed	Percipalle P
2020-11-20	Modified	Shorser S

Entities found in this pathway (1)

Input	UniProt Id
MMD	Q3B726

25. NoRC negatively regulates rRNA expression (R-HSA-427413)

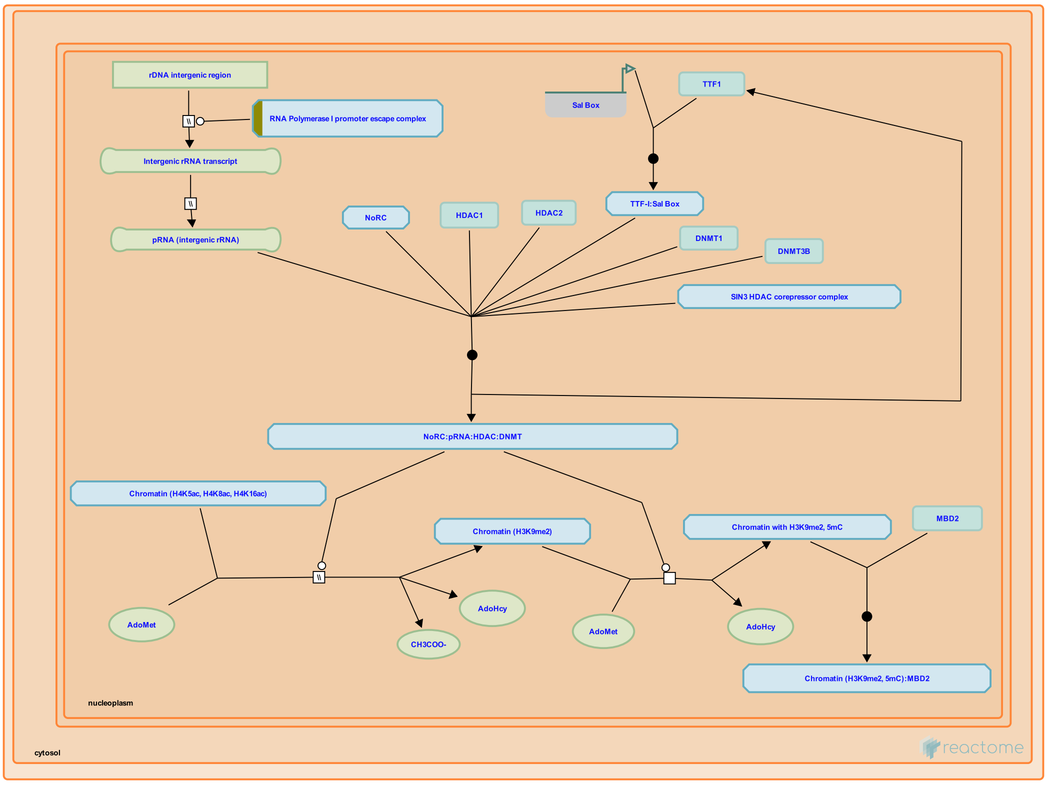

Cellular compartments: nucleoplasm.

The Nucleolar Remodeling Complex (NoRC) comprising TIP5 (BAZ2A) and the chromatin remodeller SNF2H (SMARCA5) silences rRNA gene (reviewed in Santoro and Grummt 2001, Grummt 2007, Preuss and Pikaard 2007, Birch and Zommerdijk 2008, McStay and Grummt 2008, Grummt and Langst 2013). The TAM domain of TIP5 (BAZ2A) binds promoter-associated RNA (pRNA) transcribed from the intergenic spacer region of rDNA. The pRNA bound by TIP5 is required to direct the complex to the main promoter of the rRNA gene possibly by triple helix formation between pRNA and the rDNA. The PHD domain of TIP5 binds histone H4 acetylated at lysine-16. Transcription Termination Factor-I (TTF-I) binds to a promoter-proximal terminator (T0 site) in the rDNA and interacts with the TIP5 subunit of NoRC. NoRC also interacts with the SIN3-HDAC complex, HDAC1, HDAC2, DNMT1, and DNMT3B. DNMT3B interacts with a triple helix formed by pRNA and the rDNA. HDAC1, DNMT1, and DNMT3B have been shown to be required for proper DNA methylation of silenced rRNA gene copies, although the catalytic activity of DNMT3B was not required.

References

McStay B & Grummt I (2008). The epigenetics of rRNA genes: from molecular to chromosome biology. Annu Rev Cell Dev Biol, 24, 131-57.

Grummt I (2007). Different epigenetic layers engage in complex crosstalk to define the epigenetic state of mammalian rRNA genes. Hum Mol Genet, 16, R21-7.

Santoro R & Grummt I (2001). Molecular mechanisms mediating methylation-dependent silencing of ribosomal gene transcription. Mol Cell, 8, 719-25.

Birch JL & Zomerdijk JC (2008). Structure and function of ribosomal RNA gene chromatin. Biochem Soc Trans, 36, 619-24.

Preuss S & Pikaard CS (2007). rRNA gene silencing and nucleolar dominance: insights into a chromosome-scale epigenetic on/off switch. Biochim Biophys Acta, 1769, 383-92.

Edit history

Date	Action	Author
2009-06-19	Authored	May B
2009-06-20	Created	May B
2010-04-06	Edited	May B
2014-02-18	Reviewed	Shiao YH
2020-11-20	Modified	Shorser S

Entities found in this pathway (1)

Input	UniProt Id
MMD	Q3B726

6. Identifiers found

Below is a list of the input identifiers that have been found or mapped to an equivalent element in Reactome, classified by resource.

Entities (8)

Input	UniProt Id	Input	UniProt Id	Input	UniProt Id
A1CF	Q9NQ94	ADAMTS9	Q9P2N4	BAHD1	Q96LT9
GRHL1	Q9NZI5	LRRC8B	Q6P9F7	MMD	Q9P0K1
NEO1	P43146, Q92859	RNF144A	P50876		

Input	Ensembl Id
GRHL1	ENSG00000134317

7. Identifiers not found

These 6 identifiers were not found neither mapped to any entity in Reactome.

ARRDC4	C16orf52	CDR2L	DHX40	MIER3	SSX2IP
