# Supplementary figures and images for: In silico detection of dysregulated genes and molecular pathways in Alzheimer’s disease as basis for food restoring approach
Source: PeerJ. 2025 Apr 7;13:e19100. doi: 10.7717/peerj.19100 (PMC11984471; doi:10.7717/peerj.19100)

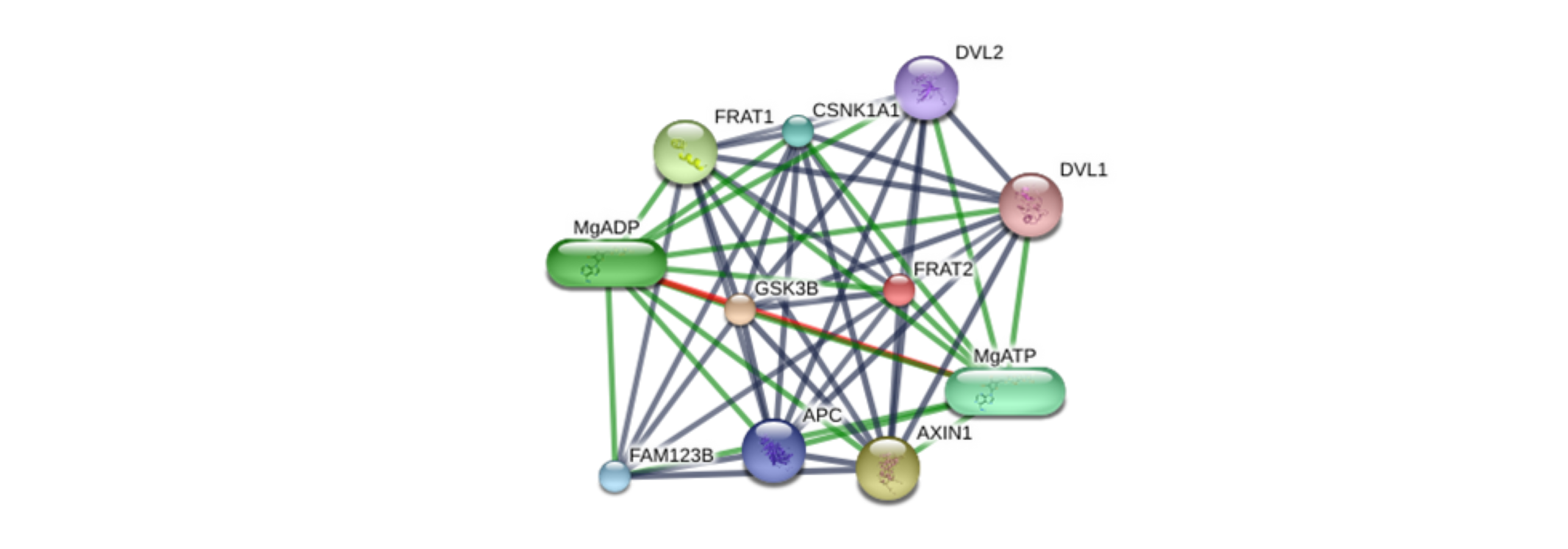

Supplement: Supplemental Information 10 — The analysis was carried out considering Homo sapiens as the organism and a high confidence level of 0.700. Stronger associations are represented by thicker lines. Protein-protein interactions are shown in grey, chemical-protein interactions in green and interactions between chemicals in red. Node size indicates structural knowledge: small nodes correspond to proteins with unknown 3D structure, while large nodes represent proteins with known or predicted 3D structures (http://stitch.embl.de/cgi/network.pl?taskId=kYKGpVE071V4). [file peerj-13-19100-s010.png]

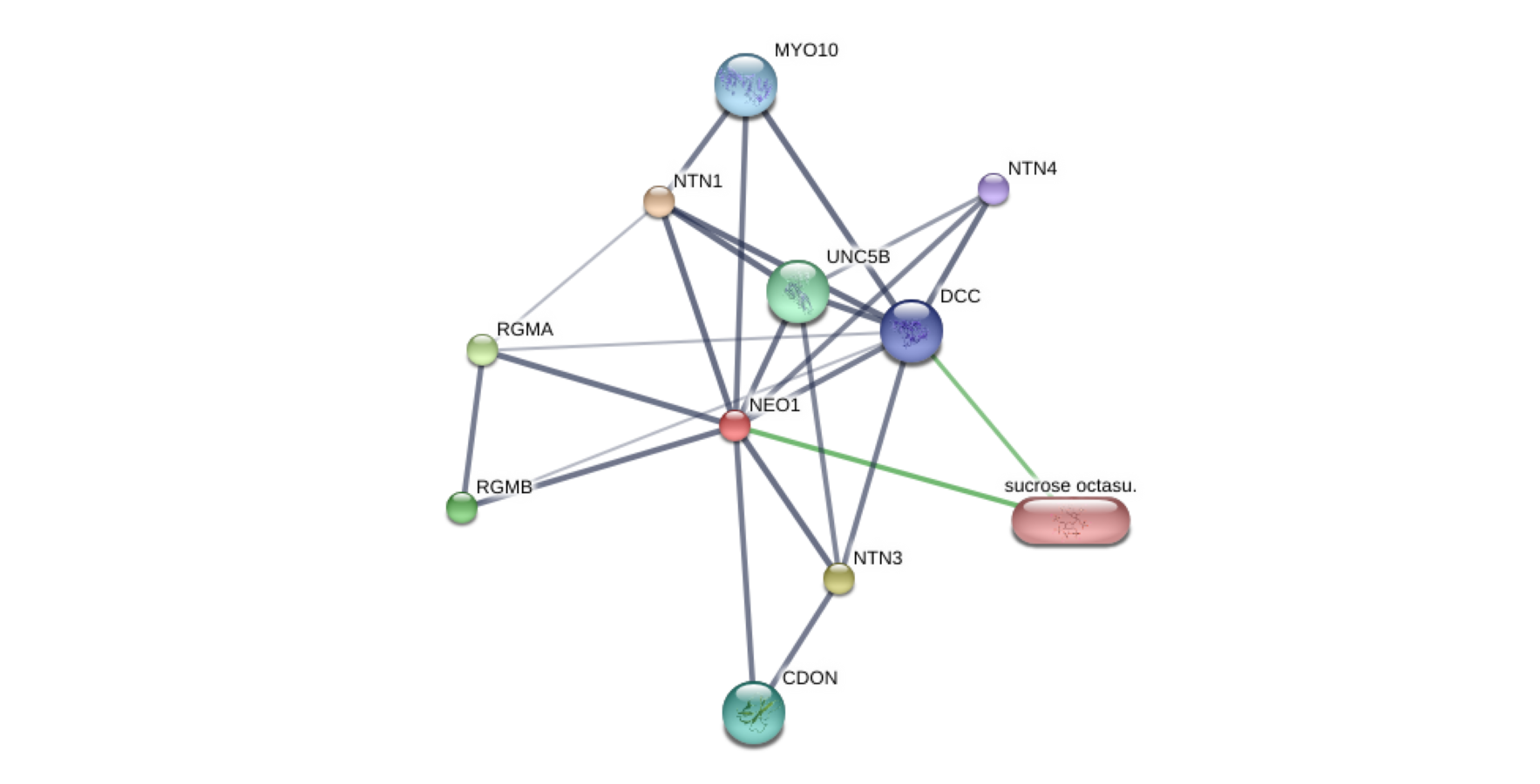

Supplement: Supplemental Information 11 — The analysis was carried out considering Homo sapiens as the organism and a high confidence level of 0.700. Stronger associations are represented by thicker lines. Protein-protein interactions are shown in grey, chemical-protein interactions in green and interactions between chemicals in red. Node size indicates structural knowledge: small nodes correspond to proteins with unknown 3D structure, while large nodes represent proteins with known or predicted 3D structures (http://stitch.embl.de/cgi/network.pl?taskId=qdIkJad1IB3L). [file peerj-13-19100-s011.png]
