# Supplementary material for: In silico detection of dysregulated genes and molecular pathways in Alzheimer’s disease as basis for food restoring approach
Source: PeerJ. 2025 Apr 7;13:e19100. doi: 10.7717/peerj.19100 (PMC11984471; doi:10.7717/peerj.19100)
Supplement: Supplemental Information 12 — Molecular insights related to the BC found to be able to modulate the dysregulated pathways in AD. [file peerj-13-19100-s012.pdf]

Curcumin, a polyphenol from the rhizome of Curcuma Longa L., a flowering plant from Zingiberaceae family, induces neurogenesis by modulation of Wnt/ β-catenin signaling pathway. Tiwari et al., 2016 developed nanoparticles encapsulating curcumin and demonstrated that these nanoparticles can induce hippocampal neurogenesis, neuronal proliferation, and differentiation in an Aβ-induced AD model in rats, through activation of Wnt/β-catenin pathway. C. longa extract significantly reduces the expression of Gsk3ß mRNA and protein in a dose-and time-dependent manner, in an in vitro study (X. Zhang et al., 2011). Curcumin increases β-catenin mRNA and protein, as well as the transcription factor Cyclin D1, in a dose-dependent manner, activating the Wnt-pathway.

Curcumin clinical trials showed controversial results. Thota et al., 2020, demonstrated that curcumin supplementation significantly reduced circulating Gsk-3β. Conversely, Baum et al., 2008, evidenced no significant differences in Mini-Mental State Examination scores or plasma Aβ40 levels over 6 months of observation, and also no adverse effects in AD patients. However, the authors emphasised the importance of further studies with longer periods of observation and, conversely, with additional endpoints.

Osthole is derived from coumarin found in different herbaceous plants such as Cnidium monnieri (L.) Cusson ex Juss and Angelica pubescens Maxim, widely used in traditional Chinese medicine. Yao et al., 2015 suggested that osthole treatment (100 μmol/L) enhances the APP-overexpressing neural stem cells (APP-NSCs) proliferation and their differentiation into neurons and β-catenin mRNA expression, while it decreases the apoptosis and the accumulation of Aβ-42 and Gsk3ß activity. By activating the Wnt-β catenin pathway, it increases the proliferation of neural stem cells, neuronal differentiation and inhibits neuronal apoptosis. Potentially, it plays a role in prevention and treatment of AD (Singh & Bhatti, 2023).

Puerarin is an isoflavone compound derived from Pueraria lobata (Willd.) Ohwi, used in traditional Chinese medicine. Accumulating evidence has indicated that puerarin has multiple pharmacological effects and exhibits potential treatment for various neurological diseases (X. Liu et al., 2023). Yao et al., 2017 demonstrated that puerarin attenuates the hyperphosphorylation of tau protein induced by Aβ1-42 in SH-SY5Y human cells through the inhibition of Gsk3ß expression, induction of β-catenin and cyclin D1 expression and subsequent activation of the Wnt/β-catenin pathway.

Xanthoceraside is another herbal molecule (triterpenoid saponin) extracted from the pericarp of Xanthoceras sorbifolium Bunge. In the hippocampus of APP/PS1 mice, the positive effect of xanthoceraside could be attributed to the increase of Wnt3a expression, the enhanced translocation of β-catenin to the nucleus and the increase levels of inactive Gsk3ß. It can also promote the proliferation and differentiation of neural stem cells into neurons, thus improving learning and memory impairment in transgenic mice (Zhu et al., 2018).

Sulforaphane is a phytocompound belonging to the isothiocyanate family. It is present in sprouts of many cruciferous vegetables like cabbage, broccoli, cauliflower, and brussels sprouts. Sulforaphane is produced by the conversion of glucoraphanin through the enzyme myrosinase, which leads to the formation of this isothiocyanate. It is characterized by antioxidant, anti-inflammatory and anti-apoptotic properties (Panjwani et al., 2018). Several studies have demonstrated the effects of sulforaphane against neurodegeneration (Alfieri et al., 2013; Ping et al., 2010). Han et al., 2017, have highlighted how this molecule leads to up-regulation of Wnt pathway proteins, including β-catenin and cyclin D, promoting proliferation and differentiation of neural stem cells into neurons (Han et al., 2017; Schepici et al., 2020).

Andrographolide is a labdane diterpenoid extracts from the leaves of Andrographis paniculata (Burm.f.) Wall ex Nees, a plant traditionally used in China and India as a natural remedy for inflammatory process.

Tapia-Rojas et al., 2015, showed that andrographolide is a potent activator of Wnt signaling by inducing the transcription of Wnt targets genes, inhibiting Gsk3ß and increasing the level of β-catenin expression. Salvianolic acid A is one of most important polyphenolic components extracted from the roots of Salvia miltiorrhiza Bunge (also known as Danshen), a traditional Chinese herb. Studies have shown that **Salvianolic acid A** can have a neuroprotective effect (Ling et al., 2021; Song et al., 2019). Animal studies highlighted how the administration of Salvianolic acid A significantly enhanced the proliferation, migration and differentiation of neural stem/progenitor cells (NPCs) into neurons. The induced neurogenesis was correlated with the activation of the Wnt3a/Gsk3ß/β-catenin signaling pathways and downstream target genes (Chien et al., 2016; S. Zhang et al., 2022).

Resveratrol is a naturally available polyphenolic compound with antioxidant, anti-cancerous, anti-inflammatory, and anti-aging properties (Albuquerque et al., 2015; Gambini et al., 2015). It is a polyphenol plant secondary metabolite commonly found in grapevines and grape juice. Peanuts, pomegranate, spinach and banana as well contain high concentrations of resveratrol (Quadros Gomes et al., 2018). Surya et al., 2023 present new findings regarding how resveratrol regulates hippocampal neurogenesis through the Wnt signaling pathway, offering potential implications for neurotherapy. They propose that resveratrol could modulate Wnt signaling in several ways, potentially by elevating Sirtuin 1 (SIRT1) levels. This elevated SIRT1 activity may act in Dvl to prevent the degradation of β-catenin from proteasomal degradation independently of the Wnt ligand (Holloway et al., 2010). Additionally, through deacetylation, SIRT1 could facilitate the movement of β-catenin into the cell nucleus, thereby promoting the transcription of Wnt target genes, independently of the ligand. Palomera-Avalos et al., 2017 demonstrated how resveratrol inhibits the activity of Gsk3ß, inducing changes in the expression of axin1 and Dvl3, increasing β-catenin levels, thereby restoring Wnt/β-catenin pathway and thus exerting a neuroprotective role in mouse models (SAMP8- Senescence-accelerated prone mice P8). It has been noted that the administration of resveratrol in clinical trials for patients with AD and stroke yielded positive outcomes (Berman et al., 2017).

One of the first clinical studies in mild to moderate AD evaluating the effects of resveratrol was conducted by Turner et al., 2015. This randomized, double-blinded, placebo-controlled phase II study revealed that resveratrol had safety and good tolerance among patients. Notably, the compound was detectable in the cerebrospinal fluid (CSF), indicating its ability to penetrate the blood-brain barrier, even if the study did not find conclusive evidence of neuroprotective benefits. However, the same research group analyzed sample of CSF and plasma from a subset of AD patients with CSF Aβ42 concentrations < 600 ng/mL. Resveratrol exhibited a reduction in the levels of metalloproteinase (MMP) responsible for the reduction of the blood-brain barrier (BBB) integrity, making it permeable and facilitating the infiltration of immune cells and inflammatory molecules into the central nervous system, thereby aggravating inflammation and neuronal damage. This result suggested an increased maintenance of integrity of the BBB and reduced infiltration of immune cells. Moreover, resveratrol exhibited regulatory effects on neuroinflammation, induced adaptative immunity and mitigated the progression of cognitive decline (Moussa et al., 2017).

Ginkgolide B, a terpenoid extracted from the Ginkgo biloba L. can enhance the presence of β-catenin in the cell nucleus, probably by inhibiting Gsk3ß. It activates the Wnt/β-catenin pathway, thus promoting the differentiation of neural stem cells (C. Li et al., 2022; M. Y. Li et al., 2018).

The extract derived from G. biloba L. comprises components like ginkgolides, bilobalide, quercetin and isorhamnetin. These constituents have demonstrated a significant influence on neural cell proliferation, as evidenced by findings from both clinical trials and animal (Ihl et al., 2012; Nada et al., 2014). The results from the study conducted by Mazza et al., 2006 confirmed the clinical efficacy of G. biloba in the dementia of the Alzheimer type, comparable with donepezil clinical efficacy. Conversely, the study

conducted by DeKosky et al., 2008 demonstrated that G. biloba at 120 mg twice a day was not effective in reducing either the overall incidence rate of dementia or AD incidence in elderly individuals with normal cognition or those with Mild Cognitive Impairments (MCI).

All these mentioned natural compounds can up-regulate the Wnt/β-catenin pathway found to be down-regulated in AD.

Regarding Netrin-1 pathway, among the dietary components that allow its modulation, upregulating it, we identified some BC reported in the results and below discussed.

Choline is an essential nutrient needed for the structural integrity and signaling functions of cell membranes, for acetylcholine synthesis (a crucial neurotransmitter for the brain and central nervous system functions, including memory and muscle control) and for methyl group metabolism (Gong et al., 2023). It's an amine that can be synthesized de novo by methylation of phosphatidylethanolamine. Despite de novo synthesis, it's insufficient to fulfill all biological functions. Choline is present in human milk, egg yolks, soy seeds, wheat germ, meat and brewer's yeast (Zeisel & Da Costa, 2009).

Albright et al., 2005 observed that the availability of maternal dietary choline, during mice gestation, can influence the levels of Netrin-1 and DCC. This intake contributes to the modulation of neurogenesis, neuronal migration, cell survival and differentiation during fetal development. Inadequate choline intake during gestation, may lead to neuronal defects at birth and impairment of cognitive postnatal abilities (Y. Wang et al., 2016). Choline supplementation, as demonstrated by several studies conducted on mice, reduces and prevents normal age-related cognitive decline (Velazquez et al., 2020).

Methionine is another compound important for the Netrin1 pathway. It is present in high levels in nuts, beef, lamb, turkey, fish, shellfish, cheese, eggs, dairy products and beans.

Vitamin B6 and B12 and folate are other essential components are which exert their potential neuroprotective effect by maintaining the level of Netrin-1.

Folates and **vitamins B6** and **B12** are essential for homocysteine metabolism (altered in vascular and neurodegenerative diseases), which occurs via remethylation of methionine or transsulfuration to cysteine. In different studies, it was observed that diets that are high in methionine, low in folate and deficient in vitamin B6/B12 have been associated with various issues such as vascular leakage, cerebral vascular dysfunction, short-term memory loss and neurodegeneration (Nuru et al., 2018; Weekman et al., 2019). Mice fed with a diet high in methionine, low in folate and vitamins exhibited a reduction in the expression of the Netrin-1 protein and an increase in the methylation of the Netrin-1 gene promoter, as determined through methylation-sensitive restriction enzyme-polymerase chain reaction analysis (Kalani et al., 2019). Data suggests that the reduction in Netrin-1 expression resulting from the hypermethylation of its gene could be linked to memory loss (Nuru et al., 2018). The link between Netrin-1 and memory was established by administering netrin, which significantly restored long-term fear-motivated memory. As reported in the results, we identified those altered miRs that target genes involved in the two identified pathways of interest (which are Wnt-β-catenin phosphorylation and Netrin-1). These miRs could themselves be modulated by BC. Pogue et al., 2011 revealed that curcumin effectively decreased the levels of miR-146 and miR-125b. In primary neurons, elevated expression of miR-125b leads to Tau phosphorylation and an increase in p53, cdk5 and p44/42-MAPK signaling (Banzhaf-Strathmann et al., 2014). Moreover, the increased expression of miR-125b led to a notable elevation in the levels of APP and β-secretase 1 (BACE1), contributing to the production of Aβ peptide (Jin et al., 2018). From the enrichment analysis we conducted by MIENTURNET, miR-125b-5p was found up-regulated in AD, targeting the Neogenin-1 (NEO1) gene, which is involved in the Netrin-1 pathway. NEO1 is a versatile transmembrane receptor that is involved in axonal guidance, neuronal differentiation, morphogenesis and cell death (Wilson & Key, 2007). Curcumin-induced downregulation of miR-125b could potentially influence the expression of NEO1 and Netrin-1 with impact on pathways

associated with axon growth and neural guidance. However, it’s crucial to consider that molecular biology is complex, and effects can vary based on numerous factors.

MiR-132-3p found down-regulated in AD, is often referred to as “NeurimmiR” due to its involvement in various neurophysiological and pathological processes. Emerging preclinical findings suggest that miR-132 may play a role in the progression of Aβ and tau pathology. Additionally, clinical studies have suggested that reduced circulating levels of miR-132 could act as a potential diagnostic biomarker in AD (Lau et al., 2013; Pichler et al., 2017; M. Zhang & Bian, 2021). The study of Ge et al., 2020 highlighted how berberine treatment could attenuate the neuronal damage in neuronal cells induced by Aβ-25-35, through up-regulation of miR-132-3p expression. The study demonstrated that berberine not only inhibited apoptosis and oxidative stress, but also enhanced synaptic activity and plasticity. Berberine, as a natural alkaloid compound, exhibits a variety of pharmacological effects. In recent years, numerous studies have explored the role of berberine in diseases of the central nervous system such as AD (Cheng et al., 2022; Yuan et al., 2019). The enrichment analysis conducted by MIENTURNET, has revealed that PPP2R5E is a gene target of miR-132-3p. The PPP2R5E gene is important for the phosphorylation of tau protein, and it is involved also in the Wnt/β-catenin pathway. In addition, the berberine-induced up-regulation of miR-132a-3p could potentially influence the expression of PPP2R5E gene and the pathways where it is involved in. For this reason, modulation of miR-132-3p through berberine administration represents a potential therapeutic approach in AD. On the other hand, a recent study has revealed that miR-22-3p, found to be down-regulated in AD, enhances apoptosis and reduces Aβ deposition (Xia P. et al., 2022). It is suggested that curcumin leads to the upregulation of the above miRNA (miR-22-3p), modulating relevant genes and pathways (FRAT2 and Wnt/β-catenin pathway) in which this miR is involved in.
